# Habitat use of and threats to African large carnivores in a mixed‐use landscape

**DOI:** 10.1111/cobi.13943

**Published:** 2022-08-04

**Authors:** Paolo Strampelli, Philipp Henschel, Charlotte E. Searle, David W. Macdonald, Amy J. Dickman

**Affiliations:** ^1^ Wildlife Conservation Research Unit, Department of Zoology University of Oxford, Recanati‐Kaplan Centre Oxford UK; ^2^ Panthera New York City New York USA; ^3^ Lion Landscapes Iringa Tanzania

**Keywords:** *Acinonyx jubatus*, anthropogenic disturbance, *Crocuta crocuta*, habitat use, *Lycaon pictus*, *Panthera leo*, *Panthera pardus*, trophy hunting area abandonment, abandono de área, caza de trofeos, perturbación antropogénica, uso de hábitat, Acinonyx jubatus, Crocuta crocuta, Lycaon pictus, Panthera leo, Panthera pardus

## Abstract

Large carnivores increasingly inhabit human‐affected landscapes, which exhibit heterogeneity in biotic resources, anthropogenic pressures, and management strategies. Understanding large carnivore habitat use in these systems is critical for their conservation, as is the evaluation of competing management approaches and the impacts of significant land‐use changes. We used occupancy modeling to investigate habitat use of an intact eastern African large carnivore guild across the 45,000 km^2^ Ruaha‐Rungwa landscape in south‐central Tanzania. We determined the relative impact on five large carnivore species of biotic, anthropogenic, and management factors at the scales of home range selection and short‐term use within home ranges. We also specifically tested the effect of abandonment of trophy hunting areas on large carnivore occurrence. Patterns of habitat use differed among species. Lions (*Panthera leo*) appeared affected by top‐down limitation, as their occurrence was significantly negatively associated with illegal human activity (β = –0.63 [SE 0.28]). African wild dogs (*Lycaon pictus*), instead, were limited by biotic features; the species was negatively associated with riverine areas of high sympatric predator density (β = –1.00 [SE 0.43]) and used less‐productive habitats. Spotted hyaena (*Crocuta crocuta*) and leopard (*Panthera pardus*) persisted in more disturbed areas and across habitat types. Large carnivore occurrence was not affected by whether an area was used for photographic or trophy hunting tourism; regular law enforcement was instead a better predictor of occurrence. All species fared better in actively managed hunting areas than those that had been abandoned by operators. Overall, our findings highlight the divergent habitat requirements within large carnivore guilds and the importance of adopting an integrated approach to large carnivore conservation planning in modern systems. We also identified a novel threat to African conservation areas in the form of decreased management investments associated with the abandonment of trophy hunting areas.

## Abstract 2

*Article impact statement*: Habitat degradation associated with ongoing hunting area abandonment is shown to be a novel threat to large African carnivore populations.

## Abstract (es)

Uso de Hábitat y Amenazas para los Grandes Carnívoros de África en un Paisaje de Uso Mixto

**Resumen:**

Cada vez más, los grandes carnívoros habitan paisajes afectados por los humanos, los cuales presentan heterogeneidad en los recursos bióticos, las presiones antropogénicas y las estrategias de manejo. El conocimiento sobre cómo usan el hábitat los grandes carnívoros en estos sistemas es crucial para su conservación, como lo es la evaluación de las estrategias competitivas de manejo y los impactos de los cambios significativos en el uso de suelo. Usamos modelos de ocupación para investigar el uso de hábitat por parte de un gremio intacto de grandes carnívoros en el este de África a lo largo de los 45,000 km^2^ del paisaje Ruaha‐Rungwa en el centro‐sur de Tanzania. Determinamos el impacto relativo que tienen los factores bióticos, antropogénicos y de manejo sobre cinco especies de grandes carnívoros a escala de selección de extensión doméstica y uso a corto plazo dentro de la extensión doméstica. También analizamos específicamente el efecto que tiene el abandono de las áreas de caza de trofeos sobre la presencia de los grandes carnívoros. Los patrones de uso de hábitat difirieron entre las especies. Los leones (*Panthera leo*) parecieron estar afectados por la limitación de arriba‐abajo ya que su presencia estuvo asociada negativamente de manera importante con la actividad humana ilegal (β = – 0.63 [SE 0.28]). El perro salvaje africano (*Lycaon pictus*) estuvo limitado por los elementos bióticos; la especie estuvo asociada negativamente con las áreas de alta densidad simpátrica de depredadores (β = – 1.00 [SE 0.43]) y utilizó los hábitats menos productivos. La hiena moteada (*Crocuta crocuta*) y el leopardo (*Panthera pardus*) persistieron en áreas más perturbadas y en todos los tipos de hábitat. La presencia de los grandes carnívoros no se vio afectada si el área se usaba para caza de trofeos o turismo fotográfico; la aplicación regular de la ley fue un mejor pronóstico de la presencia. A todas las especies les fue mejor en las áreas de caza con gestión activa que en aquellas abandonadas por los operadores. En general, nuestros descubrimientos resaltan los distintos requerimientos de hábitat dentro de los gremios de grandes carnívoros y la importancia de adoptar un enfoque integrado para la planeación de la conservación de estas especies en los sistemas modernos. También identificamos una amenaza nueva para las áreas de conservación africana a manera de inversiones de manejo disminuidas asociadas con el abandono de las áreas de caza de trofeos.

## INTRODUCTION

Modern African conservation landscapes often comprise areas under a range of management strategies, which exhibit varying levels of protection and anthropogenic disturbance and span habitat types and resource gradients (Lockwood et al., 2006; Riggio et al., 2019). Although intact, strictly protected areas (PAs), such as national parks, remain critical for most African large carnivore populations (Balme et al., 2010), many also rely on adjacent multiple‐use areas, where anthropogenic impacts often exert greater pressures. Here, disturbances can include habitat loss to agriculture, competition with livestock, prey‐base depletion, and direct mortality from persecution, bycatch from bushmeat poaching, and trophy hunting (Bauer et al., 2020). Because of their naturally low densities, wide‐ranging nature, and potential for conflict with humans, large carnivores are particularly vulnerable to such impacts (Cardillo et al., 2004). Indeed, as the intensity of anthropogenic pressures has increased over recent decades, large carnivores have experienced severe range contractions across the continent (Wolf & Ripple, 2017).

Despite these trends, it remains poorly understood how anthropogenic pressures compare with biotic resources and interspecific effects in shaping space use and how these relationships vary among species (Balme et al., 2014; Everatt et al., 2019). Understanding the habitat requirements and tolerance limits of large carnivore communities in modern, multidimensional systems is critical for their conservation in increasingly heterogeneous and human‐affected habitats (Balme et al., 2014).

Furthermore, conservation strategies must align with economic development and access to natural resources by local communities to be successful in the long term (Selemani, 2020). Thus, there is also an urgent need to evaluate which models delivering both biodiversity conservation and human development benefits can be successfully integrated within conservation frameworks (Lindsey et al., 2006). Identifying such solutions is particularly important in Tanzania, home to some of Africa's largest remaining wilderness areas (UNEP‐WCMC & IUCN, 2018), but where a growing human population is placing increasing pressure on PAs (Lobora et al., 2017).

Many of Tanzania's PAs are used for trophy hunting, which has a relatively small environmental footprint and can help conserve large carnivores and their habitats by creating value for wildlife in areas often unsuitable for photographic tourism (Dickman et al., 2019; Lindsey et al., 2007). However, if poorly managed or additive to other sources of anthropogenic mortality, trophy hunting can have severe detrimental impacts on populations (Creel et al., 2016; Packer et al., 2011). Tanzania is one of Africa's most popular destinations for trophy hunting (Packer et al., 2011); hunting is permitted in the majority of its PAs, over approximately one‐third of the country (circa 300,000 km^2^; MNRT, 2016, 2018; Riggio et al., 2019). Nevertheless, relatively little research has been carried out on the impact of the practice on large carnivore populations in Tanzania (but see Brink et al. [2016] and Packer et al. [2011]), which has led to increased conservation concern (USFWS, 2015). Furthermore, approximately one‐half of the country's 150 hunting areas were vacated by operators and returned to the government for management from 2014 to 2018, and similar patterns have been observed elsewhere in sub‐Saharan Africa (FZS, 2018). The impacts of this significant land management change on large carnivores are also yet to be assessed.

At approximately 45,000 km^2^, Tanzania's Ruaha‐Rungwa landscape is a vast mosaic of fully and semiprotected areas that span multiple habitats, and is home to a complete eastern African large carnivore guild. The landscape thus permits the investigation of large carnivore spatial ecology in a multiple‐use setting characteristic of modern Africa. As the complex comprises some of Tanzania's largest hunting areas, some of which are now vacant, it also provides the opportunity to assess the impact of both trophy hunting and hunting area vacancy on large carnivore populations.

We used occupancy modeling (Mackenzie et al., 2002) of sign‐based presence‐absence data to determine the relative impact of biotic, anthropogenic, and management factors on habitat use of five large carnivore species in Ruaha‐Rungwa. Ours is the first landscape‐scale assessment of these populations, which, although believed to be globally important, have yet to be assessed at this scale. We considered what our findings reveal about habitat requirements of large carnivore guilds in multiple‐use landscapes and the effectiveness of competing management strategies. We also used our results to provide insights into trophy hunting as a conservation tool in the region and to elucidate the implications of the emerging trend of hunting‐area abandonment in Tanzania and elsewhere.

## METHODS

### Study area

**FIGURE 1:**
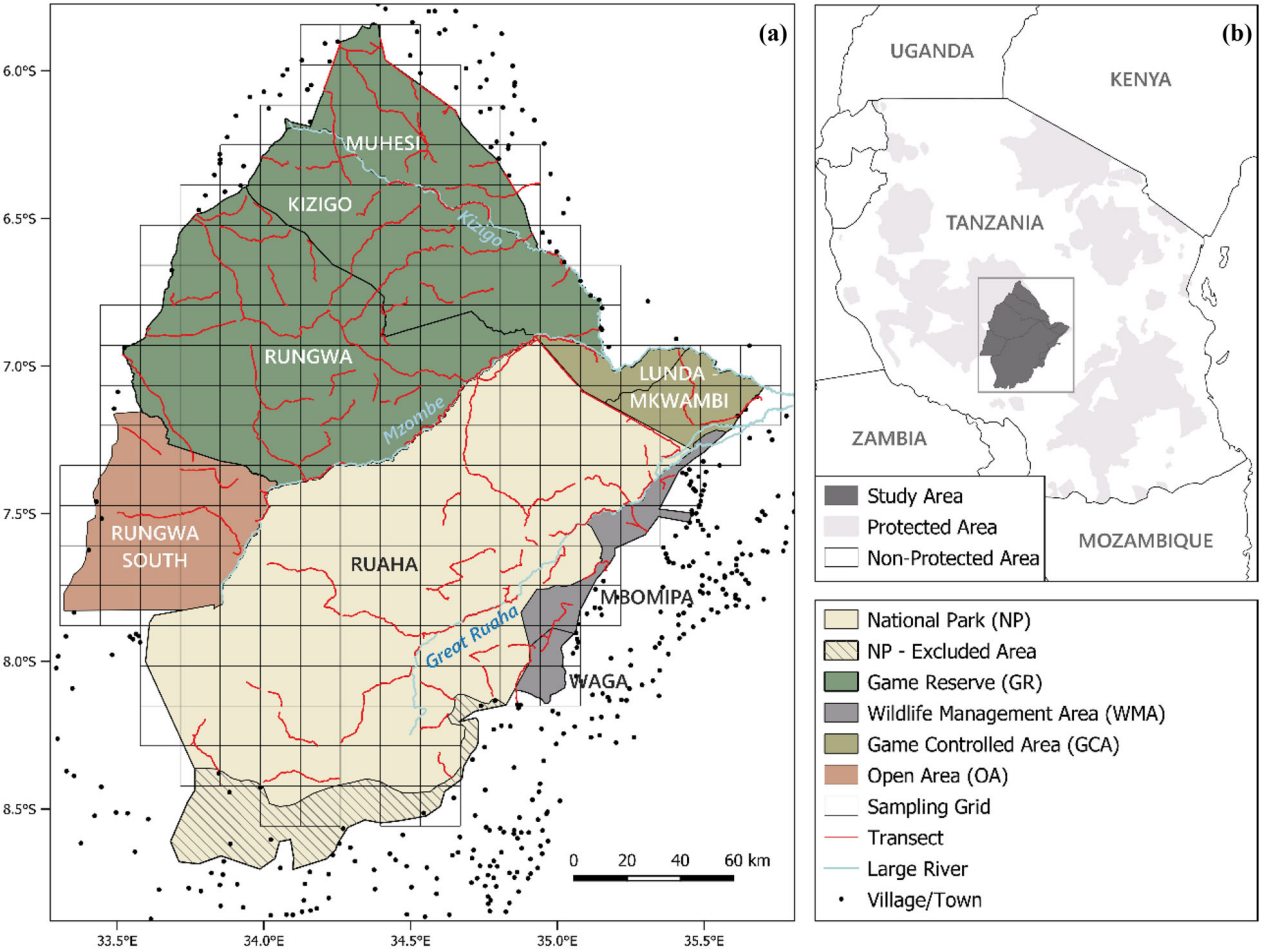
(a) Home‐range‐scale sampling grid and survey transects in the Ruaha‐Rungwa conservation landscape and (b) the landscape within the wider context of Tanzania's protected areas. Both the gazetted and effective boundaries for Ruaha National Park are depicted. Lunda‐Mkwambi Game Controlled Area is composed of Lunda‐Mkwambi North and South, while Rungwa South Open Area (OA) includes both Rungwa South OA and Rungwa Mzombe OA. Only villages near the protected‐area complex are shown.

Ruaha‐Rungwa is a large PA system in south‐central Tanzania, comprising Ruaha National Park (NP) (20,226 km^2^), where only photographic tourism is permitted; Rungwa, Kizigo, and Muhesi Game Reserves (GRs) (9175, 5140, and 2720 km^2^, respectively), where only trophy hunting is permitted; community‐managed Matumizi Bora ya Malihai Idodi na Pawaga (MBOMIPA) and Waga Wildlife Management Areas (WMAs) (947 and 344 km^2^, respectively), where both photographic tourism and trophy hunting are permitted (although neither were taking place at the time of the study); and Lunda‐Mkwambi Game Controlled Area (GCA) (1720 km^2^) and Rungwa South Open Area (OA) (3870 km^2^), where trophy hunting and additional resource extraction by local communities are permitted. The complex is surrounded by unprotected village lands to the south and east and by a number of additional OAs and GCAs to the west and north (Figure 1). No fences restricting the movement of wildlife are present.

Law enforcement is generally higher in the NP and the GRs than in the GCA, OA, and WMAs. Nevertheless, due to boundary disputes, human settlements and intensive agriculture are present in an area of ∼2100 km^2^ in southwestern Ruaha NP (TAWIRI, 2019). As a result, the effective boundaries of the NP do not correspond to the gazetted boundaries in this area (Figure 1); thus, the area was excluded from our study. Other anthropogenic pressures within the PA complex include land clearing for settlements and subsistence agriculture; bushmeat poaching; fishing; burning for honey gathering; legal and illegal logging; and illegal mining (TAWIRI, 2019).

Trophy hunting areas in the complex are subdivided into zones (i.e., hunting blocks), where lion (*Panthera leo*), leopard (*Panthera pardus*), spotted hyaena (*Crocuta crocuta*), and most ungulates are hunted on a quota system (MNRT, 2016). Hunting operators are required to contribute to the management of their respective hunting blocks through protection activities (e.g., antipoaching) (MNRT, 2018); however, four of the 12 blocks within the three GRs (∼33%) were vacant at the time of the study and no trophy hunting took place (map of block vacancy in Appendix S5).

Vegetation cover is composed primarily of a mosaic of *Acacia‐Commiphora* open savannah bushland and central Zambezian and Eastern *Brachystegia‐*dominated miombo woodland, complemented by riverine forests and floodplain grasslands (Olson et al., 2001). Annual precipitation varies from 450 mm in eastern Ruaha NP to 900 mm in Rungwa South OA (Fick & Hijmans, 2017). Surface water availability is highly seasonal; the majority of smaller tributaries dry up completely in the dry season (July–October). The Great Ruaha, Mzombe, and Kizigo Rivers are the largest in the complex (Figure 1) and key sources of dry season surface water. The landscape's large mammal assemblage includes most East African species (TAWIRI, 2019), and the area is believed to harbor globally important populations of lion, cheetah (*Acinonyx jubatus*), African wild dog (*Lycaon pictus*), leopard, spotted hyaena, and striped hyaena (*Hyaena hyaena;* TAWIRI, 2009); however, little research has been carried out into these populations to date.

### Study design

We employed an occupancy modeling framework to model the distribution of large carnivores, their prey, and illegal human activity (see “Site use covariates in occupancy models”), based on presence‐absence sign (“spoor”, or track) transect data, and make inferences on their habitat use. Occupancy models use spatially or temporally replicated detection‐nondetection surveys (i.e., sampling occasions) to estimate the probability of detecting a species (*p*) and derive unbiased probabilities of sites being occupied or used by the species (ψ) while explicitly accounting for imperfect detection (Mackenzie et al., 2002).

Because habitat use is a hierarchical process, ranging from distribution and home range selection to the temporary use of patches within the home range (Johnson, 1980), investigations limited to a single scale may not recognize the importance of key habitat components (Ciarniello et al., 2007; Everatt et al., 2015). We therefore investigated large carnivore habitat use at two biologically meaningful scales: home range selection and persistence, and short‐term use within the home range, equivalent to Johnson's (1980) second and third order of habitat selection, respectively.

To investigate habitat use at the home range scale, we defined sampling sites as 225‐km^2^ grid cells overlaid across the landscape (Figure 1). This size is representative of the scale at which large carnivores make second‐order habitat‐use decisions (Henschel et al., 2016) and is appropriate for decision‐making by PA managers (Petracca et al., 2019). To investigate short‐term use within the home range, we divided our survey transects into 2‐km segments, which we considered representative of the scale at which large carnivores make short‐term habitat‐use decisions (Everatt et al., 2015).

### Data collection

We conducted vehicle‐based spoor transect surveys along roads to collect spatially replicated detection‐nondetection data. The survey team consisted of a driver and two experienced trackers seated on custom‐made seats on the bull‐bar of the vehicle, which was driven at a maximum speed of 10 km/h. Surveying was carried out between dawn and approximately 10:30, before a high‐standing sun made spoor detection difficult.

We recorded spoor data of lion, leopard cheetah, African wild dog, and spotted hyaena, as well as buffalo (*Syncerus caffer*), giraffe (*Giraffa camelopardalis*), plains zebra (*Equus quagga*), eland (*Taurotragus oryx*), sable (*Hippotragus niger*), roan (*Hippotragus equinus*), greater kudu (*Tragelaphus strepsiceros*), and impala (*Aepyceros melampus*). Because of the difficulty associated with differentiating between sable and roan spoor and their similar habitats (Estes, 1991), these were pooled (roan and sable). We also recorded signs of recent illegal human activity (footprints and bicycle or motorcycle trails).

Surveys were carried out over two dry seasons from July 7 to November 29, 2017 and from June 29 to November 21, 2018. Survey transects varied in length based on the amount of roads present within each site, with a minimum 6 km and a maximum 20 km surveyed within each home range site (225 km^2^). Differences in sampling effort between sites were accounted for in the modeling process (Mackenzie et al., 2002).

We employed a spatially replicated occupancy sampling approach (Everatt et al., 2019; Henschel et al., 2016; Petracca et al., 2019). Each transect was divided into 500‐m segments, and we recorded whether a sign of each species was detected (1) or not detected (0) within each segment. Transects were surveyed once; each site was sampled once in one of the two survey seasons.

The probability of detecting tracks depends on the quality of the road (Funston et al., 2010) and how recently it was used by vehicles. We, therefore, graded the quality of each 500‐m segment of the road (from 1 to 4: 1, excellent; 4, poor [Henschel et al., 2016]) and determined how recently it had last been used by a vehicle (from 0 to 3: 0, no vehicles in ≥3 days; 1, no vehicles yesterday; 2, vehicle or vehicles yesterday; 3, vehicle or vehicles today [transect was ended if a score of 3 was assigned]).

### Site use covariates in occupancy models

We identified a range of predictor variables (covariates) to explain heterogeneity in large carnivore occurrence. Covariates were either biotic (prey availability, vegetation type, and landscape features) or abiotic (anthropogenic disturbances and management). Prey availability was quantified by using our survey data to model the probability of site use of frequently taken prey species (Everatt et al., 2015; Searle et al., 2020), thus ensuring an empirical measure of availability that accounts for imperfect detection. For each large carnivore species, the prey species included as covariates were based on prey preferences described in the literature. The probability of illegal human activity was similarly modeled from our survey data and included as an explanatory covariate in the large carnivore models (Everatt et al., 2015, 2019). As for prey, modeling human activity explicitly from survey data rather than through proxies (e.g., distance to boundary and distance to ranger post) allowed us to quantify illegal human activity empirically, ensuring that the covariate was truly representative of anthropogenic disturbances within the PA complex. Information on site covariates for all species at both scales is in Appendix S1.

Covariates were extracted at the sampling unit level: within the 225‐km^2^ cell for the home range scale and within a 250‐m area around each 2‐km segment of survey transect (∼1 km^2^) for the short‐term use scale. Each site was assigned a covariate value equal to the mean of all raster pixel values within that site with the Zonal Statistics tool in QGIS 3.6.3. All covariates were collected at, or representative of, the time of surveying.

### Detection covariates in occupancy models

To account for variation in sampling occasion length following the pooling of segments (see “Occupancy analyses”), the number of 500‐m segments of transect per sampling occasion was modeled as a detection covariate in home‐range‐scale analyses. We also included as a detection covariate an index combining road quality and vehicle use metrics collected during surveying (see “Data collection”) at both scales. This was obtained by summing the scores of the two metrics for each 500‐m segment, and then averaging the scores of each 500‐m segment within a single sampling occasion to obtain a mean value for that sampling occasion.

### Occupancy analyses

We produced single‐season, single‐species occupancy models (Mackenzie et al., 2002) for each prey and large carnivore species and for illegal human activity. Modeling was carried out in package unmarked (Fiske & Chandler, 2011) in R 3.5.3 (R Core Team, 2019) for the home range scale and in program PRESENCE (Hines, 2006) for the short‐term use scale. One of our objectives was to assess the effect of hunting block abandonment on large carnivore occurrence; however, the covariate relating to whether a hunting block was active or vacant is not relevant to sites in photographic areas, potentially resulting in its true effect being masked. We, therefore, also carried out a second home‐range‐scale analysis for each large carnivore species with only data from sites within hunting areas (GRs, GCA, and OA).

We followed a two‐step process to model detection (*p*) and site use (ψ). To identify which of the competing detection models best explained the observed heterogeneity in detection probability based on Akaike information criterion rankings (adjusted for small sample size [AICc] for home‐range‐scale analyses) (Mackenzie et al., 2017), we modeled covariates hypothesized to influence *p* while holding site use constant (with the most parametrized model of noncorrelated covariates). We then modeled ψ by fixing the best‐ranked detection model and varying all possible combinations of noncorrelated site covariates. Covariates were standardized on a *z* scale (MacKenzie et al., 2017) and tested for collinearity with Pearson's correlation test and were not included in the same model if *r* > 0.6 (Green, 1979). To avoid overparameterization, the maximum number of covariates within a single model was informed by the rule of thumb of 15–25 observations (defined here as detections) per predictor variable (Green, 1991).

Models were ranked based on their AIC or AICc scores, and for each analysis, we retained a final model set composed of all models with ΔAIC or ΔAICc of <2, indicating substantial empirical support (Burnham & Anderson, 2004). Maximum‐likelihood site‐specific estimates of *p* and ψ were obtained by model averaging parameters from the final model set based on relative model weights (Burnham & Anderson, 2004). The direction and strength of influence of covariates on site use was determined by their untransformed coefficients (β). Covariates were deemed to have a statistically significant impact if the β coefficient (SE 1.96) did not span zero. We assessed goodness of fit with the MacKenzie and Bailey test for single‐season occupancy models (Mackenzie & Bailey, 2004). Because there is free movement of the target species among sites at both scales, we relaxed the closure assumption of occupancy modeling by interpreting ψ as the probability of site use instead of occupancy (MacKenzie et al., 2017).

Data from track‐based surveys carry an inherent risk of spatial autocorrelation (Hines et al., 2010). For the home‐range‐scale analyses, the use of occupancy models that explicitly account for spatial dependence in detections was precluded by some sampling sites containing independent (noncontinuous) transects. As a result, we employed the methods of Henschel et al. (2016) and Searle et al. (2020) to identify the minimum sampling occasion length required to avoid spatial autocorrelation for each species in PRESENCE (Hines, 2006) and employed this as the sampling occasion length. A summary of the results of the spatial autocorrelation tests is in Appendix S2. At the short‐term use scale, sampling occasions consisted of 2–4 continuous 500‐m segments; therefore, we were able to use models that explicitly accounted for correlation in detections (Hines et al., 2010).

## RESULTS

We surveyed a total of 2235 km of transects across eight PAs (Figure 1). A total of 144 sites were surveyed at the home range scale (average effort per site = 15.5 km), equivalent to ∼75% of all sites in the landscape. This resulted in a total of 1484 sites at the short‐term use scale.

There was no evidence of lack of fit or overdispersion for most models. Where there was some evidence of overdispersion ($\hat{c}$ > 1.5), we followed the recommended practice of adjusting standard errors (Mackenzie et al., 2017).

### Prey and illegal human activity site use

Results of prey detection and site use modeling are in Appendix S3. Eland occupancy could not be modeled at either scale due to low detections and high spatial heterogeneity within these.

Greater illegal human activity was associated with proximity to PA boundary and a lack of regular law enforcement, which both had a significant impact (full results of illegal human activity modeling in Appendix S3). Illegal human activity was slightly greater in exclusively trophy hunting areas (GRs) $(\bar{\hat{\psi }}$ = 0.50 [SE 0.07]) than exclusively photographic Ruaha NP $(\bar{\hat{\psi }}$ = 0.37 [0.06]). Illegal human activity was significantly lower in areas with evidence of regular law enforcement (most of the NP and GRs) than in areas without (WMAs, OA, GCA, portions of the NP and GRs) (Figure 3) and was significantly greater in vacant $(\bar{\hat{\psi }}$ = 0.77 [0.07]) than in actively managed $(\bar{\hat{\psi }}$ = 0.34 [0.07]) hunting blocks (Figure 3).

### Large carnivore site use

Full large carnivore detection and site use model rankings are in Appendix S4.

**FIGURE 2:**
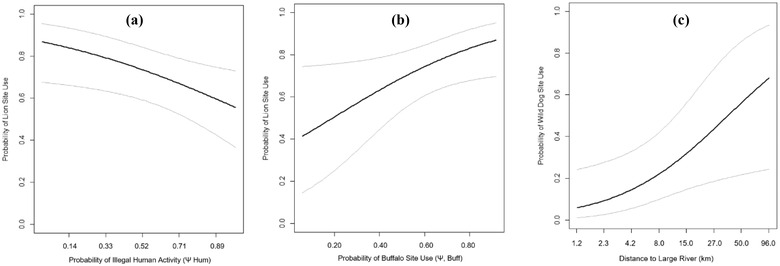
Three important biotic and anthropogenic effects identified in Ruaha‐Rungwa (black line, mean; gray lines, 95% confidence intervals) from the home‐range‐scale analysis across all protected areas (PAs): (a) relationship between the probability of illegal human activity and probability of lion site use ($\bar{\hat{\psi }}$), (b) relationship between the probability of buffalo occurrence ($\bar{\hat{\psi }}$) and probability of lion site use ($\bar{\hat{\psi }}$), and (c) relationship between distance to large river and probability of wild dog site use ($\bar{\hat{\psi }}$). All plots produced from the top‐ranked model containing the covariate based on Akaike information criterion rankings adjusted for a small sample size.

At the home range scale, across all PAs, lions were best predicted by low illegal human activity (β = 0.63 [SE 0.28]) (Figure 2 & Table 1), regular law enforcement (β = 0.59 [0.26]), and high buffalo site use (β = 0.52 [0.25]) (Figure 2), which all had a significant effect. Lion site use exhibited a nonsignificant positive association with actively managed hunting areas, *Acacia‐Commiphora* habitat, and proximity to a ranger post. The overarching management strategy (i.e., photographic vs. hunting tourism) had no meaningful association with lion occurrence. Within hunting areas, lions exhibited a significant association only with actively managed hunting areas (β = 1.16 [0.44]). At the scale of short‐term use within their home range, lions were significantly associated with areas of high buffalo site use (β = 0.25 [0.07]) and proximity to riparian habitat (β = 0.18 [0.07] (Table 1) and nonsignificantly associated with areas of high impala and greater kudu availability.

**TABLE 1:** Summary of β‐coefficient estimates (SE) and relative summed model weights (Σw) of covariates explaining large carnivore site use (ψ) in Ruaha‐Rungwa at two spatial scales.^a^

Species	Scale	Covariate^b^	β (SE)	Significant^c^	Relationship	Σ*w*
Lion						
	home range					
	all protected areas (PAs)					
		probability of illegal human activity	0.63 (0.28)	*	–	0.58
		regular law enforcement	0.59 (0.26)	*	+	0.37
		buffalo site use	0.52 (0.25)	*	+	0.08
		active management in hunting areas	0.69 (0.37)		+	0.91
		primary vegetation type^d^	0.49 (0.28)		–	0.63
		distance to ranger post	0.32 (0.27)		–	0.33
	hunting areas					
		active management in hunting areas	1.16 (0.44)	*	+	1.00
		primary vegetation type^d^	0.54 (0.37)		–	0.85
	short‐term use					
		buffalo site use	0.25 (0.07)	*	+	1.00
		distance to riparian habitat	0.18 (0.07)	*	–	1.00
		impala site use	0.12 (0.06)		+	0.48
		greater kudu site use	0.08 (0.06)		+	0.41
		roan and sable site use	0.10 (0.06)		–	0.27
Leopard						
	home range					
	all PAs					
		regular law enforcement	15.82 (16.97)		+	1.00
		active management in hunting areas	2.27 (1.63)		+	1.00
		distance to riparian habitat	0.43 (0.45)		+	0.20
	hunting areas					
		active management in hunting areas	6.24 (12.60)		+	0.81
		distance to ranger post	1.95 (1.11)		+	0.46
		probability of illegal human activity	13.97 (9.98)		–	0.42
		impala site use	1.36 (0.87)		+	0.24
	short‐term use					
		distance to PA boundary (ln)	0.14 (0.05)	*	+	1.00
		forest and bush cover	0.10 (0.05)	*	+	0.61
		greater kudu site use	0.08 (0.05)		+	0.44
		all prey site use (mean)	0.09 (0.06)		+	0.26
Cheetah						
	home range					
	all PAs					
		probability of illegal human activity	0.60 (0.62)		–	0.14
		regular law enforcement	0.52 (0.49)		+	0.12
	short‐term use					
		distance to riparian habitat	0.52 (0.48)		+	0.16
		distance to PA boundary (ln)	0.27 (0.35)		–	0.09
African wild dog						
	home range					
	all PAs					
		all prey site use (mean)	1.76 (0.69)	*	+	1.00
		distance to large river	1.00 (0.43)	*	+	1.00
	hunting areas					
		all prey site use (mean)	3.28 (1.20)	*	+	1.00
		distance to large river	0.85 (0.50)		+	0.48
		primary vegetation type^d^	0.69 (0.49)		+	0.29
	short‐term use					
		greater kudu site use	0.51 (0.21)	*	+	1.00
		impala site use	0.49 (0.22)	*	–	0.64
		distance to PA boundary (ln)	0.55 (0.25)	*	+	0.60
		forest and bush cover	0.59 (0.25)	*	+	0.36
Spotted hyaena						
	short‐term use					
		buffalo site use	0.29 (0.04)	*	+	1.00
		roan and sable site use	0.13 (0.04)	*	–	1.00

^a^ Only models retained in the final confidence set (ΔAICc < 2) were considered.

^b^ Only the covariates with the strongest effects are presented.

^c^ Effect significant if β (1.96 × SE) does not span zero.

^d^ Symbols: +, miombo woodland; –, *Acacia‐Commiphora* grasslands and bushlands.

* = effect is significant.

At the home range scale, across all PAs, leopard site use did not exhibit significant relationships with any of the covariates. The species nevertheless exhibited nonsignificant positive associations with regular law enforcement, actively managed hunting areas, and greater availability of riparian habitat. Neither overarching management strategy (photographic or hunting tourism) nor illegal human activity affected leopard occurrence at this scale. No significant effects were observed when also restricting the analysis to hunting areas, although active block management was the covariate with the greatest summed model weight (Table 1). For both analyses, the large covariate β coefficients suggested that the species’ high naïve occupancy was likely confounding results to an extent. Within their home range, leopard site use was best predicted by increasing distance to PA boundary (β = 0.14 [SE 0.05]) and more cover (β = 0.10 [0.05]), both of which had a significant effect. Leopards were also nonsignificantly positively associated with areas of the home range with higher greater kudu and mean ungulate prey availability.

At both scales, there was little evidence of any covariate having a strong impact on cheetahs; the null model received strong support. It was not possible to model cheetah site use within hunting areas due to the low number of detections (5) and resulting low naïve occupancy (0.06).

At the home range scale, across all PAs, wild dog site use was best predicted by high availability of prey (β = 1.76 [SE 0.69]) (Table 1) and greater distance to large rivers (β = 1.00 [0.43]) (Figure 2). Similar effects were observed within hunting areas, alongside a nonsignificant positive association with miombo woodlands. Whether an area was used for photographic or hunting tourism appeared to not affect wild dog occurrence. Short‐term wild dog site use was significantly positively associated with greater kudu (β = 0.51 [0.21]) and impala (β = 0.49 [0.22]) site use and with relatively greater distance to PA boundary (β = 0.55 [0.25]) and cover (β = 0.59 [0.25]).

Because spotted hyaenas were detected at all sampled sites at the home range scale, it was not possible to model habitat use at this scale. This suggests that, at this spatial scale, spotted hyaenas were using virtually all areas within PAs in the landscape. At the short‐term use scale, spotted hyaena exhibited a significant positive relationship with buffalo site use (β = 0.29 [SE 0.04]) (Table 1) and a significant negative association with roan and sable site use (β = 0.13 [0.04]). Spotted hyaenas were also nonsignificantly positively associated with areas of the home range close to riparian habitat.

### Mean detection and site use probabilities

**FIGURE 3:**
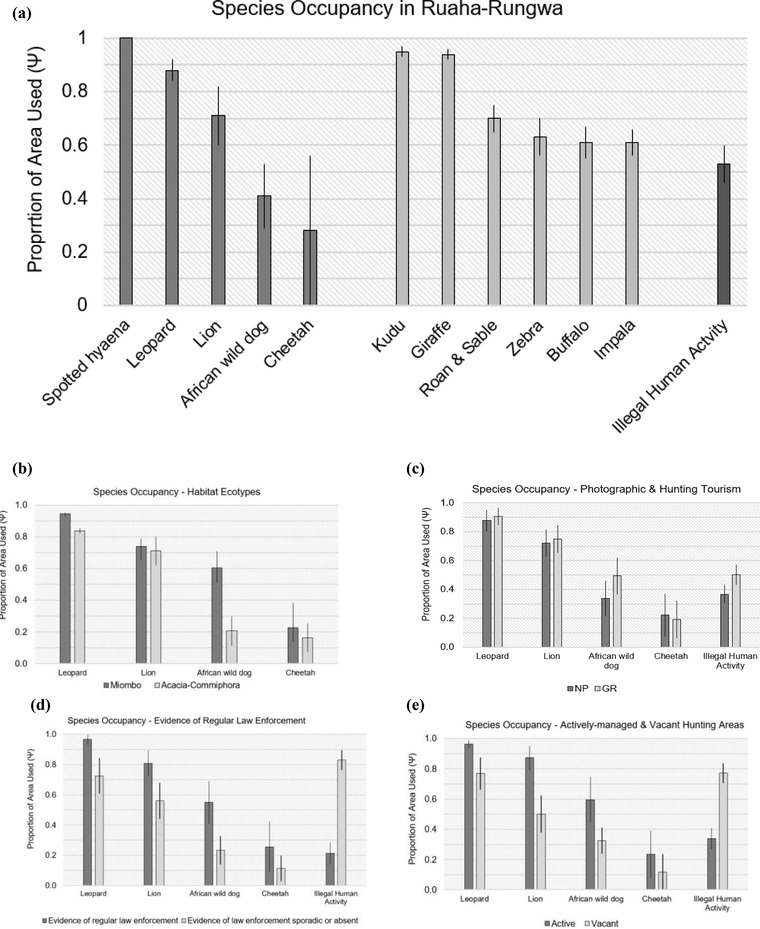
Estimated mean occupancy ($\bar{\hat{\psi }}$), with associated mean of the standard errors, for sites (a) across the Ruaha‐Rungwa landscape; (b) in miombo and *Acacia‐Commiphora* habitats; (c) in exclusively photographic (NP) and trophy hunting (GR) tourism areas; (d) in areas with and without evidence of regular, sustained law enforcement; and (e) in actively managed and vacant blocks in areas reserved exclusively for trophy hunting (GRs). Conditional occupancy was used to plot spotted hyaena site use in (a), whereas the species was not included in (b–d) due to its ubiquity across the landscape.

Mean site use was significantly greater in miombo woodlands than in *Acacia‐Commiphora* for wild dogs, and similar for all other large carnivores (Table 2 & Figure 3). Similarly, mean wild dog site use was greater in exclusively trophy hunting areas (GRs) $(\bar{\hat{\psi }}$ = 0.49 [SE 0.13]) than exclusively photographic Ruaha NP $(\bar{\hat{\psi }}$ = 0.34 [0.12]), but similar for other large carnivores. For all large carnivores, mean site use was greater in areas with evidence of regular and sustained law enforcement than in areas without (Figure 3). Mean site use in actively managed hunting areas was 0.87 (0.08) for lions and 0.96 (0.03) for leopards, whereas it was 0.50 (0.12) for lions and 0.77 (0.11) for leopards in vacant hunting areas. A similar relationship was observed in nonhunted species (wild dog: $\bar{\hat{\psi }}$ = 0.60 [0.15] vs. $\bar{\hat{\psi }}$ = 0.32 [0.09]; cheetah: $\bar{\hat{\psi }}$ = 0.23 [0.16] vs. $\bar{\hat{\psi }}$ = 0.12 [0.12]) (Figure 3).

**TABLE 2:** Number of independent detections, naïve occupancy (ψ), and model‐averaged mean probability of detection ($\bar{\hat{p}}$) and site use ($\bar{\hat{\psi }}$)^a^ for prey, illegal human activity, and large carnivores at the home range scale across all protected areas

Species	Independent detections	Naïve ψ	$\bar{\hat{p}}$ (SE)	$\bar{\hat{\psi }}$ (SE)
Buffalo	187	0.45	0.44 (0.05)	0.61 (0.06)
Zebra	212	0.47	0.54 (0.07)	0.63 (0.07)
Giraffe	460	0.77	0.75 (0.02)	0.94 (0.02)
Roan and sable	336	0.58	0.77 (0.03)	0.70 (0.05)
Greater kudu	545	0.85	0.84 (0.03)	0.95 (0.02)
Impala	283	0.54	0.64 (0.04)	0.61 (0.05)
Illegal human activity	164	0.50	0.51 (0.03)	0.53 (0.07)
Lion	159	0.57	0.49 (0.19)	0.70 (0.11)
Leopard	233	0.82	0.51 (0.13)	0.88 (0.04)
Cheetah	11	0.07	0.07 (0.06)	0.28 (0.28)
African wild dog	40	0.22	0.19 (0.08)	0.41 (0.12)
Spotted hyaena^b^	432	1.00	–	–

^a^ Also interpretable as the proportion of sampled area used by the species.

^b^ Spotted hyaena site use could not be modeled as the species was detected at all sites sampled.

## DISCUSSION

### Large carnivore occurrence and habitat use

The most regionally threatened large carnivore species, cheetahs and wild dogs (Wolf & Ripple, 2017), were also the least widely distributed in Ruaha‐Rungwa (Table 2 & Figure 3). As expected, spotted hyaenas and leopards––species which have shown environmental flexibility and resilience to anthropogenic pressures (Henschel et al., 2020; Petracca et al., 2019)––were relatively widespread and able to persist in more affected areas. Although still fairly well distributed, lions were limited across the landscape, and there was evidence of top‐down limitation.

### Human impacts in PAs

The best predictors of home range scale (second order) use by lions were low illegal human activity and regular law enforcement (Table 1). Lion site use declined as illegal human activity increased (Figure 2); this suggests that the species is experiencing top‐down pressures even within PAs. The observed effect was likely a result of both direct mortality (e.g., through snaring [Everatt et al., 2015]) and indirect effects (e.g., prey base depletion and inability to persist in depleted areas [Bauer et al., 2020]). Our findings thus add to the growing body of evidence that this species is particularly vulnerable to human disturbances within PAs (Farr et al., 2017; Petracca et al., 2019). As the dominant competitor within their guild, lions are unlikely to have evolved appropriate strategies for mitigating competition with other species and may, therefore, be less able to adapt to competition from humans (Everatt et al., 2019). Our findings thus highlight the important role of active protection for the species (Henschel et al., 2016) and align with others in suggesting that anthropogenic rather than ecological factors have the strongest impacts on lion habitat use in disturbed areas (Petracca et al., 2019).

### Effects of biotic resources

In contrast, wild dogs were limited by biotic rather than anthropogenic factors. At the home range scale, the species was strongly associated with areas farther from large rivers (Figure 2), but that nonetheless exhibited relatively high levels of prey availability. Although areas near large rivers host the highest prey concentrations in the dry season (TAWIRI, 2019), they also exhibit the highest densities of dominant sympatric predators, including lions (Strampelli et al., 2022). Wild dogs can be suppressed in areas of high lion and spotted hyaena density (Creel & Creel, 1998); by indicating avoidance of (or inability to persist in) these high‐prey but also high‐competitor areas, our results provide further evidence for such effects. Instead, wild dogs appeared to be associated with areas that still had relatively high prey availability, but had lower densities of sympatric predators. Mean wild dog site use was significantly greater in miombo woodlands (Figure 3), where sympatric competitor densities are lower (Searle et al., 2021; Strampelli et al., 2022). This is likely to also be a result of competitor avoidance or of greater survival as a result of kleptoparasitism and direct mortality being minimized in woodlands (Creel & Creel, 1998; Mills & Gorman, 1997).

These observed dual requirements––low interspecific pressure and high prey availability––suggest that wild dog persistence is particularly fragile. These requirements may also have contributed to the drastic population and range decreases experienced by the species (Wolf & Ripple, 2017). In contrast, our finding that wild dogs were significantly associated with riparian areas within their home range suggests that, by avoiding dominant competitors at a coarser scale, wild dogs may not be forced to avoid such areas at finer scales. Hierarchical avoidance strategies of this kind have been noted elsewhere (e.g., Polfus et al., 2011) and should be given appropriate consideration in conservation planning.

Although lions were primarily limited by top‐down pressures, our results also revealed some important biotic relationships for the species. As found elsewhere (Everatt et al., 2015), buffalo presence was strongly associated with lion occurrence at the home‐range scale (Figure 2) and was a better predictor than any other prey species (including mean prey availability). In contrast, lions appeared to make short‐term use decisions based on both the availability and vulnerability of prey. Unlike at the coarser scale, significant associations were observed between lions and areas of their home range exhibiting both greater prey availability (especially buffalo) and greater prey catchability, in the form of a significant positive association with riparian habitats, where prey are more susceptible to predation (Hopcraft et al., 2005).

For leopards, the lack of significant covariate effects at the home range scale confirmed that, as long as some prey are available, the species can persist across a range of habitats (Estes, 1991; Strampelli et al., 2018). In contrast, finer scale habitat use by leopard was significantly associated with availability of cover, as noted by Miller et al. (2018). These findings highlight the importance of accounting for multiple scales in habitat use studies (Everatt et al., 2015). For cheetahs, the lack of significant covariate associations suggests either relatively homogeneous habitat use or insufficient data to model this effectively. Given the low number of detections, the latter appears most likely. Finally, the ubiquity of spotted hyaenas within PAs (Figure 3) highlights the species’ adaptability to a range of environmental conditions, and its resilience to anthropogenic disturbance and prey‐base depletion.

### Ruaha‐Rungwa's large carnivore guild and the importance of heterogeneous landscapes

Our study reveals that Ruaha‐Rungwa hosts globally important populations of lion, leopard, wild dog, spotted hyaena, and possibly cheetah and striped hyaena (but more in‐depth assessments are required for the latter two species). Nevertheless, illegal human activities within PAs were found to be an important threat to these populations, even within better‐protected PAs. Efforts to reduce such disturbances, particularly where they are greatest (vacant hunting blocks, southwestern Ruaha NP, GCA, OA, and WMAs) (distribution maps from the occupancy modeling, including of prey and illegal human activity, are in Appendix S5), would thus have a beneficial impact on the complex's large carnivores, particularly lion. Furthermore, large carnivores were widespread in boundary areas, where mortalities from accidental snaring and direct persecution are common (Dickman et al., 2014). Such areas are likely to be acting as population sinks, and conflict‐mitigation activities and other initiatives that reduce large carnivore mortalities (e.g., by increasing tolerance by tying wildlife presence to financial benefits [RCP, 2019]) should be considered priorities for the landscape.

Our results also have implications for conservation planning elsewhere in Africa. While some species (e.g., lion) thrived in more productive riparian or grassland areas, drier or less‐productive habitats, such as miombo woodlands, were critical for wild dogs and a number of ungulate species. Conservation managers and policy makers should be aware of this heterogeneity in habitat requirements, and protection should be provided to a breadth of complementary habitat types and biotic features, rather than solely to core areas of higher wildlife densities (Watson et al., 2011). Similarly, the different priorities identified for our study species suggest that informed, holistic management decisions would be precluded by single‐species efforts, as indicated by similar studies elsewhere (e.g., Petracca et al., 2019). As such, our findings highlight the importance of adopting an integrated, multispecies approach to large carnivore conservation research and support the need for landscape‐scale conservation planning in African PA systems (Trombulak & Baldwin, 2010).

### Competing management strategies and the growing threat of hunting area abandonment

Whether an area was designated for hunting or photographic tourism did not affect large carnivore occurrence. This result mirrors Mills et al.’s (2020) finding of comparable occurrence of lions in hunting concessions and NPs in West Africa. Nonetheless, we recommend further research into whether hunting offtake is eliciting finer‐scale effects on the study populations. For example, while hunting did not affect leopard site use in Zimbabwe, it negatively affected abundance (Searle et al., 2020), and similar effects have been noted for lions (Creel et al., 2016). Our results nevertheless suggest that large carnivores can persist in hunting areas if these are effectively managed, and that management and protection levels are a better determinant of persistence than whether the area is used for photographic or hunting tourism (as also noted by Bauer et al. [2015]).

Further evidence of this was provided by our finding that large carnivores were faring better in areas with evidence of regular and sustained law enforcement activities than areas without (WMAs, GCA, OA, sections of the NP and GR), and in actively managed hunting areas compared with those left vacant by operators (Figure 3). In both cases, we believe this is primarily a result of differences in management resources. In Tanzania, NPs and GRs generally receive greater conservation investment than WMAs, GCAs, and OAs (Stoner et al., 2007; this study), and the higher illegal human activity and lower wildlife occurrence observed in the latter confirm patterns noted elsewhere (Oberosler et al., 2019; Stoner et al., 2007). Similarly, hunting operators in Tanzania are required to support regular protection activities within their blocks (MNRT, 2018). When blocks are vacated, management is returned to the Tanzania Wildlife Management Authority (TAWA), which often lacks the resources to effectively protect such vast areas, particularly without the income generated from hunting activities (EU, 2017; MNRT, 2016; TAWA, personal communication). Our findings thus suggest that increased protection associated with the presence of hunting operators can improve the management of wildlife habitats in the absence of other sources of conservation funding. Nevertheless, we were unable to determine whether poor management by hunting operators played a role in blocks becoming degraded before they were abandoned. This possibility should not be excluded, given past evidence of overharvesting (Brink et al., 2016; Packer et al., 2011). Ensuring sustainable practices, and that operators implement regular protection activities, should be a priority for PA managers.

Regardless of underlying drivers, block vacancy was the best predictor of lion and leopard absence within hunting areas, indicating that the high level of human disturbance in vacant hunting blocks is a key conservation threat in the study landscape. From 2014 to 2018, approximately half of Tanzania's hunting areas, covering almost 150,000 km^2^, were vacated by hunting operators (FZS, 2018). Thus, if the situation in Ruaha‐Rungwa is indicative of vacant blocks elsewhere in the country, the dereliction of protective management associated with hunting block vacancy may present a novel and important threat to Tanzania's biodiversity. This is likely to be the case especially where human population density around PA boundaries is high, as in parts of the study area. We, therefore, encourage further research into this emerging threat, both in Tanzania and in other countries experiencing similar trends of hunting area abandonment (Zambia and Mozambique; FZS, 2018).

In light of these findings, we also caution against attempts to phase out trophy hunting (or, more specifically, protection activities associated with it) without appropriate alternatives being in place (Dickman et al., 2019; Lindsey et al., 2007). Given the ongoing conservation funding shortage (Lindsey et al., 2018) and the size of the areas of concern, both consumptive and nonconsumptive strategies are likely to be required to preserve Africa's vast network of wildlife areas (Dickman et al., 2019; Lindsey et al., 2007). These will likely have to involve mechanisms that are both established (photographic and hunting tourism) and innovative (e.g., debt for nature swaps, carbon payments, conservation basic incomes, private philanthropy, joint ventures with the private or nongovernmental organization sectors, and sustainable livestock‐wildlife systems [Buscher & Fletcher, 2020; Lindsey et al., 2014]). We, therefore, urgently recommend investments to identify and implement solutions to what appears to be a novel and potentially severe threat to the region's wildlife and its habitats.

## Supporting information

Appendix information
