# Supplementary material for: Habitat use of and threats to African large carnivores in a mixed‐use landscape
Source: Conserv Biol. 2022 Aug 4;36(6):e13943. doi: 10.1111/cobi.13943 (PMC10087927; doi:10.1111/cobi.13943)
Supplement: Supplementary file 1 — Appendix information [file COBI-36-0-s001.docx]

Appendix S1 – Site Use Covariates

| **Table S1.1** Covariates hypothesised to influence site use by prey (ungulate) species, at both spatial scales. All covariates were tested at both spatial scales investigated. | | | | | |
| --- | --- | --- | --- | --- | --- |
| **Covariate** | **Handle** | **Relationship to ungulate occurrence** | **Measurement (site level)** | **Data source** | **Hypothesised direction of effect** |
| **Biotic** | | | | | |
| Water | W | Dry season surface water availability | Mean probability of water availability (mean pixel value) | Google Earth (hand digitised) | - (Sable/roan)  + (All others) |
| NDVI (mean) | NDVIave | Nutritional resources (primary productivity) | Mean NDVI value at site during survey period (mean pixel value) | MCD43A4_NDVI (NASA LP DAAC, https://lpdaac.usgs. gov/) | + (All) |
| NDVI (standard deviation) | NDVIstd | Nutritional resources (primary productivity) | Standard deviation of NDVI values at site during survey period (mean pixel value) | MCD43A4_NDVI (NASA LP DAAC, https://lpdaac.usgs. gov/) | + (All) |
| Tree cover | C | Nutritional resources (primary productivity) | % cover (woodland only) | ESA CCI Land Cover – S2 prototype land cover 20m map of Africa  <http://2016africalandcover20m.esrin.esa.int/> | + (All) |
| Primary habitat ecotype | H | Predominant vegetation type (*miombo* or *Acacia-commiphora*) | Habitat ecotype (miombo or *Acacia-commiphora*) - % site miombo | WWF Terrestrial Ecoregions of the World (<https://www.worldwildlife.org/publications/terrestrial-ecoregions-of-the-world>) | + (Sable/roan)  - (Impala) |
| **Abiotic – Anthropogenic Impacts** | | | | | |
| Habitat conversion to agriculture^1^ | Crop | Unsuitable habitat | % converted habitat in cell | Google Earth Grids (Jacobsen *et al*., 2015) | - (All) |
| Distance to Human Settlements^1^ | DistV | Direct persecution, competition with livestock | Natural logarithm of mean distance to nearest settlement, with asymptote (Log) | Settlements & hamlets GIS layer (hand digitised) | - (All) |
| **Abiotic – Management** | | | | | |
| Legal protection^1^ | PA | Protection from targeted or accidental mortalities | % site legally protected | IUCN WDPA (WDPA; <https://protectedplanet.net/>), Riggio *et al*. (2017) | + (All) |
| Regular law Enforcement | Enf | Protection from targeted or accidental mortalities, prey base protection | % site with evidence of regular, sustained anti-poaching ^2^ | This study | + (All) |
| ^1^ Site use of prey species was estimated as part of a broader study which included non-protected areas (village lands); thus, effects of habitat conversion, distance to human settlements, and legal protection were also tested  ^2^ Includes regular protection activities by management authorities (TAWA, TANAPA) or others (e.g. NGOs, hunting operators). Assessed through data collected during surveys, and cross-referenced with management authorities (TAWA, TANAPA) | | | | | |

| **Table S1.2** Covariates hypothesised to influence illegal human activity site use, at both spatial scales. All covariates were tested at both spatial scales investigated. | | | | | |
| --- | --- | --- | --- | --- | --- |
| **Covariate** | **Handle** | **Relationship to illegal human use** | **Measurement (site level)** | **Data source** | **Hypothesised direction of effect** |
| **Biotic** | | | | | |
| Water | W | Dry season surface water availability | Mean probability of water availability (mean pixel value) | Hand digitised | + |
| All prey | AllPrey | Availability of wild ungulates | Mean probability of site use of six prey species (buffalo, zebra, roan & sable, impala, kudu, giraffe) | This study | + |
| Tree cover | C | Increased attractiveness for logging | % cover (woodland only) | ESA CCI Land Cover – S2 prototype land cover 20m map of Africa  <http://2016africalandcover20m.esrin.esa.int/> | + |
| **Other** | | | | | |
| Distance to PA boundary | Bound | Increased difficulty of access | Mean distance to nearest PA boundary | IUCN World Database on Protected Areas, modified (WDPA; <https://protectedplanet.net/>), Riggio *et al*. (2017) | - |
| Distance to ranger post | Post | Protection from targeted or accidental mortalities, prey base protection | Distance to nearest range post, with asymptote (Ln) | This study | + |
| Regular law Enforcement | Enf | Avoidance of better protected areas | % site with evidence of regular, sustained anti-poaching ^1^ | This study | - |
| ^1^ Includes regular protection activities by management authorities (TAWA, TANAPA) or others (e.g. NGOs, hunting operators). Assessed through data collected during surveys, and cross-referenced with management authorities (TAWA, TANAPA) | | | | | |

| **Table S1.3** Covariates hypothesised to influence lion site use at the home range and short-term use spatial scales | | | | | | | |
| --- | --- | --- | --- | --- | --- | --- | --- |
| **Covariate** | **Handle** | **Relationship to occurrence** | **Measurement (site level)** | **Data source** | **Published evidence of effect** | **Spatial Scale** | **Hypothesised effect** |
| **Biotic – Prey** | | | | | | | |
| Preferred prey (buffalo) site use | Buff | Availability of preferred prey / buffalo | Probability of site use of buffalo | This study | Everatt et al., 2015; Hayward & Kerley, 2005 | Both | + |
| Alternate prey site use | SecPrey | Availability of alternate prey | Mean probability of site use of other prey species (zebra, giraffe, roan & sable, kudu, impala) | This study | Everatt et al., 2015; Hayward & Kerley, 2005 | Both | + |
| Mean prey site use | AllPrey | Availability of all prey | Mean probability of site use of all prey species (buffalo, zebra, roan & sable, impala, kudu, giraffe) | This study | Hayward & Kerley, 2005 | Both | + |
| **Biotic – Other** | | | | | | | |
| Cover | C | Habitat features facilitating prey capture | % cover (woodland or bushland) | ESA CCI Land Cover – S2 prototype land cover 20m map of Africa  <http://2016africalandcover20m.esrin.esa.int/> | Hopcraft et al., 2005 | Both | - |
| Riparian habitat | R | Habitat features facilitating prey capture | Mean Euclidian distance to nearest riparian habitat | Rivers & drainage lines GIS layer (hand digitised) | Hopcraft et al., 2005 | Both | + |
| Primary habitat ecotype | H | Habitat features facilitating prey capture, nutritional variation | Habitat ecotype (miombo or *Acacia-commiphora*) - % site miombo | WWF Terrestrial Ecoregions of the World (<https://www.worldwildlife.org/publications/terrestrial-ecoregions-of-the-world>) | Midlane et al., 2014 | Home Range^1^ | / |
| **Abiotic – Anthropogenic Impacts** | | | | | | | |
| Distance to PA boundary | Bound | Persecution, unsuitable habitat | Distance to nearest settlement, with asymptote (Ln) | IUCN WDPA, modified (WDPA; <https://protectedplanet.net/>), Riggio *et al*. (2017) | Bauer et al., 2016 | Both | + |
| Illegal human use | Hum | Targeted or accidental snaring, behavioural avoidance | Probability of site use of illegal human activity | This study | Everatt et al., 2015 | Both | - |
| **Abiotic – Management** | | | | | | | |
| Regular law Enforcement | Enf | Protection from targeted or accidental mortalities, prey base protection | % site with evidence of regular, sustained anti-poaching ^2^ | This study | Henschel et al., 2016; Packer et al., 2013 | Home range^1^ | + |
| Distance to ranger post | Post | Protection from targeted or accidental mortalities, prey base protection | Distance to nearest range post, with asymptote (Ln) | This study | Henschel et al., 2016; Packer et al., 2013 | Both | - |
| Trophy hunting (permitted) | Hnt | Offtake mortalities | % site where hunting is permitted | Tanzania Wildlife Authority (TAWA) | Croes et al., 2011; Packer et al., 2011 | Home Range^1^ | - |
| Trophy hunting (occurring) | Mgd | Offtake mortalities | % site in actively managed hunting area | Tanzania Wildlife Authority (TAWA) | Croes et al., 2011; Packer et al., 2011 | Home Range^1^ | - |
| ^1^ Covariate not included at the short-term use within the home range scale as it exhibited little or no heterogeneity within most individual home range scale sites, and is therefore not relevant to habitat use decisions at this scale  ^2^ Includes regular protection activities by management authorities (TAWA, TANAPA) or others (e.g. NGOs, hunting operators). Assessed through data collected during surveys, and cross-referenced with management authorities (TAWA, TANAPA) | | | | | | | |

| **Table S1.4** Covariates hypothesised to influence leopard site use at the home range and short-term use spatial scales | | | | | | | | |
| --- | --- | --- | --- | --- | --- | --- | --- | --- |
| **Covariate** | **Handle** | **Relationship to occurrence** | **Measurement (site level)** | **Data source** | **Published evidence of effect** | | **Spatial Scale** | **Hypothesised effect** |
| **Biotic – Prey** | | | | | | | | |
| Preferred prey (impala) | Imp | Availability of preferred prey / impala | Probability of site use of impala | This study | Hayward et al., 2006 | Both | | + |
| All prey | AllPrey | Availability of all prey | Mean probability of site use of all prey species (buffalo, zebra, roan & sable, impala, kudu, giraffe) | This study | Hayward et al., 2006 | Both | | + |
| **Biotic – Other** | | | | | | | | |
| Cover | C | Habitat features facilitating prey capture | % cover (woodland or bushland) | ESA CCI Land Cover – S2 prototype land cover 20m map of Africa  <http://2016africalandcover20m.esrin.esa.int/> | Balme et al., 2007; Henschel et al., 2020 | Both | | + |
| Riparian habitat | R | Habitat features facilitating prey capture | Mean Euclidian distance to nearest riparian habitat | Rivers & drainage lines GIS layer (hand digitised) | Burton et al., 2012 | Both | | + |
| Primary habitat ecotype | H | Habitat features facilitating prey capture, nutritional variation | Habitat ecotype (miombo or *Acacia-commiphora*) - % site miombo | WWF Terrestrial Ecoregions of the World (<https://www.worldwildlife.org/publications/terrestrial-ecoregions-of-the-world>) | Kane et al., 2014 | Home Range^1^ | | / |
| **Abiotic – Anthropogenic impacts** | | | | | | | | |
| Distance to PA boundary | Bound | Persecution, unsuitable habitat | Distance to nearest settlement, with asymptote (Ln) | IUCN World Database on Protected Areas, modified (WDPA; <https://protectedplanet.net/>), Riggio *et al*. (2017) | Stein et al., 2016 | Both | | + |
| Illegal human use | Hum | Targeted or accidental snaring, behavioural avoidance | Probability of site use of illegal human activity | This study | Henschel et al., 2011 | Both | | - |
| **Abiotic – Management** | | | | | | | | |
| Regular law Enforcement | Enf | Protection from targeted or accidental mortalities, prey base protection | % site with evidence of regular, sustained anti-poaching ^1^ | This study | Madsen & Broekhuis, 2018 | Home Range^1^ | | + |
| Distance to ranger post | Post | Protection from targeted or accidental mortalities, prey base protection | Distance to nearest range post, with asymptote (Ln) | This study | Madsen & Broekhuis, 2018 | Both | | - |
| Trophy hunting (permitted) | Hnt | Offtake mortalities | % site where hunting is permitted | Tanzania Wildlife Authority (TAWA) *pers comm*.; this study | Packer et al., 2011 | Home Range^1^ | | - |
| Trophy hunting (occurring) | Mgd | Offtake mortalities | % site in actively managed hunting area | Tanzania Wildlife Authority (TAWA) *pers comm*.; this study | Packer et al., 2011 | Home Range^1^ | | - |
| ^1^ Covariate not included at the short-term use within the home range scale as it exhibited little or no heterogeneity within most individual home range scale sites, and is therefore not relevant to habitat use decisions at this scale  ^2^ Includes regular protection activities by management authorities (TAWA, TANAPA) or others (e.g. NGOs, hunting operators). Assessed through data collected during surveys, and cross-referenced with management authorities (TAWA, TANAPA) | | | | | | | | |

| **Table S1.5** Covariates hypothesised to influence cheetah site use at the home range and short-term use spatial scales | | | | | | | |
| --- | --- | --- | --- | --- | --- | --- | --- |
| **Covariate** | **Handle** | **Relationship to cheetah occurrence** | **Measurement (site level)** | **Data source** | **Published evidence of effect** | **Spatial Scale** | **Hypothesised effect** |
| **Biotic – Prey** | | | | | | | |
| Preferred prey (impala) | Imp | Availability of preferred prey / impala | Probability of site use of impala^2^ | This study | Andresen et al., 2014; Hayward, Hofmeyr, et al., 2006 | Both | + |
| All prey | AllPrey | Availability of all prey | Mean probability of site use of all prey species (buffalo, zebra, roan & sable, impala, kudu, giraffe)^2^ | This study | Hayward, Hofmeyr, et al., 2006 | Both | + |
| **Biotic – Other** | | | | | | | |
| Cover | C | Habitat features facilitating prey capture | % cover (woodland or bushland) | ESA CCI Land Cover – S2 prototype land cover 20m map of Africa  <http://2016africalandcover20m.esrin.esa.int/> | Bissett & Bernard, 2007; Pettorelli et al., 2009 | Both | + |
| Riparian habitat | R | Habitat features facilitating prey capture | Mean Euclidian distance to nearest riparian habitat | Rivers & drainage lines GIS layer (hand digitised) | Broomhall et al., 2003; M. G. L. Mills et al., 2004 | Both | - |
| Primary habitat ecotype | H | Habitat features facilitating prey capture, nutritional variation | Habitat ecotype (miombo or *Acacia-commiphora*) - % site miombo | WWF Terrestrial Ecoregions of the World (<https://www.worldwildlife.org/publications/terrestrial-ecoregions-of-the-world>) | Broomhall et al., 2003; M. G. L. Mills et al., 2004 | Home Range^1^ | / |
| Distance to major river | Riv | Avoidance of areas of high densities of dominant competitors | Mean Euclidian distance to nearest large river | Rivers & drainage lines GIS layer (hand digitised) | Durant, 1998; Pettorelli et al., 2009 | Both | + |
| **Abiotic – Anthropogenic impacts** | | | | | | | |
| Distance to PA boundary | Bound | Persecution, unsuitable habitat | Distance to nearest settlement, with asymptote (Ln) | IUCN World Database on Protected Areas, modified (WDPA; <https://protectedplanet.net/>), Riggio *et al*. (2017) | Klaassen & Broekhuis, 2018; Madsen & Broekhuis, 2018 | Both | + |
| Illegal human use | Hum | Targeted or accidental snaring, behavioural avoidance | Probability of site use of illegal human activity^2^ | This study | Klaassen & Broekhuis, 2018; Madsen & Broekhuis, 2018 | Both | - |
| **Abiotic – Management** | | | | | | | |
| Regular law Enforcement | Enf | Protection from targeted or accidental mortalities, prey base protection | % site with evidence of regular, sustained anti-poaching ^1^ | This study | Klaassen & Broekhuis, 2018; Madsen & Broekhuis, 2018 | Home Range^1^ | + |
| Distance to ranger post | Post | Protection from targeted or accidental mortalities, prey base protection | Distance to nearest range post, with asymptote (Ln) | This study | Klaassen & Broekhuis, 2018; Madsen & Broekhuis, 2018 | Both | - |
| Trophy hunting (permitted) | Hnt | Disturbance / prey offtake | % site where hunting is permitted | Tanzania Wildlife Authority (TAWA) *pers comm*.; this study | Durant et al., 2015 | Home Range^1^ | - |
| Trophy hunting (occurring) | Mgd | Disturbance / prey offtake | % site in actively managed hunting area | Tanzania Wildlife Authority (TAWA) *pers comm*.; this study | Durant et al., 2015 | Home Range^1^ | - |
| ^1^ Covariate not included at the short-term use within the home range scale as it exhibited little or no heterogeneity within most individual home range scale sites, and is therefore not relevant to habitat use decisions at this scale  ^2^ Includes regular protection activities by management authorities (TAWA, TANAPA) or others (e.g. NGOs, hunting operators). Assessed through data collected during surveys, and cross-referenced with management authorities (TAWA, TANAPA) | | | | | | | |

| **Table S1.6** Covariates hypothesised to influence wild dog site use at the home range and short-term use spatial scales | | | | | | | |
| --- | --- | --- | --- | --- | --- | --- | --- |
| **Covariate** | **Handle** | **Relationship to wild dog occurrence** | **Measurement (site level)** | **Data source** | **Published evidence of effect** | **Spatial Scale** | **Hypothesised effect** |
| **Biotic – Prey** | | | | | | | |
| Preferred prey (impala) | Imp | Availability of preferred prey / impala | Probability of site use of impala | This study | Hayward, O’Brien, et al., 2006 | Both | + |
| Preferred prey (kudu) | Kud | Availability of kudu | Probability of site use of kudu | This study | Hayward, O’Brien, et al., 2006 | Both | + |
| All prey | AllPrey | Availability of all prey | Mean probability of site use of all prey species (buffalo, zebra, roan & sable, impala, kudu, giraffe) | This study | Hayward, O’Brien, et al., 2006 | Both | + |
| **Biotic – Other** | | | | | | | |
| Cover | C | Habitat features facilitating prey capture | % cover (woodland or bushland) | ESA CCI Land Cover – S2 prototype land cover 20m map of Africa  <http://2016africalandcover20m.esrin.esa.int/> | M. G. L. Mills & Gorman, 1997; Shumba et al., 2018 | Both | + |
| Riparian habitat | R | Habitat features facilitating prey capture | Mean Euclidian distance to nearest riparian habitat | Rivers & drainage lines GIS layer (hand digitised) | Creel & Creel, 2002; M. G. L. Mills & Gorman, 1997 | Both | - |
| Habitat type | H | Habitat features facilitating prey capture, nutritional variation | Habitat type (*miombo* or *Acacia-commiphora*) - % site *miombo* | WWF Terrestrial Ecoregions of the World (<https://www.worldwildlife.org/publications/terrestrial-ecoregions-of-the-world>) | M. G. L. Mills & Gorman, 1997; Shumba et al., 2018 | Home Range^1^ | / |
| Distance to major river | Riv | Avoidance of areas of high densities of dominant competitors | Mean Euclidian distance to nearest large river | Rivers & drainage lines GIS layer (hand digitised) | Creel & Creel, 2002; M. G. L. Mills & Gorman, 1997 | Both | + |
| **Abiotic – Anthropogenic impacts** | | | | | | | |
| Distance to PA boundary | Bound | Persecution, unsuitable habitat | Distance to nearest settlement, with asymptote (Ln) | IUCN WDPA (WDPA; <https://protectedplanet.net/>), Riggio *et al*. (2017) | Van Der Meer et al., 2014 | Both | + |
| Illegal human use | Hum | Targeted or accidental snaring, behavioural avoidance | Probability of site use of illegal human activity | This study | Woodroffe & Sillero-Zubiri, 2020 | Both | - |
| **Abiotic – Management** | | | | | | | |
| Regular law Enforcement | Enf | Protection from targeted or accidental mortalities, prey base protection | % site with evidence of regular, sustained anti-poaching ^1^ | This study | Van Der Meer et al., 2014 | Home Range^1^ | + |
| Distance to ranger post | Post | Protection from targeted or accidental mortalities, prey base protection | Distance to nearest range post, with asymptote (Ln) | This study | Van Der Meer et al., 2014 | Both | - |
| Trophy hunting (permitted) | Hnt | Offtake mortalities | % site where hunting is permitted | Tanzania Wildlife Authority (TAWA) *pers comm*.; this study | Woodroffe & Sillero-Zubiri, 2020 | Home Range^1^ | - |
| Trophy hunting (occurring) | Mgd | Offtake mortalities | % site in actively managed hunting area | Tanzania Wildlife Authority (TAWA) *pers comm*.; this study | Woodroffe & Sillero-Zubiri, 2020 | Home Range^1^ | - |
| ^1^ Covariate not included at the short-term use within the home range scale as it exhibited little or no heterogeneity within most individual home range scale sites, and is therefore not relevant to habitat use decisions at this scale  ^2^ Includes regular protection activities by management authorities (TAWA, TANAPA) or others (e.g. NGOs, hunting operators). Assessed through data collected during surveys, and cross-referenced with management authorities (TAWA, TANAPA) | | | | | | | |

| **Table S1.7** Covariates hypothesised to influence spotted hyaena site use at short-term use scale. Spotted hyaena site use could not be modelled at the at the home range scale due to the species being detected at all sites. | | | | | | |
| --- | --- | --- | --- | --- | --- | --- |
| **Covariate** | **Handle** | **Relationship to spotted hyaena occurrence** | **Measurement (site level)** | **Data source** | **Published evidence of effect** | **Hypothesised effect** |
| All prey | AllPrey | Availability of all prey | Mean probability of site use of all prey species (buffalo, zebra, roan & sable, impala, kudu, giraffe) | This study | Hayward, 2006 | + |
| Buffalo | Buff | Availability of buffalo | Probability of site use of buffalo | This study | Hayward, 2006 | + |
| Kudu | Kud | Availability of kudu | Probability of site use of kudu | This study | Hayward, 2006 | + |
| Impala | Imp | Availability of impala | Probability of site use of impala | This study | Hayward, 2006 | + |
| Roan & Sable | Sbl | Availability of roan & sable | Probability of site use of roan & sable | This study | Hayward, 2006 | + |
| Giraffe | Giff | Availability of giraffe | Probability of site use of giraffe | This study | Hayward, 2006 | + |
| Zebra | Zeb | Availability of zebra | Probability of site use of zebra | This study | Hayward, 2006 | + |
| Cover | C | Habitat features facilitating prey capture | % cover (woodland or bushland) | ESA CCI Land Cover – S2 prototype land cover 20m map of Africa  <http://2016africalandcover20m.esrin.esa.int/> | Boydston et al., 2003 | - |
| Riparian habitat | R | Habitat features facilitating prey capture | Mean Euclidian distance to nearest riparian habitat | Rivers & drainage lines GIS layer (hand digitised) | Kolowski & Holekamp, 2009 | + |
| Habitat type | H | Habitat features facilitating prey capture, nutritional variation | Habitat type (*miombo* or *Acacia-commiphora*) - % site *miombo* | WWF Terrestrial Ecoregions of the World (<https://www.worldwildlife.org/publications/terrestrial-ecoregions-of-the-world>) | Pettorelli et al., 2010 | / |
| Distance to PA boundary | Bound | Persecution, unsuitable habitat | Distance to nearest settlement, with asymptote (Ln) | IUCN World Database on Protected Areas, modified (WDPA; <https://protectedplanet.net/>), Riggio *et al*. (2017) | Burton et al., 2012; Schuette et al., 2013 | + |
| Illegal human use | Hum | Targeted or accidental snaring, behavioural avoidance | Probability of site use of illegal human activity^2^ | This study | Burton et al., 2012; Schuette et al., 2013 | - |
| Regular law Enforcement | Enf | Protection from targeted or accidental mortalities, prey base protection | % site with evidence of regular, sustained anti-poaching ^1^ | This study | Burton et al., 2012; Schuette et al., 2013 | + |
| Distance to ranger post | Post | Protection from targeted or accidental mortalities, prey base protection | Distance to nearest range post, with asymptote (Ln) | This study | Burton et al., 2012; Schuette et al., 2013 | - |
| Trophy hunting (permitted) | Hnt | Offtake mortalities | % site where hunting is permitted | Tanzania Wildlife Authority (TAWA) *pers comm*.; this study | Croes et al., 2011 | - |
| Trophy hunting (occurring) | Mgd | Offtake mortalities | % site in actively managed hunting area | Tanzania Wildlife Authority (TAWA) *pers comm*.; this study | Croes et al., 2011 | - |
| ^1^ Covariate not included at the short-term use within the home range scale as it exhibited little or no heterogeneity within most individual home range scale sites, and is therefore not relevant to habitat use decisions at this scale  ^2^ Includes regular protection activities by management authorities (TAWA, TANAPA) or others (e.g. NGOs, hunting operators). Assessed through data collected during surveys, and cross-referenced with management authorities (TAWA, TANAPA) | | | | | | |

Appendix S2 – Spatial Autocorrelation Test Notes & Results Summary

**Additional Methodological Notes**

We employed the methodology detailed by Henschel *et al.* (2016) and Searle *et al.* (2020) to identify, for each species, the minimum sampling occasion length required to avoid spatial autocorrelation, using program PRESENCE (Hines, 2006). Specifically, we increased sampling occasion length by 500 m increments until (1) the standard single-season occupancy (Mackenzie et al., 2002) outperformed the model accounting for Markovian dependence in detections (Hines et al., 2010); and (2) the two key parameters of the model accounting for correlated detections (⊖, probability of presence on a sampling occasion given absence on previous replicate, and ⊖^l^, probability of presence on a sampling occasion given presence on previous replicate) were approximately equal (as per Henschel et al., 2016). The length at which this was achieved was considered the minimum acceptable sampling occasion length for each species, enabling us to avoid spatial auto-correlation biases.

**Results**

For prey species, spatial independence was achieved at 3 – 4km, depending on the species ((4 km: buffalo, roan & sable, impala; 3 km: giraffe, kudu). To streamline analyses, a conservative spatial replicate (sampling occasion) length of 4 km was therefore used for all prey species. For illegal human activity, spatial independence was achieved at 4km, and sampling occasions therefore measured 4 km. For large carnivores, spatial independence was achieved at 1 km for cheetah; 4 km for wild dog and lion; and 5 km for leopard. As a result, a conservative length of 5 km was used for spatial replicates for all large carnivore analyses at the home range scale.

Appendix S3 – Prey & Illegal Human Activity Detection & Site Use Modelling Results

**Prey Detection Model Rankings**

| **Table S3.1.** Model rankings of possible combinations of covariates influencing detection (p) for each prey species at the home range and short-term use scales | | | | | | |
| --- | --- | --- | --- | --- | --- | --- |
| **Species** | **Model** | **AICc** | Δ**AICc** | **W_i_** | **-2*Log** | **nPars** |
| **Home range scale** | | | | | | |
| Buffalo | p (E+S) | 594.76 | 0.00 | 0.36 | 582.76 | 7 |
|  | p (E) | 594.86 | 0.10 | 0.35 | 582.86 | 6 |
|  | p (S) | 596.31 | 1.59 | 0.16 | 584.28 | 6 |
|  | p (.) | 596.75 | 2.05 | 0.13 | 584.71 | 5 |
| Zebra | p (.) | 626.62 | 0.00 | 0.48 | 616.28 | 5 |
|  | p (E) | 627.90 | 1.28 | 0.26 | 615.43 | 6 |
|  | p (S) | 628.74 | 2.12 | 0.17 | 616.27 | 6 |
|  | p (E+S) | 630.04 | 3.42 | 0.09 | 615.41 | 7 |
| Giraffe | p (E) | 670.00 | 0.00 | 0.68 | 657.53 | 6 |
|  | p (E+S) | 671.38 | 1.38 | 0.31 | 671.38 | 7 |
|  | p (.) | 678.15 | 8.15 | 0.01 | 557.81 | 5 |
|  | p (S) | 680.10 | 10.10 | 0.00 | 667.63 | 6 |
| Roan/Sable | p (S) | 559.25 | 0.00 | 0.42 | 544.62 | 6 |
|  | p (E+S) | 560.16 | 0.86 | 0.28 | 543.29 | 7 |
|  | p (.) | 560.68 | 1.43 | 0.21 | 548.21 | 5 |
|  | p (E) | 562.26 | 3.01 | 0.09 | 547.63 | 6 |
| Kudu | p (E+S) | 540.35 | 0.00 | 0.97 | 525.75 | 7 |
|  | p (S) | 546.82 | 6.47 | 0.03 | 534.35 | 6 |
|  | p (E) | 556.58 | 15.84 | 0.00 | 544.11 | 6 |
|  | p (.) | 569.01 | 28.27 | 0.00 | 558.67 | 5 |
| Impala | p (S) | 636.55 | 0.00 | 0.45 | 624.08 | 6 |
|  | p (E+S) | 636.98 | 0.43 | 0.36 | 622.35 | 7 |
|  | p (E) | 639.12 | 2.57 | 0.13 | 626.65 | 6 |
|  | p (.) | 640.63 | 4.08 | 0.06 | 630.29 | 5 |
| **Short-term use scale** | | | | | | |
| Buffalo | p (S) | 2664.83 | 0.00 | 0.69 | 2640.83 | 12 |
|  | p (.) | 2666.45 | 1.62 | 0.21 | 2644.45 | 11 |
| Zebra | p (.) | 2810.10 | 0.00 | 0.81 | 2780.10 | 11 |
|  | p (S) | 2912.02 | 1.92 | 0.19 | 2785.76 | 12 |
| Giraffe | p (S) | 5862.25 | 0.00 | 0.91 | 5836.25 | 9 |
|  | p (.) | 5866.99 | 4.74 | 0.09 | 5846.30 | 8 |
| Roan/Sable | p (.) | 4117.70 | 0.00 | 0.84 | 4095.70 | 11 |
|  | p (S) | 4119.69 | 1.99 | 0.16 | 4095.70 | 12 |
| Kudu | p (S) | 6292.17 | 0.00 | 1.00 | 6260.17 | 13 |
|  | p (.) | 6308.68 | 16.51 | 0.00 | 6278.68 | 12 |
| Impala | p (.) | 3567.93 | 0.00 | 0.83 | 3533.93 | 12 |
|  | p (S) | 3569.91 | 1.98 | 0.17 | 3533.91 | 13 |
| Covariate key: Effort (E); Substrate quality & use index (S). AICc = Akaike Information Criterion, adjusted for small sample sizes; ΔAICc = Difference between model AICc and that of model with the lowest AICc; Wi = relative model weight; nPars = number of parameters in the model; −2 log = twice the negative likelihood; (.) signifies constant parameter | | | | | | |

**Prey Site Use Model Rankings – Home range scale**

| **Table S3.3** Model rankings for prey site use at the home range scale, based on AICc values. Final set based on models with ΔAICc < 2 for (a) buffalo, (b) zebra, (c) giraffe, (d) roan & sable, (e) kudu, and (f) impala | | | | | | | |
| --- | --- | --- | --- | --- | --- | --- | --- |
| 1. **Buffalo** | | | | | | | |
|  | **Model** | **AICc** | Δ**AICc** | **W_i_** | **-2*Log** | **nPars** |  |
| 1 | ψ (PA+W+H) p(E+S) | 593.23 | 0.00 | 1.00 | 578.23 | 7 |  |

| 1. **Zebra** | | | | | | | |
| --- | --- | --- | --- | --- | --- | --- | --- |
|  | **Model** | **AICc** | Δ**AICc** | **W_i_** | **-2*Log** | **nPars** |  |
| 1 | ψ (PA+Enf+NDVIstd) p(.) | 626.62 | 0.00 | 0.26 | 616.28 | 5 |  |
| 2 | ψ (PA+Enf+H) p(.) | 626.83 | 0.21 | 0.23 | 616.49 | 5 |  |
| 3 | ψ (PA+Enf) p(.) | 627.65 | 1.03 | 0.15 | 619.43 | 4 |  |
| 4 | ψ (PA+Enf+C) p(.) | 627.70 | 1.08 | 0.15 | 617.36 | 5 |  |
| 5 | ψ (PA+H+W) p(.) | 628.19 | 1.57 | 0.11 | 617.85 | 5 |  |
| 6 | ψ (PA+Enf+W) p(.) | 628.34 | 1.72 | 0.10 | 618.00 | 5 |  |

| 1. **Giraffe** | | | | | | | |
| --- | --- | --- | --- | --- | --- | --- | --- |
|  | **Model** | **AICc** | Δ**AICc** | **W_i_** | **-2*Log** | **nPars** |  |
| 1 | ψ (PA+NDVIstd+C) p(E) | 668.17 | 0.00 | 0.37 | 655.70 | 6 |  |
| 2 | ψ (PA) p(E) | 669.36 | 1.19 | 0.20 | 661.14 | 4 |  |
| 3 | ψ (PA+W+NDVIstd) p(E) | 670.00 | 1.83 | 0.15 | 657.53 | 6 |  |
| 4 | ψ (PA+W) p(E) | 670.07 | 1.90 | 0.14 | 659.73 | 5 |  |
| 5 | ψ (PA+NDVIstd) p(E) | 670.13 | 1.96 | 0.14 | 659.79 | 5 |  |

| 1. **Roan & Sable** | | | | | | | |
| --- | --- | --- | --- | --- | --- | --- | --- |
|  | **Model** | **AICc** | Δ**AICc** | **W_i_** | **-2*Log** | **nPars** |  |
| 1 | ψ (PA+H+NDVIstd+PA*H) p(S) | 559.25 | 0.00 | 0.39 | 544.62 | 7 |  |
| 2 | ψ (PA+H+Enf+PA*H) p(S) | 560.26 | 1.01 | 0.23 | 545.63 | 7 |  |
| 3 | ψ (PA+H+C+PA*H) p(.) | 560.42 | 1.17 | 0.21 | 545.79 | 7 |  |
| 4 | ψ (PA+H+PA*H) p(.) | 560.93 | 1.68 | 0.17 | 548.46 | 6 |  |

| 1. **Kudu** | | | | | | | |
| --- | --- | --- | --- | --- | --- | --- | --- |
|  | **Model** | **AICc** | Δ**AICc** | **W_i_** | **-2*Log** | **nPars** |  |
| 1 | ψ (Crop) p(E+S) | 539.28 | 0.00 | 0.18 | 528.94 | 5 |  |
| 2 | ψ (Crop+NDVIstd) p(E+S) | 539.36 | 0.08 | 0.17 | 526.89 | 6 |  |
| 3 | ψ (Crop+DistV) p (E+S) | 539.91 | 0.63 | 0.13 | 527.44 | 6 |  |
| 4 | ψ (Crop+C) p (E+S) | 540.21 | 0.93 | 0.11 | 527.74 | 6 |  |
| 5 | ψ (Crop+NDVIstd+DistV) p (E+S) | 540.35 | 1.07 | 0.10 | 525.72 | 7 |  |
| 6 | ψ (Crop+NDVIstd+W) p (E+S) | 540.74 | 1.46 | 0.09 | 526.11 | 7 |  |
| 7 | ψ (Crop+C+DistV) p (E+S) | 540.75 | 1.47 | 0.09 | 526.12 | 7 |  |
| 8 | ψ (C+H) p (E+S) | 541.13 | 1.85 | 0.07 | 528.66 | 6 |  |
| 9 | ψ (Crop+W) p (E+S) | 541.21 | 1.93 | 0.06 | 528.74 | 6 |  |

| 1. **Impala** | | | | | | | |
| --- | --- | --- | --- | --- | --- | --- | --- |
|  | **Model** | **AICc** | Δ**AICc** | **W_i_** | **-2*Log** | **nPars** |  |
| 1 | ψ (Crop+W+C) p(S) | 636.55 | 0.00 | 0.59 | 624.08 | 7 |  |
| 2 | ψ (Crop+H+C) p(S) | 637.24 | 0.69 | 0.41 | 624.77 | 7 |  |
| Covariate key: Legal protection (PA); availability of water (W); primary habitat (H; positive, miombo woodland; negative, acacia-commiphora grasslands & bushlands); strong effective protection (Enf); variation in vegetation composition and growth (NDVIstd); forest cover (C); conversion of habitat to agriculture (Crop); natural logarithm of distance to human settlement (DistV); protected *miombo* woodland (PA*H). AICc = Akaike Information Criterion, adjusted for small sample sizes; ΔAICc = Difference between model AICc and that of model with the lowest AICc; Wi = relative model weight; nPars = number of parameters in the model; −2 log = twice the negative likelihood; (.) signifies constant parameter | | | | | | |  |

**Prey Site Use Model Rankings – Short-term use scale**

| **Table S3.4** Results of model ranking for prey site use at the short-term use scale, based on AIC values. Final set based on models with ΔAICc < 2 for (a) buffalo, (b) zebra, (c) giraffe, (d) roan & sable, (e) kudu, and (f) impala | | | | | | | |
| --- | --- | --- | --- | --- | --- | --- | --- |
| 1. **Buffalo** | | | | | | | |
|  | **Model** | **AIC** | Δ**AIC** | **W_i_** | **-2*Log** | **nPars** |  |
| 1 | ψ,th0(PA+Enf+W+H+C+NDVIstd),th1(),p(S),th0pi() | 2664.83 | 0.00 | 1.00 | 2640.83 | 12 |  |

| 1. **Zebra** | | | | | | | |
| --- | --- | --- | --- | --- | --- | --- | --- |
|  | **Model** | **AIC** | Δ**AIC** | **W_i_** | **-2*Log** | **nPars** |  |
| 1 | ψ,th0(PA+Enf+W+H+C+NDVIstd),th1(),p(.),th0pi() | 2810.10 | 0.00 | 0.28 | 2780.10 | 12 |  |
| 2 | ψ,th0(PA+Enf+W+C+NDVIstd),th1(),p(.),th0pi() | 2811.18 | 1.08 | 0.17 | 2783.18 | 11 |  |
| 3 | ψ,th0(PA+Enf+W+H),th1(),p(.),th0pi() | 2811.22 | 1.12 | 0.16 | 2785.22 | 10 |  |
| 4 | ψ,th0(PA+Enf+W+NDVIstd),th1(),p(.),th0pi() | 2811.56 | 1.46 | 0.14 | 2785.56 | 10 |  |
| 5 | ψ,th0(PA+Enf+W+H+C),th1(),p(.),th0pi() | 2811.60 | 1.50 | 0.13 | 2783.60 | 11 |  |
| 6 | ψ,th0(PA+Enf+W+H+C+NDVIstd),th1(),p(.),th0pi() | 2811.76 | 1.66 | 0.12 | 2783.66 | 11 |  |

| 1. **Giraffe** | | | | | | |
| --- | --- | --- | --- | --- | --- | --- |
|  | **Model** | **AIC** | Δ**AIC** | **W_i_** | **-2*Log** | **nPars** |
| 1 | ψ,th0(PA+Enf+CGE),th1(),p(S),th0pi() | 5860.65 | 0.00 | 0.39 | 5836.65 | 9 |
| 2 | ψ,th0(PA+Enf+CGE+H),th1(),p(S),th0pi() | 5862.25 | 1.60 | 0.18 | 5836.25 | 10 |
| 3 | ψ,th0(PA+Enf+CGE+W),th1(),p(S),th0pi() | 5862.58 | 1.93 | 0.15 | 5836.58 | 10 |
| 4 | ψ,th0(PA+Enf+CGE+C),th1(),p(S),th0pi() | 5862.64 | 1.99 | 0.14 | 5836.64 | 10 |
| 5 | ψ,th0(PA+Enf+CGE+NDVIstd),th1(),p(S),th0pi()th0pi() | 5862.25 | 2.00 | 0.14 | 5836.65 | 10 |

| 1. **Roan & Sable** | | | | | | |
| --- | --- | --- | --- | --- | --- | --- |
|  | **Model** | **AIC** | Δ**AIC** | **W_i_** | **-2*Log** | **nPars** |
| 1 | ψ,th0(PA+Enf+H+C+NDVIstd),th1(),p(.),th0pi() | 4117.58 | 0.00 | 0.52 | 4097.58 | 10 |
| 2 | ψ,th0(PA+Enf+H+C+NDVIstd+W),th1(),p(.),th0pi() | 4117.70 | 0.12 | 0.48 | 4095.70 | 11 |

| 1. **Kudu** | | | | | | |
| --- | --- | --- | --- | --- | --- | --- |
| **Model** | | **AIC** | Δ**AIC** | **W_i_** | **-2*Log** | **nPars** |
| 1 | ψ,th0(PA+Enf+Crop+W+H+C+NDVIstd),th1(),p(S),th0pi() | 6292.17 | 0.00 | 1.00 | 6260.17 | 13 |

| 1. **Impala** | | | | | | |
| --- | --- | --- | --- | --- | --- | --- |
|  | **Model** | **AIC** | Δ**AIC** | **W_i_** | **-2*Log** | **nPars** |
| 1 | ψ,th0(PA+W+Crop+H+C+NDVIstd),th1(),p(.),th0pi() | 3567.75 | 0.00 | 0.52 | 3535.75 | 11 |
| 2 | ψ,th0(PA+Enf+W+Crop+H+C+NDVIstd),th1(),p(.),th0pi() | 3567.93 | 0.18 | 0.48 | 3533.93 | 12 |
| Covariate key: Legal protection (PA); availability of water (W); primary habitat (H; positive, miombo woodland; negative, acacia-commiphora grasslands & bushlands); strong effective protection (Enf); variation in vegetation composition and growth (NDVIstd); forest cover (C); conversion of habitat to agriculture (Crop); natural logarithm of distance to human settlement (DistV); protected *miombo* woodland (PA*H). AICc = Akaike Information Criterion, adjusted for small sample sizes; ΔAICc = Difference between model AICc and that of model with the lowest AICc; Wi = relative model weight; nPars = number of parameters in the model; −2 log = twice the negative likelihood; (.) signifies constant parameter | | | | | | |

**Prey Site Use Covariate Effects – Home range scale**

| **Table S3.5** Relative summed model weights (Σw) and *β*-coefficient estimates, with associated standard errors, of covariates explaining site use (ψ) of six ungulate species in Ruaha-Rungwa at the home range scale. Only models retained in the final confidence set (ΔAICc<2) were considered. Highlighted covariates have a significant effect (*β* ± 1.96 x SE). | | | |
| --- | --- | --- | --- |
| **Site Covariate** | **Σw** | **Relationship** | ***β* (SE*_β_*)** |
| **Buffalo** | | | |
| PA | 1.00 | + | **1.73 (0.40)** |
| W | 1.00 | + | **1.13 (0.39)** |
| H | 1.00 | + | **1.10 (0.34)** |
| **Zebra** | | | |
| PA | 1.00 | + | **1.04 (0.30)** |
| Enf | 0.89 | + | **0.67 (0.23)** |
| W | 0.21 | + | **0.47 (0.20)** |
| H | 0.34 | + | 0.33 (0.20) |
| NDVIstd | 0.23 | + | 0.35 (0.20) |
| C | 0.15 | + | 0.29 (0.21) |
| **Giraffe** | | | |
| PA | 1.00 | + | **2.46 (0.45)** |
| NDVIstd | 0.66 | - | **1.23 (0.57)** |
| C | 0.37 | + | 1.73 (0.89) |
| W | 0.29 | - | 0.50 (0.34) |
| **Roan & Sable** | | | |
| PA | 1.00 | + | **1.52 (0.30)** |
| H | 1.00 | + | **1.37 (0.31)** |
| PA*H | 1.00 | + | **1.72 (0.68)** |
| NDVIstd | 0.39 | + | **0.63 (0.30)** |
| Enf | 0.23 | - | 0.49 (0.28) |
| C | 0.21 | + | 0.57 (0.33) |
| **Kudu** | | | |
| Crop | 1.00 | - | **1.80 (0.30)** |
| NDVIstd | 0.36 | - | 0.50 (0.36) |
| DistV | 0.32 | + | 0.58 (0.48) |
| C | 0.20 | - | 0.42 (0.39) |
| W | 0.15 | - | 0.30 (0.33) |
| H | 0.07 | - | 0.17 (0.33) |
| **Impala** | | | |
| Crop | 1.00 | - | **1.63 (0.31)** |
| C | 1.00 | - | **1.78 (0.33)** |
| W | 0.59 | + | **0.75 (0.29)** |
| H | 0.41 | - | **0.74 (0.31)** |
| Covariate key: Legal protection (PA); availability of water (W); primary habitat (H; + = miombo woodland; - = *Acacia-Commiphora* grasslands & bushlands); regular law enforcement (Enf); variation in vegetation composition and growth (NDVIstd); forest cover (C); conversion of habitat to agriculture (Crop); logarithmic distance to human settlement (DistV); protected miombo woodland (PA*H) | | | |

At the home range scale, legal protection and availability of natural habitat were the best predictors of occurrence for all prey species. Of these, legal protection was a better predictor of occurrence of buffalo, zebra, giraffe, and sable and roan, while, for kudu and impala availability of natural habitat explained occurrence best. This suggests that while kudu and impala can persist without legal protection, provided natural habitat is available, the same is not true for the other species. Dry season surface water availability was a strong predictor of some water-dependent species (buffalo, zebra and impala), and a weak or even negative (kudu) predictor for the others. Primary habitat type (miombo woodlands or Acacia-Commiphora bushland and grasslands) was important for some species, with statistically significant between buffalo and roan & sable site use and miombo, and between impala occurrence and Acacia-Commiphora. Regular law enforcement was a strong predictor of zebra persistence, and significant associations were observed between greater forest cover and buffalo and roan & sable site use, while kudu and impala instead exhibited significant positive associations with more open areas. Greater variation in vegetation composition and growth was a strong positive predictor of roan & sable; giraffe and impala, instead, were conversely strongly associated with areas of lower primary productivity. Similar relationships were observed at the short-term use scale (Table S3.6), as were a range of other non-significant covariate effects, at both spatial scales.

**Prey Site Use Covariate Effects – Short-term use scale**

| **Table S3.6** Relative summed model weights (Σw) and *β*-coefficient estimates, with associated standard errors, of covariates explaining site use (ψ) of six ungulate species in Ruaha-Rungwa at the short-term use scale. Only models retained in the final confidence set (ΔAICc<2) were considered. Highlighted covariates have a significant effect (*β* ± 1.96 x SE). | | | |
| --- | --- | --- | --- |
| **Site Covariate** | **Σw** | **Relationship** | ***β* (SE*_β_*)** |
| **Buffalo** | | | |
| PA | 1.00 | + | **1.23 (0.35)** |
| Enf | 1.00 | + | **0.13 (0.05)** |
| W | 1.00 | + | **0.58 (0.06)** |
| H | 1.00 | + | **0.18 (0.06)** |
| C | 1.00 | - | **0.21 (0.06)** |
| NDVIstd | 1.00 | + | **0.21 (0.07)** |
| **Zebra** | | | |
| PA | 1.00 | + | **1.25 (0.40)** |
| Enf | 1.00 | + | **0.47 (0.07)** |
| W | 1.00 | + | **0.17 (0.05)** |
| NDVIstd | 0.71 | + | 0.13 (0.07) |
| H | 0.69 | + | 0.11 (0.06) |
| C | 0.58 | - | 0.13 (0.07) |
| **Giraffe** | | | |
| PA | 1.00 | + | **0.96 (0.08)** |
| Enf | 1.00 | + | **0.14 (0.05)** |
| Crop | 1.00 | - | **0.21 (0.09)** |
| H | 0.18 | - | 0.03 (0.05) |
| W | 0.15 | - | 0.01 (0.04) |
| C | 0.14 | - | 0.01 (0.06) |
| NDVIstd | 0.14 | - | 0.01 (0.03) |
| **Roan & Sable** | | | |
| PA | 1.00 | + | **1.15 (0.12)** |
| Enf | 1.00 | - | **0.27 (0.05)** |
| H | 1.00 | + | **0.54 (0.06)** |
| C | 1.00 | + | **0.28 (0.06)** |
| NDVIstd | 1.00 | + | **0.14 (0.06)** |
| W | 0.48 | - | 0.07 (0.05) |
| **Kudu** | | | |
| PA | 1.00 | + | **2.41 (0.76)** |
| Crop | 1.00 | - | **0.45 (0.09)** |
| W | 1.00 | - | **0.32 (0.11)** |
| H | 1.00 | - | **0.80 (0.15)** |
| C | 1.00 | - | **0.45 (0.16)** |
| NDVIstd | 1.00 | + | **0.52 (0.13)** |
| Enf | 1.00 | - | 1.43 (0.86) |
| **Impala** | | | |
| PA | 1.00 | + | **0.56 (0.09)** |
| Crop | 1.00 | - | **0.47 (0.09)** |
| W | 1.00 | + | **0.36 (0.08)** |
| H | 1.00 | - | **0.55 (0.08)** |
| C | 1.00 | - | **0.67 (0.10)** |
| NDVIstd | 1.00 | - | **0.27 (0.10)** |
| Enf | 0.48 | + | 0.05 (0.06) |
| Covariate key: Legal protection (PA); availability of water (W); primary habitat (H; + = miombo woodland; - = *Acacia-Commiphora* grasslands & bushlands); regular on law enforcement (Enf); variation in vegetation composition and growth (NDVIstd); forest cover (C); conversion of habitat to agriculture (Crop); logarithmic distance to human settlement (DistV); protected miombo woodland (PA*H) | | | |

**Illegal Human Activity Detection Model Rankings**

| **Table S3.2.** Model rankings of possible combinations of covariates influencing detection (p) illegal human activity at the home range and short-term use scales | | | | | |
| --- | --- | --- | --- | --- | --- |
| **Model** | **AICc** | Δ**AICc** | **W_i_** | **-2*Log** | **nPars** |
| **Home range scale** | | | | | |
| p (.) | 501.47 | 0.00 | 0.49 | 486.84 | 7 |
| p (S) | 503.03 | 1.56 | 0.22 | 486.21 | 8 |
| p (E) | 503.30 | 1.83 | 0.20 | 486.48 | 8 |
| p (E+S) | 504.96 | 3.49 | 0.09 | 485.93 | 9 |
| **Short-term use scale** | | | | | |
| p (.) | 1995.26 | 0.00 | 0.73 | 1973.26 | 11 |
| p (S) | 1997.26 | 2.00 | 0.27 | 1973.26 | 12 |

**Illegal Human Activity Site Use Model Rankings**

| **Table S3.7** Results of multivariate model ranking for illegal human site use at the home range and short-term use scales, based on AICc values. Final set based on all models with ΔAICc < 2 | | | | | | |
| --- | --- | --- | --- | --- | --- | --- |
|  | **Model** | **AICc** | Δ**AICc** | **W_i_** | **-2*Log** | **nPars** |
| **Home range scale** | | | | | | |
| 1 | ψ (Bound+Enf+C+Post) p(.) | 500.43 | 0.00 | 0.19 | 487.96 | 6 |
| 2 | ψ (Bound+Enf+Post) p(.) | 500.57 | 0.14 | 0.18 | 490.23 | 5 |
| 3 | ψ (Bound+Enf+W+Post) p(.) | 501.08 | 0.65 | 0.14 | 488.61 | 6 |
| 4 | ψ (Bound+Enf+W+C+Post) p(.) | 501.47 | 1.04 | 0.12 | 486.84 | 7 |
| 5 | ψ (Bound+Enf+C) p(.) | 501.66 | 1.23 | 0.11 | 491.32 | 5 |
| 6 | ψ (Bound+Enf+W+C) p(.) | 501.93 | 1.50 | 0.09 | 489.46 | 6 |
| 7 | ψ (Bound+Enf+C+Post+AllPrey) p(.) | 502.00 | 1.57 | 0.09 | 487.37 | 7 |
| 8 | ψ (Bound+Enf+Post+AllPrey) p(.) | 502.26 | 1.83 | 0.08 | 489.79 | 6 |
| **Short-term use scale** | | | | | | |
| 1 | ψ (Bound+Enf+Post+W+C+AllPrey) p(.) | 1995.26 | 0.00 | 1.00 | 1973.26 | 11 |
| Covariate key: Availability of water (W); strong effective protection (Enf); forest cover (C); distance to protected area boundary (Bound); distance to ranger post (Post); mean availability of ungulate prey (AllPrey). AICc = Akaike Information Criterion, adjusted for small sample sizes; ΔAICc = Difference between model AICc and that of model with the lowest AICc; Wi = relative model weight; nPars = number of parameters in the model; −2 log = twice the negative likelihood; (.) signifies constant parameter | | | | | | |

**Illegal Human Activity Covariate Effects**

| **Table S3.8** Relative summed model weights (Σw) and *β*-coefficient estimates, with associated standard errors, of covariates explaining illegal human site use (ψ) at the home range and short-term use scales within PAs in Ruaha-Rungwa. Only models retained in the final confidence set (ΔAICc<2) were considered. Highlighted covariates have a significant effect (*β* ± 1.96 x SE) in the top and/or the most parameterised model in which they appear. | | | |
| --- | --- | --- | --- |
| **Site Covariate** | **Σw** | **Relationship** | ***β* (SE*_β_*)** |
| **Home range scale** | | | |
| Bound | 1.00 | - | **1.67 (0.52)** |
| Enf | 1.00 | - | **1.06 (0.38)** |
| Post | 0.80 | + | 0.63 (0.36) |
| C | 0.60 | - | 0.49 (0.36) |
| W | 0.35 | - | 0.35 (0.28) |
| AllPrey | 0.17 | - | 0.34 (0.49) |
| **Short-term use scale** | | | |
| Post | 1.00 | + | **0.44 (0.06)** |
| Bound | 1.00 | - | **0.78 (0.09)** |
| Enf | 1.00 | - | **0.91 (0.13)** |
| W | 1.00 | + | **0.19 (0.07)** |
| C | 1.00 | + | **0.13 (0.05)** |
| AllPrey | 1.00 | - | **0.21 (0.05)** |
| Covariate key: Logarithmic distance to PA boundary (Bound); regular law enforcement (Enf); distance to ranger post (Post); forest cover (C); availability of water (W); mean availability of ungulate prey (AllPrey) | | | |

At the home range scale, higher illegal human use was most strongly associated with proximity to PA boundary and a lack of law enforcement, which both had a significant impact. There was also some evidence that greater illegal human use was associated with increased distance to ranger post and greater forest cover, and to a lesser extent with lower availability of water and of ungulates, although none of these had a significant effect. At the short-term use scale, all included predictor covariates exhibited a significant relationship with illegal human activity, and their summed model weights were equal. Illegal human activity was strongly positively associated with increasing distance from ranger post, proximity to PA boundary and availability of water, and negatively with regular law enforcement, more closed areas, and availability of wild ungulates.

Appendix S4 – Large Carnivore Detection & Site Use Modelling Results

**Large Carnivores Detection Model Rankings**

| **Table S4.1.** Model rankings of covariates influencing detection (p) for lion in occupancy analyses at the home range and short-term use scales | | | | | | |
| --- | --- | --- | --- | --- | --- | --- |
| **Analysis** | **Model** | **AICc** | Δ**AICc** | **W_i_** | **-2*Log** | **nPars** |
| Home range scale  (all PAs) | p (E+S) | 541.92 | 0.00 | 0.89 | 527.88 | 7 |
|  | p (S) | 545.95 | 4.11 | 0.11 | 533.99 | 6 |
|  | p (E) | 575.61 | 33.77 | 0.00 | 563.65 | 6 |
|  | p (.) | 584.44 | 42.60 | 0.00 | 574.48 | 5 |
| Home range scale  (hunting areas) | p (E+S) | 312.88 | 0.00 | 0.89 | 298.88 | 7 |
|  | p (S) | 317.10 | 4.22 | 0.11 | 305.10 | 6 |
|  | p (E) | 331.72 | 18.84 | 0.00 | 319.72 | 6 |
|  | p (.) | 337.73 | 24.85 | 0.00 | 327.73 | 5 |
| Short-term use scale | p (S) | 2070.75 | 0.00 | 1.00 | 2052.75 | 9 |
|  | p (.) | 2093.19 | 22.44 | 0.00 | 2075.19 | 8 |
| Covariate key: Effort (E); Substrate quality & use index (S); AICc = Akaike Information Criterion, adjusted for small sample sizes; ΔAICc = Difference between model AICc and that of model with the lowest AICc; Wi = relative model weight; nPars = number of parameters in the model; −2 log = twice the negative likelihood; (.) signifies constant parameter | | | | | | |

| **Table S4.2.** Model rankings of covariates influencing detection (p) for leopard in occupancy analyses at the home range and short-term use scales | | | | | | |
| --- | --- | --- | --- | --- | --- | --- |
| **Analysis** | **Model** | **AICc** | Δ**AICc** | **W_i_** | **-2*Log** | **nPars** |
| Home range scale  (all PAs) | p (E+S) | 635.25 | 0.00 | 0.96 | 623.30 | 6 |
|  | p (S) | 641.78 | 6.48 | 0.04 | 631.78 | 5 |
|  | p (E) | 658.55 | 23.25 | 0.00 | 648.55 | 5 |
|  | p (.) | 669.70 | 34.40 | 0.00 | 661.70 | 4 |
| Home range scale  (hunting areas) | p (E+S) | 390.99 | 0.00 | 0.89 | 376.99 | 7 |
|  | p (S) | 395.29 | 4.30 | 0.10 | 383.29 | 6 |
|  | p (E) | 399.52 | 8.53 | 0.01 | 387.52 | 6 |
|  | p (.) | 405.35 | 14.36 | 0.00 | 395.35 | 5 |
| Short-term use scale | p (S) | 2916.84 | 0.00 | 1.00 | 2989.84 | 9 |
|  | p (.) | 2952.82 | 35.98 | 0.00 | 2936.82 | 8 |
| Covariate key: Effort (E); Substrate quality & use index (S). AICc = Akaike Information Criterion, adjusted for small sample sizes; ΔAICc = Difference between model AICc and that of model with the lowest AICc; Wi = relative model weight; nPars = number of parameters in the model; −2 log = twice the negative likelihood; (.) signifies constant parameter | | | | | | |

| **Table S4.3.** Model rankings of covariates influencing detection (p) for cheetah in occupancy analyses at the home range and short-term use scales | | | | | | |
| --- | --- | --- | --- | --- | --- | --- |
| **Analysis** | **Model** | **AICc** | Δ**AICc** | **W_i_** | **-2*Log** | **nPars** |
| Home range scale  (all PAs) | p (E) | 107.33 | 0.00 | 0.42 | 99.33 | 4 |
|  | p (.) | 107.62 | 0.29 | 0.36 | 101.62 | 3 |
|  | p (E+S) | 109.29 | 1.96 | 0.16 | 99.29 | 5 |
|  | p (S) | 111.00 | 3.67 | 0.06 | 103.00 | 4 |
| Short-term use scale | p (.) | 143.40 | 0.00 | 0.56 | 137.40 | 3 |
|  | p (S) | 143.91 | 0.51 | 0.44 | 135.91 | 4 |
| Covariate key: Effort (E); Substrate quality & use index (S). AICc = Akaike Information Criterion, adjusted for small sample sizes; ΔAICc = Difference between model AICc and that of model with the lowest AICc; Wi = relative model weight; nPars = number of parameters in the model; −2 log = twice the negative likelihood; (.) signifies constant parameter | | | | | | |

| **Table S4.4.** Model rankings of covariates influencing detection (p) for wild dog in occupancy analyses at the home range and short-term use scales | | | | | | |
| --- | --- | --- | --- | --- | --- | --- |
| **Analysis** | **Model** | **AICc** | Δ**AICc** | **W_i_** | **-2*Log** | **nPars** |
| Home range scale  (all PAs) | p (E+S) | 250.66 | 0.00 | 0.54 | 238.66 | 6 |
|  | p (E) | 251.46 | 0.80 | 0.36 | 241.46 | 5 |
|  | p (S) | 254.37 | 3.71 | 0.08 | 244.37 | 5 |
|  | p (.) | 257.01 | 6.35 | 0.02 | 249.01 | 4 |
| Home range scale  (hunting areas) | p (.) | 161.08 | 0.00 | 0.36 | 153.08 | 4 |
|  | p (E) | 161.47 | 0.39 | 0.29 | 151.47 | 5 |
|  | p (S) | 162.14 | 1.06 | 0.21 | 152.14 | 5 |
|  | p (E+S) | 162.92 | 1.84 | 0.14 | 150.92 | 6 |
| Short-term use | p (S) | 2468.83 | 0.00 | 0.99 | 2456.83 | 9 |
|  | p (.) | 2479.38 | 10.55 | 0.01 | 2469.38 | 8 |
| Covariate key: Effort (E); Substrate quality & use index (S). AICc = Akaike Information Criterion, adjusted for small sample sizes; ΔAICc = Difference between model AICc and that of model with the lowest AICc; Wi = relative model weight; nPars = number of parameters in the model; −2 log = twice the negative likelihood; (.) signifies constant parameter | | | | | | |

| **Table S4.5.** Model rankings of covariates influencing detection (p) for spotted hyaena at the short-term use scale | | | | | | |
| --- | --- | --- | --- | --- | --- | --- |
| **Analysis** | **Model** | **AICc** | Δ**AICc** | **W_i_** | **-2*Log** | **nPars** |
| Short-term use scale | p (S) | 2070.75 | 0.00 | 1.00 | 2052.75 | 9 |
|  | p (.) | 2093.19 | 22.44 | 0.00 | 2075.19 | 8 |
| Covariate key: Effort (E); Substrate quality & use index (S); AICc = Akaike Information Criterion, adjusted for small sample sizes; ΔAICc = Difference between model AICc and that of model with the lowest AICc; Wi = relative model weight; nPars = number of parameters in the model; −2 log = twice the negative likelihood; (.) signifies constant parameter | | | | | | |

**Large Carnivore Site Use Model Rankings**

The final model set for each large carnivore occupancy analysis can be found below. Additionally, complete model rankings, for all species and at all scales, can be accessed at: <https://github.com/RuahaRungwaOccupancy/FullModelRankings>

***Lion***

| **Table S4.6.** Results of model ranking for lion site use, based on AICc/AIC values. Final set of models with ΔAICc/ΔAIC < 2 for all occupancy analyses at the home range and short-term use scales | | | | | | | |
| --- | --- | --- | --- | --- | --- | --- | --- |
| 1. **Home range scale (all PAs)** | | | | | | | |
|  | **Model** | **AICc** | Δ**AICc** | **W_i_** | **-2*Log** | **nPars** |  |
| 1 | ψ (Hum+H+Mgd) p(E+S) | 541.92 | 0.00 | 0.10 | 527.92 | 7 |  |
| 2 | ψ (Enf+H+Mgd) p(E+S) | 542.08 | 0.16 | 0.09 | 528.08 | 7 |  |
| 3 | ψ (Hum+H+Post+Mgd) p(E+S) | 542.53 | 0.61 | 0.07 | 526.53 | 8 |  |
| 4 | ψ (Enf+H+Post+Mgd) p(E+S) | 542.63 | 0.71 | 0.07 | 526.63 | 8 |  |
| 5 | ψ (Hum+H+C+Mgd) p(E+S) | 542.93 | 1.00 | 0.06 | 526.93 | 8 |  |
| 6 | ψ (Hum+Mgd) p(E+S) | 543.06 | 1.14 | 0.05 | 531.06 | 6 |  |
| 7 | ψ (Buff+Post+Mgd) p(E+S) | 543.09 | 1.17 | 0.05 | 529.09 | 7 |  |
| 8 | ψ (Hum+R+H+Mgd) p(E+S) | 543.21 | 1.29 | 0.05 | 527.21 | 8 |  |
| 9 | ψ (Hum+Post+Mgd) p(E+S) | 543.21 | 1.29 | 0.05 | 529.21 | 7 |  |
| 10 | ψ (Enf+Post+Mgd) p(E+S) | 543.40 | 1.47 | 0.05 | 529.40 | 7 |  |
| 11 | ψ (Enf+R+M+Mgd) p(E+S) | 543.46 | 1.54 | 0.04 | 527.46 | 8 |  |
| 12 | ψ (Enf+M+C+Mgd) p(E+S) | 543.52 | 1.59 | 0.04 | 527.52 | 8 |  |
| 13 | ψ (Hum) p(E+S) | 543.55 | 1.63 | 0.04 | 533.55 | 5 |  |
| 14 | ψ (Enf+Mgd) p(E+S) | 543.56 | 1.64 | 0.04 | 531.56 | 6 |  |
| 15 | ψ (Hum+R+Mgd) p(E+S) | 543.68 | 1.76 | 0.04 | 529.68 | 7 |  |
| 16 | ψ (Hum+M+C+Post+Mgd) p(E+S) | 543.68 | 1.76 | 0.04 | 525.68 | 9 |  |
| 17 | ψ (Enf) p(E+S) | 543.79 | 1.86 | 0.04 | 533.79 | 5 |  |
| 18 | ψ (Hum+R+M+C+Mgd) p(E+S) | 543.89 | 1.97 | 0.04 | 525.89 | 9 |  |
| 19 | ψ (Hum+Buff+M+Mgd) p(E+S) | 543.90 | 1.98 | 0.04 | 527.90 | 8 |  |

| 1. **Home range scale (hunting areas)** | | | | | | | |
| --- | --- | --- | --- | --- | --- | --- | --- |
|  | **Model** | **AICc** | Δ**AICc** | **W_i_** | **-2*Log** | **nPars** |  |
| 1 | ψ (H+Mgd) p(E+S) | 311.30 | 0.00 | 0.19 | 299.30 | 6 |  |
| 2 | ψ (Mgd) p(E+S) | 311.70 | 0.40 | 0.15 | 301.70 | 5 |  |
| 3 | ψ (Enf+H+Mgd) p(E+S) | 312.99 | 1.68 | 0.09 | 298.99 | 7 |  |
| 4 | ψ (H+Post+Mgd) p(E+S) | 313.05 | 1.74 | 0.08 | 299.05 | 7 |  |
| 5 | ψ (Hum+H+Mgd) p(E+S) | 313.14 | 1.84 | 0.07 | 299.14 | 7 |  |
| 6 | ψ (SecPrey+H+Mgd) p(E+S) | 313.19 | 1.88 | 0.07 | 299.19 | 7 |  |
| 7 | ψ (AllPrey+H+Mgd) p(E+S) | 313.20 | 1.89 | 0.07 | 299.20 | 7 |  |
| 8 | ψ (R+H+Mgd) p(E+S) | 313.21 | 1.90 | 0.07 | 299.21 | 7 |  |
| 9 | ψ (Buff+H+Mgd) p(E+S) | 313.23 | 1.92 | 0.07 | 299.23 | 7 |  |
| 10 | ψ (Bound+H+Mgd) p(E+S) | 313.25 | 1.95 | 0.07 | 299.25 | 7 |  |
| 11 | ψ (H+C+Mgd) p(E+S) | 313.30 | 2.00 | 0.07 | 299.30 | 7 |  |

| 1. **Short-term use scale** | | | | | | | |
| --- | --- | --- | --- | --- | --- | --- | --- |
|  | **Model** | **AIC** | Δ**AIC** | **W_i_** | **-2*Log** | **nPars** |  |
| 1 | ψ (Buff+R+Imp),θ_0_, θ_1~~,~~_ p(S) | 2070.75 | 0.00 | 0.23 | 2052.75 | 9 |  |
| 2 | ψ (Buff+R+Sbl),θ_0_, θ_1~~,~~_ p(S) | 2071.16 | 0.41 | 0.15 | 2053.16 | 9 |  |
| 3 | ψ (Buff+R+Sbl+Kud),θ_0_, θ_1~~,~~_ p(S) | 2071.42 | 0.67 | 0.12 | 2051.42 | 10 |  |
| 4 | ψ (Buff+R+Imp+Kud),θ_0_, θ_1~~,~~_ p(S) | 2071.99 | 1.24 | 0.11 | 2051.99 | 10 |  |
| 5 | ψ (Buff+R+Kud),θ_0_, θ_1~~,~~_ p(S) | 2072.11 | 1.36 | 0.1 | 2054.11 | 9 |  |
| 6 | ψ (Buff+R+Post+Kud),θ_0_, θ_1~~,~~_ p(S) | 2072.25 | 1.5 | 0.09 | 2052.25 | 10 |  |
| 7 | ψ (Buff+R),θ_0_, θ_1~~,~~_ p(S) | 2072.6 | 1.85 | 0.07 | 2056.6 | 8 |  |
| 8 | ψ (Buff+R+Imp+Post),θ_0_, θ_1~~,~~_ p(S) | 2072.63 | 1.88 | 0.06 | 2052.63 | 10 |  |
| 9 | ψ (Buff+R+Imp+Bound),θ_0_, θ_1~~,~~_ p(S) | 2072.63 | 1.88 | 0.04 | 2052.63 | 10 |  |
| 10 | ψ (Buff+R+Imp+Hum),θ_0_, θ_1~~,~~_ p(S) | 2072.73 | 1.98 | 0.03 | 2052.73 | 10 |  |
| Covariate key: Mean prey site use (AllPrey); mean prey site use of secondary prey (SecPrey); buffalo site use (Buff); kudu site use (Kud); impala site use (Imp); roan & sable site use (Sbl); availability of riparian habitat (R); distance to ranger post (Post); distance to PA boundary (Bound); probability of illegal human activity (Hum); primary habitat (H; positive, miombo woodland; negative, *Acacia-commiphora* grasslands & bushlands); evidence of regular law enforcement (Enf); forest and bush cover (C); site actively hunted (Mgd). AICc = Akaike Information Criterion, adjusted for small sample sizes; ΔAICc = Difference between model AICc and that of model with the lowest AICc; Wi = relative model weight; nPars = number of parameters in the model; −2 log = twice the negative likelihood; (.) signifies constant parameter | | | | | | |  |

***Leopard***

| **Table S4.7.** Results of model ranking for leopard site use, based on AICc/AIC values. Final set of models with ΔAICc/ΔAIC < 2 for all occupancy analyses at the home range and short-term use scales | | | | | | | |
| --- | --- | --- | --- | --- | --- | --- | --- |
| 1. **Home range scale (all PAs)** | | | | | | | |
|  | **Model** | **AICc** | Δ**AICc** | **W_i_** | **-2*Log** | **nPars** |  |
| 1 | ψ (Enf+Mgd) p(E+S) | 635.25 | 0.00 | 0.31 | 623.25 | 6 |  |
| 2 | ψ (Enf+R+Mgd) p(E+S) | 636.11 | 0.85 | 0.20 | 622.11 | 7 |  |
| 3 | ψ (Enf+H+Mgd) p(E+S) | 636.90 | 1.64 | 0.14 | 622.90 | 7 |  |
| 4 | ψ (Enf+Imp+Mgd) p(E+S) | 637.10 | 1.84 | 0.12 | 623.10 | 7 |  |
| 5 | ψ (Enf+C+Mgd) p(E+S) | 637.18 | 1.93 | 0.12 | 623.18 | 7 |  |
| 6 | ψ (Enf+Post+Mgd) p(E+S) | 637.23 | 1.98 | 0.11 | 623.23 | 7 |  |

| 1. **Home range scale (hunting areas)** | | | | | | | |
| --- | --- | --- | --- | --- | --- | --- | --- |
|  | **Model** | **AICc** | Δ**AICc** | **W_i_** | **-2*Log** | **nPars** |  |
| 1 | ψ (Hum+Post+Mgd) p(E+S) | 390.99 | 0.00 | 0.13 | 376.99 | 7 |  |
| 2 | ψ (Imp+Mgd) p(E+S) | 391.35 | 0.36 | 0.11 | 379.35 | 6 |  |
| 3 | ψ (Enf+Imp+Mgd) p(E+S) | 391.50 | 0.51 | 0.10 | 377.50 | 7 |  |
| 4 | ψ (Hum+Imp+Mgd) p(E+S) | 391.55 | 0.57 | 0.10 | 377.55 | 7 |  |
| 5 | ψ (Hum+Post) p(E+S) | 391.63 | 0.64 | 0.09 | 379.63 | 6 |  |
| 6 | ψ (Enf+Post+Mgd) p(E+S) | 391.96 | 0.97 | 0.08 | 377.96 | 7 |  |
| 7 | ψ (Bound+Imp+Mgd) p(E+S) | 392.30 | 1.31 | 0.07 | 378.30 | 7 |  |
| 8 | ψ (R+Imp+Mgd) p(E+S) | 392.43 | 1.44 | 0.06 | 378.43 | 7 |  |
| 9 | ψ (Post+Mgd) p(E+S) | 392.51 | 1.52 | 0.06 | 380.51 | 6 |  |
| 10 | ψ (Mgd) p(E+S) | 392.76 | 1.77 | 0.05 | 382.76 | 5 |  |
| 11 | ψ (Hum+R+Post) p(E+S) | 392.81 | 1.82 | 0.05 | 378.81 | 7 |  |
| 12 | ψ (Enf+Mgd) p(E+S) | 392.87 | 1.88 | 0.05 | 380.87 | 6 |  |
| 13 | ψ (Hum+M+Post) p(E+S) | 392.92 | 1.93 | 0.05 | 378.92 | 7 |  |

| 1. **Short-term use scale** | | | | | | | |
| --- | --- | --- | --- | --- | --- | --- | --- |
|  | **Model** | **AIC** | Δ**AIC** | **W_i_** | **-2*Log** | **nPars** |  |
| 1 | ψ (Kud+C+Bound),θ_0_, θ_1~~,~~_ p(S) | 2916.84 | 0.00 | 0.11 | 2898.84 | 9 |  |
| 2 | ψ (C+Bound),θ_0_, θ_1~~,~~_ p(S) | 2917.17 | 0.33 | 0.09 | 2901.17 | 8 |  |
| 3 | ψ (C+Bound+Post),θ_0_, θ_1~~,~~_ p(S) | 2917.61 | 0.77 | 0.08 | 2899.61 | 9 |  |
| 4 | ψ (Kud+Imp+Bound),θ_0_, θ_1~~,~~_ p(S) | 2917.62 | 0.78 | 0.08 | 2899.62 | 9 |  |
| 5 | ψ (Kud+C+Bound+Post),θ_0_, θ_1~~,~~_ p(S) | 2918.01 | 1.17 | 0.06 | 2898.01 | 10 |  |
| 6 | ψ (Bound),θ_0_, θ_1~~,~~_ p(S) | 2918.05 | 1.21 | 0.06 | 2904.05 | 7 |  |
| 7 | ψ (AllPrey+Imp+Bound),θ_0_, θ_1~~,~~_ p(S) | 2918.09 | 1.25 | 0.06 | 2900.09 | 9 |  |
| 8 | ψ (Kud+R+C+Bound),θ_0_, θ_1~~,~~_ p(S) | 2918.12 | 1.28 | 0.06 | 2898.12 | 10 |  |
| 9 | ψ (AllPrey+C+Bound),θ_0_, θ_1~~,~~_ p(S) | 2918.16 | 1.32 | 0.06 | 2900.16 | 9 |  |
| 10 | ψ (Imp+Bound),θ_0_, θ_1~~,~~_ p(S) | 2918.22 | 1.38 | 0.06 | 2902.22 | 8 |  |
| 11 | ψ (AllPrey+C+Bound+Post),θ_0_, θ_1~~,~~_ p(S) | 2918.27 | 1.43 | 0.05 | 2898.27 | 10 |  |
| 12 | ψ (Bound+Post),θ_0_, θ_1~~,~~_ p(S) | 2918.34 | 1.50 | 0.05 | 2902.34 | 8 |  |
| 13 | ψ (R+C+Bound),θ_0_, θ_1~~,~~_ p(S) | 2918.55 | 1.71 | 0.05 | 2900.55 | 9 |  |
| 14 | ψ (Kud+Bound),θ_0_, θ_1~~,~~_ p(S) | 2918.66 | 1.82 | 0.04 | 2902.66 | 8 |  |
| 15 | ψ (AllPrey+Kud+C+Bound),θ_0_, θ_1~~,~~_ p(S) | 2918.70 | 1.86 | 0.04 | 2898.70 | 10 |  |
| 16 | ψ (AllPrey+Kud+Imp+Bound),θ_0_, θ_1~~,~~_ p(S)),θ_0_, θ_1~~,~~_ p(S) | 2918.82 | 1.98 | 0.04 | 2898.82 | 10 |  |
| Covariate key: Man prey site use (AllPrey); kudu site use (Kud); impala site use (Imp); availability of riparian habitat (R); distance to ranger post (Post); distance to PA boundary (Bound); probability of illegal human activity (Hum); primary habitat (H; positive, miombo woodland; negative, *Acacia-commiphora* grasslands & bushlands); evidence of regular law enforcement (Enf); forest and bush cover (C); site in hunting area (Hnt); site actively hunted (Mgd) | | | | | | |  |

***Cheetah***

| **Table S4.8.** Results of model ranking for cheetah site use, based on AICc/AIC values. Final set of models with ΔAICc/ΔAIC < 2 for all occupancy analyses at the home range and short-term use scales | | | | | | | |
| --- | --- | --- | --- | --- | --- | --- | --- |
| 1. **Home range scale (all PAs)** | | | | | | | |
|  | **Model** | **AICc** | Δ**AICc** | **W_i_** | **-2*Log** | **nPars** |  |
| 1 | ψ (.) p(E+S) | 106.96 | 0.00 | 0.14 | 100.96 | 3 |  |
| 2 | ψ (Hum) p(E+S) | 107.33 | 0.37 | 0.12 | 99.33 | 4 |  |
| 3 | ψ (Enf) p(E+S) | 107.61 | 0.65 | 0.10 | 99.61 | 4 |  |
| 4 | ψ (Riv) p(E+S) | 107.68 | 0.72 | 0.10 | 99.68 | 4 |  |
| 5 | ψ (C) p(E+S) | 107.88 | 0.92 | 0.09 | 99.88 | 4 |  |
| 6 | ψ (AllPrey) p(E+S) | 108.10 | 1.14 | 0.08 | 100.10 | 4 |  |
| 7 | ψ (Post) p(E+S) | 108.35 | 1.39 | 0.07 | 100.35 | 4 |  |
| 8 | ψ (Imp) p(E+S) | 108.45 | 1.49 | 0.07 | 100.45 | 4 |  |
| 9 | ψ (Hnt) p(E+S) | 108.73 | 1.77 | 0.06 | 100.73 | 4 |  |
| 10 | ψ (Mgd) p(E+S) | 108.76 | 1.8 | 0.06 | 100.76 | 4 |  |
| 11 | ψ (M) p(E+S) | 108.88 | 1.92 | 0.05 | 100.88 | 4 |  |
| 12 | ψ (R) p(E+S) | 108.92 | 1.96 | 0.05 | 100.92 | 4 |  |

| 1. **Short-term use scale** | | | | | | | |
| --- | --- | --- | --- | --- | --- | --- | --- |
|  | **Model** | **AIC** | Δ**AIC** | **W_i_** | **-2*Log** | **nPars** |  |
| 1 | ψ (.) p(.) | 143.14 | 0.00 | 0.18 | 139.14 | 2 |  |
| 2 | ψ (R) p(.) | 143.4 | 0.26 | 0.16 | 137.40 | 3 |  |
| 3 | ψ (Bound) p(.) | 144.52 | 1.38 | 0.09 | 138.52 | 3 |  |
| 4 | ψ (AllPrey) p(.) | 144.92 | 1.78 | 0.08 | 138.92 | 3 |  |
| 5 | ψ (Post) p(.) | 144.93 | 1.79 | 0.08 | 138.93 | 3 |  |
| 6 | ψ (Sbl) p(.) | 145.02 | 1.88 | 0.07 | 139.02 | 3 |  |
| 7 | ψ (Riv) p(.) | 145.09 | 1.95 | 0.07 | 139.09 | 3 |  |
| 8 | ψ (Imp) p(.) | 145.1 | 1.96 | 0.07 | 139.10 | 3 |  |
| 9 | ψ (C) p(.) | 145.13 | 1.99 | 0.07 | 139.13 | 3 |  |
| 10 | ψ (Kud) p(.) | 145.14 | 2.00 | 0.07 | 139.14 | 3 |  |
| 11 | ψ (Hum) p(.) | 145.14 | 2.00 | 0.06 | 139.14 | 3 |  |
| Covariate key: Mean prey site use (AllPrey); kudu site use (Kud); impala site use (Imp); roan & sable site use (Sbl); availability of riparian habitat (R); distance to ranger post (Post); distance to PA boundary (Bound); probability of illegal human activity (Hum); primary habitat (H; positive, miombo woodland; negative, *Acacia-commiphora* grasslands & bushlands); evidence of regular law enforcement (Enf); forest and bush cover (C); site in hunting area (Hnt); site actively hunted (Mgd); distance to large river (Riv). AICc = Akaike Information Criterion, adjusted for small sample sizes; ΔAICc = Difference between model AICc and that of model with the lowest AICc; Wi = relative model weight; nPars = number of parameters in the model; −2 log = twice the negative likelihood; (.) signifies constant parameter | | | | | | |  |

***Wild Dog***

| **Table S4.9.** Results of model ranking for wild dog site use, based on AICc/AIC values. Final set of models with ΔAICc/ΔAIC < 2 for all occupancy analyses at the home range and short-term use scales | | | | | | | |
| --- | --- | --- | --- | --- | --- | --- | --- |
| 1. **Home range scale (all P9As)** | | | | | | | |
|  | **Model** | **AICc** | Δ**AICc** | **W_i_** | **-2*Log** | **nPars** |  |
| 1 | ψ (AllPrey+Riv) p(E+S) | 250.66 | 0.00 | 1.00 | 238.66 | 6 |  |

| 1. **Home range scale (hunting areas)** | | | | | | | |
| --- | --- | --- | --- | --- | --- | --- | --- |
|  | **Model** | **AICc** | Δ**AICc** | **W_i_** | **-2*Log** | **nPars** |  |
| 1 | ψ (AllPrey+Riv) p(S) | 161.08 | 0.00 | 0.48 | 153.08 | 4 |  |
| 2 | ψ (AllPrey+M) p(S) | 162.10 | 1.02 | 0.29 | 154.10 | 4 |  |
| 3 | ψ (AllPrey) p(S) | 162.51 | 1.42 | 0.23 | 156.51 | 3 |  |

| 1. **Short-term use scale** | | | | | | | |
| --- | --- | --- | --- | --- | --- | --- | --- |
|  | **Model** | **AIC** | Δ**AIC** | **W_i_** | **-2*Log** | **K** |  |
| 1 | ψ (C+Kud+Bound), θ_0_, θ_1_, p(S) | 2468.83 | 0.00 | 0.36 | 456.83 | 6 |  |
| 2 | ψ (Kud+Bound) p(.), θ_0_, θ_1_, p(S) | 2469.70 | 0.87 | 0.24 | 457.70 | 6 |  |
| 3 | ψ (Kud+Imp) p(.), θ_0_, θ_1_, p(S) | 2469.71 | 0.88 | 0.23 | 459.71 | 5 |  |
| 4 | ψ (R+Kud+Bound), θ_0_, θ_1_, p(S) | 2470.32 | 1.48 | 0.17 | 458.32 | 6 |  |
| Covariate key: Mean prey site use (AllPrey); kudu site use (Kud); impala site use (Imp); availability of riparian habitat (R); distance to ranger post (Post); distance to PA boundary (Bound); probability of illegal human activity (Hum); primary habitat (H; positive, miombo woodland; negative, *Acacia-commiphora* grasslands & bushlands); evidence of regular law enforcement (Enf); forest and bush cover (C); site in hunting area (Hnt); distance to large river (Riv). AICc = Akaike Information Criterion, adjusted for small sample sizes; ΔAICc = Difference between model AICc and that of model with the lowest AICc; Wi = relative model weight; nPars = number of parameters in the model; −2 log = twice the negative likelihood; (.) signifies constant parameter | | | | | | |  |

***Spotted Hyaena***

| **Table S4.10.** Results of model ranking for spotted hyaena site use at the short-term use scale, based on AIC values. Final set of models with ΔAIC < 2 | | | | | | | |
| --- | --- | --- | --- | --- | --- | --- | --- |
| 1. **TUS** | | | | | | | |
|  | **Model** | **AIC** | Δ**AIC** | **W_i_** | **-2*Log** | **K** |  |
| 1 | ψ (Buff+Sbl), θ_0_, θ_1_, p(S) | 5264.58 | 0.00 | 0.36 | 5248.58 | 8 |  |
| 2 | ψ (Buff+Sbl+Hum) p(.), θ_0_, θ_1_, p(S) | 5265.90 | 1.32 | 0.18 | 5247.90 | 9 |  |
| 3 | ψ (Buff+R+Sbl) p(.), θ_0_, θ_1_, p(S) | 5266.08 | 1.50 | 0.17 | 5248.08 | 9 |  |
| 4 | ψ (Buff+Sbl+Kud) p(.), θ_0_, θ_1_, p(S) | 5266.25 | 1.67 | 0.15 | 5248.25 | 9 |  |
| 5 | ψ (Buff+C+Sbl), θ_0_, θ_1_, p(S) | 5266.48 | 1.90 | 0.14 | 5248.48 | 9 |  |
| 6 | ψ (Buff+Sbl), θ_0_, θ_1_, p(S) | 5264.58 | 0.00 | 0.36 | 5248.58 | 8 |  |
| Covariate key: Mean prey site use (AllPrey); kudu site use (Kud); buffalo site use (Buff); kudu site use (Kud); roan & sable roan site use (Sbl); availability of riparian habitat (R); distance to ranger post (Post); probability of illegal human activity (Hum); primary habitat (H; positive, miombo woodland; negative, *Acacia-commiphora* grasslands & bushlands); forest and bush cover (C). AICc = Akaike Information Criterion, adjusted for small sample sizes; ΔAICc = Difference between model AICc and that of model with the lowest AICc; Wi = relative model weight; nPars = number of parameters in the model; −2 log = twice the negative likelihood; (.) signifies constant parameter | | | | | | |  |

Appendix S5 – Additional Maps & Figures

**Ruaha-Rungwa Hunting Blocks**

Ruaha-Rungwa’s hunting areas, including information on block status (actively managed/vacant) at the time of study.


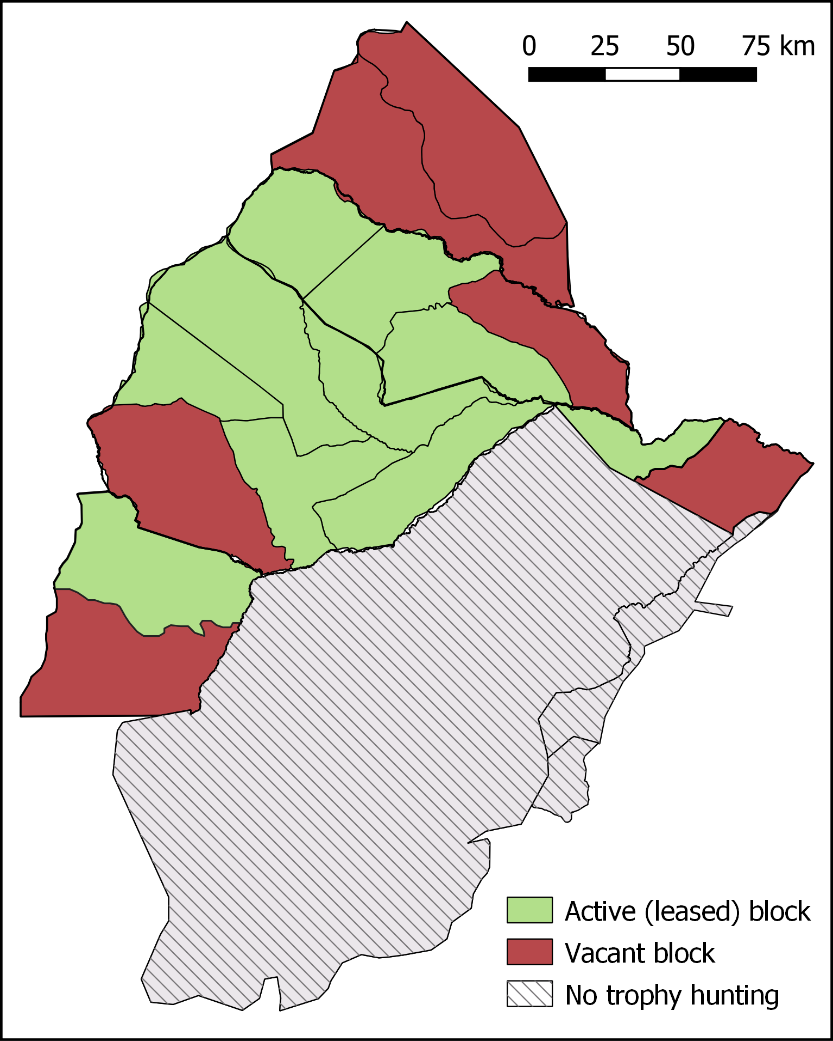


Lunda-Mkwambi S

Kizigo East 1

Lunda-Mkwambi N

Kizigo Central

Muhesi West

Kizigo East 2

Wembere South

Rungwa Rungwa (W)

Rungwa Rungwa (E)

Rungwa Mzombe

Rungwa South

Rungwa Mwamagembe

Rungwa Mpera

Rungwa Inyonga

Muhesi East

**Detections**

***Illegal Human Activity Detections***

Illegal human activity detections, across the landscape (A) and superimposed over hunting block status (B).


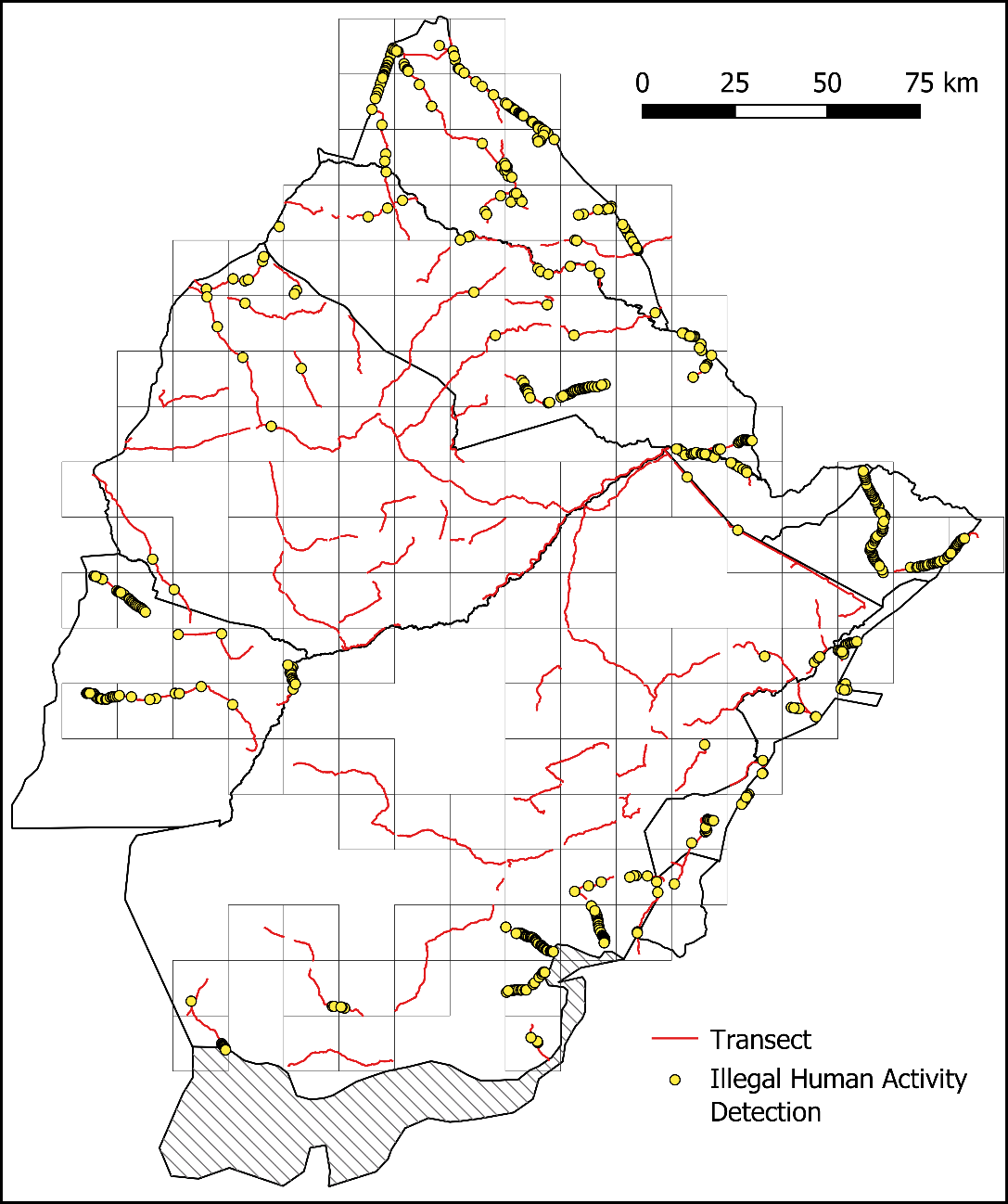


**A**


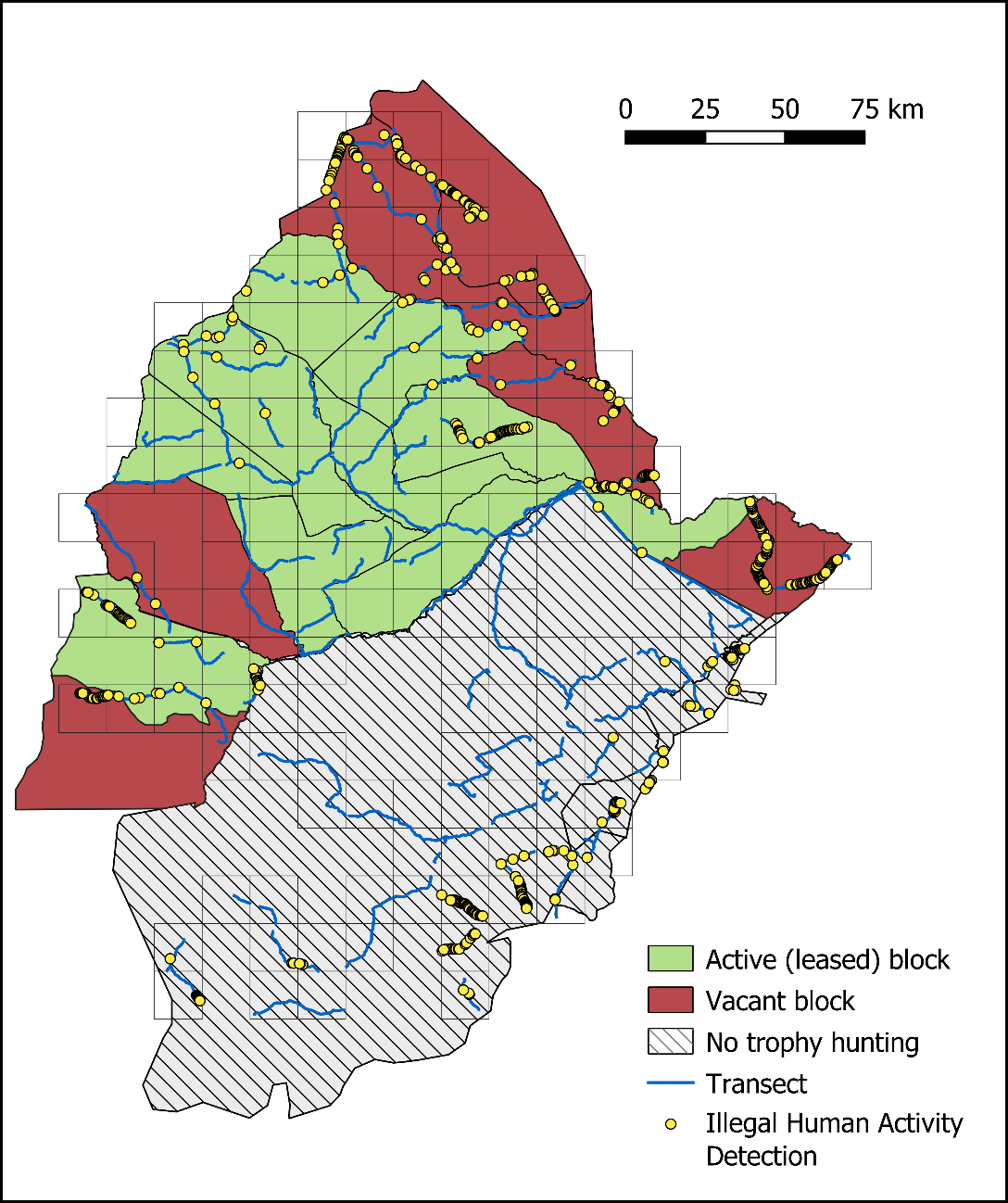


**B**

***Large Carnivore Detections***

*Lion*


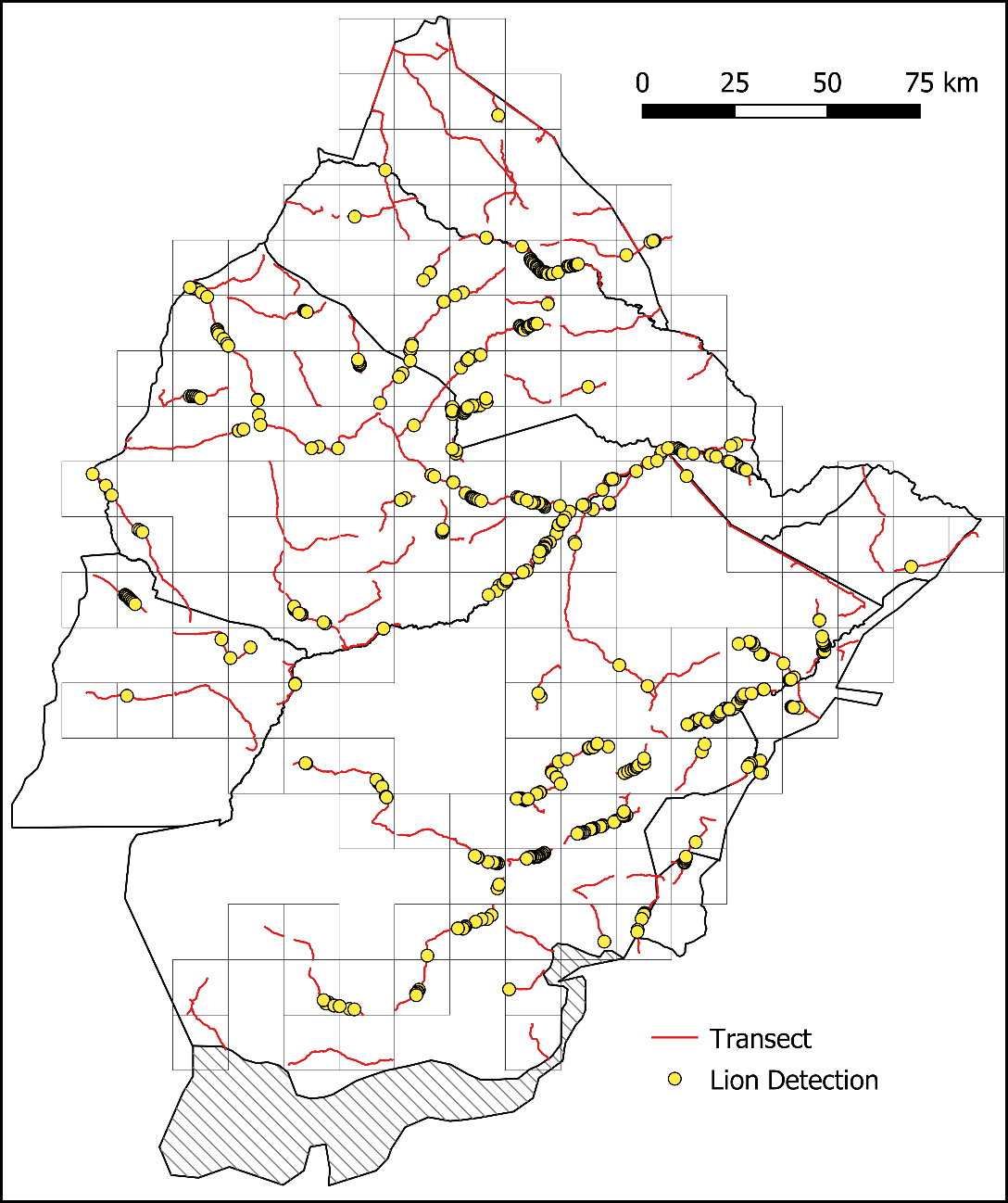


*Leopard*


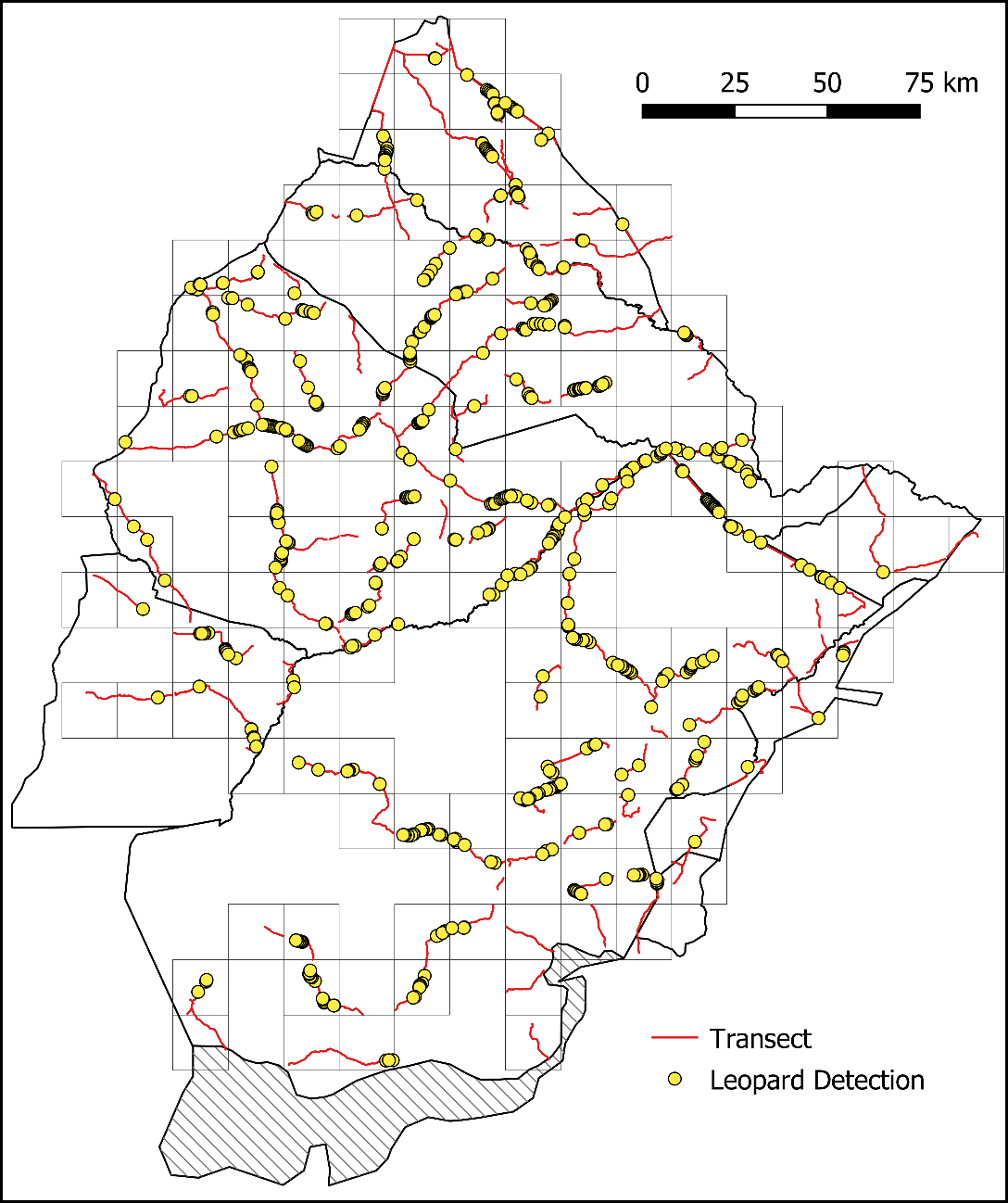


*Cheetah*


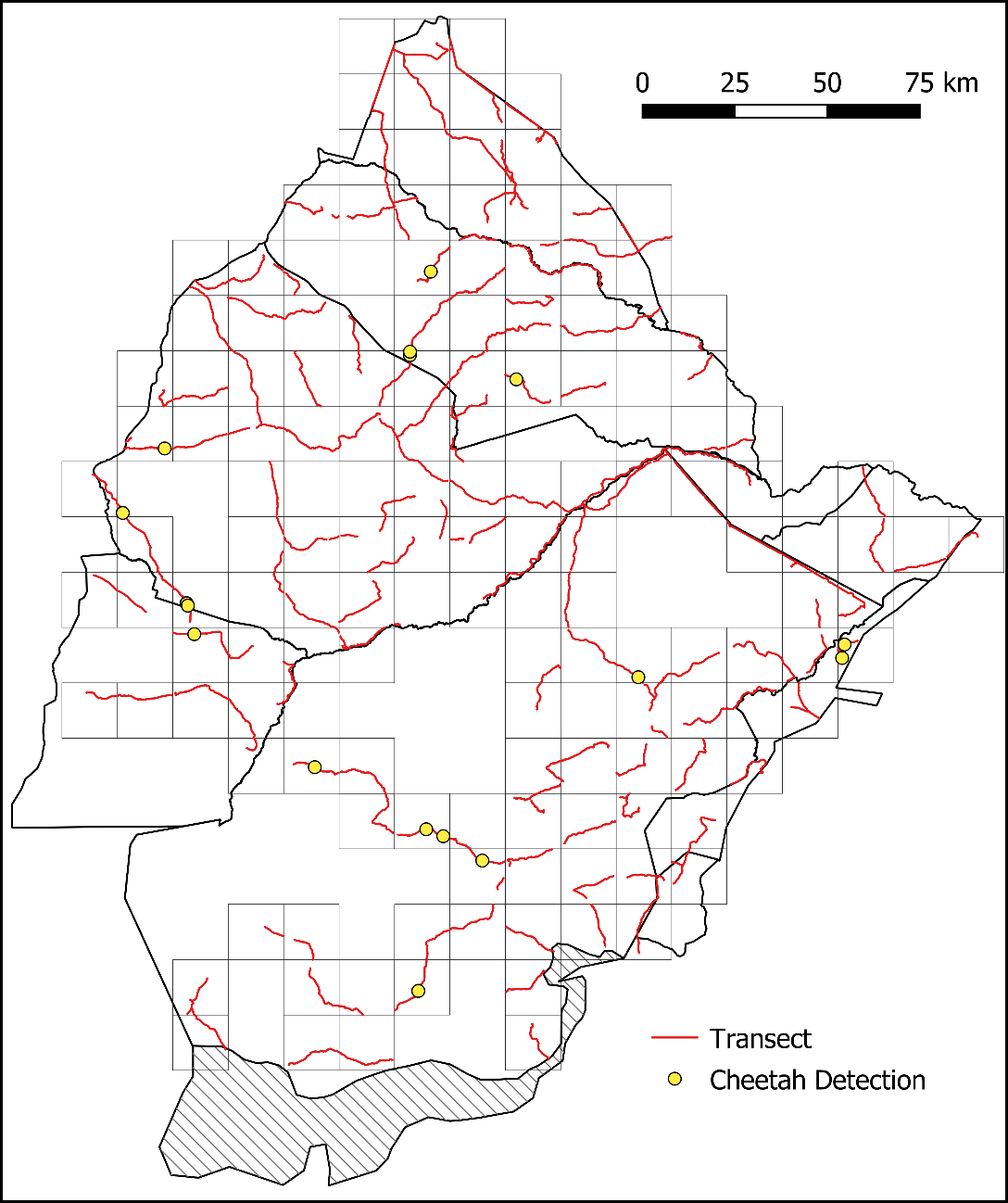


*African Wild Dog*


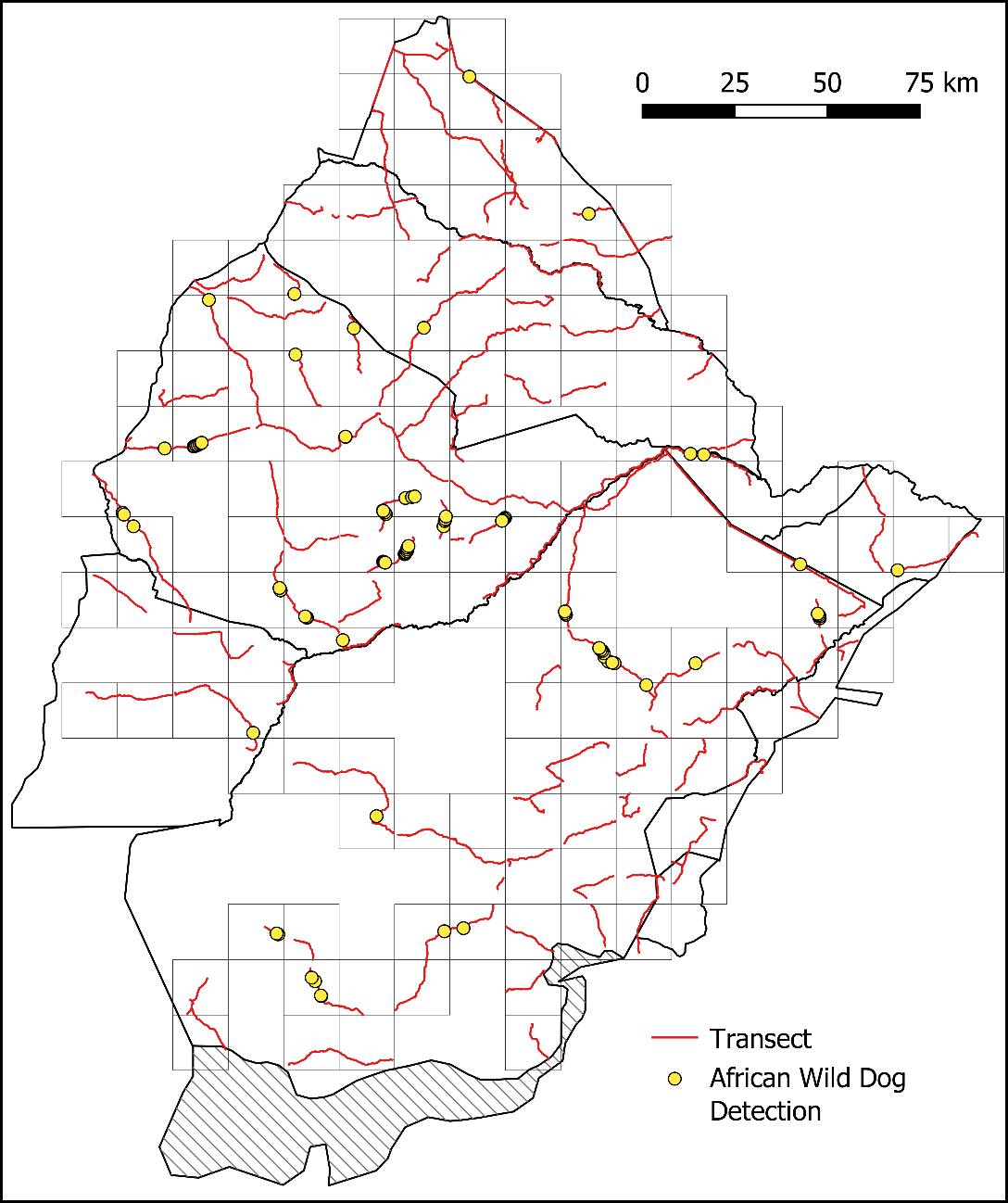


*Spotted Hyaena*


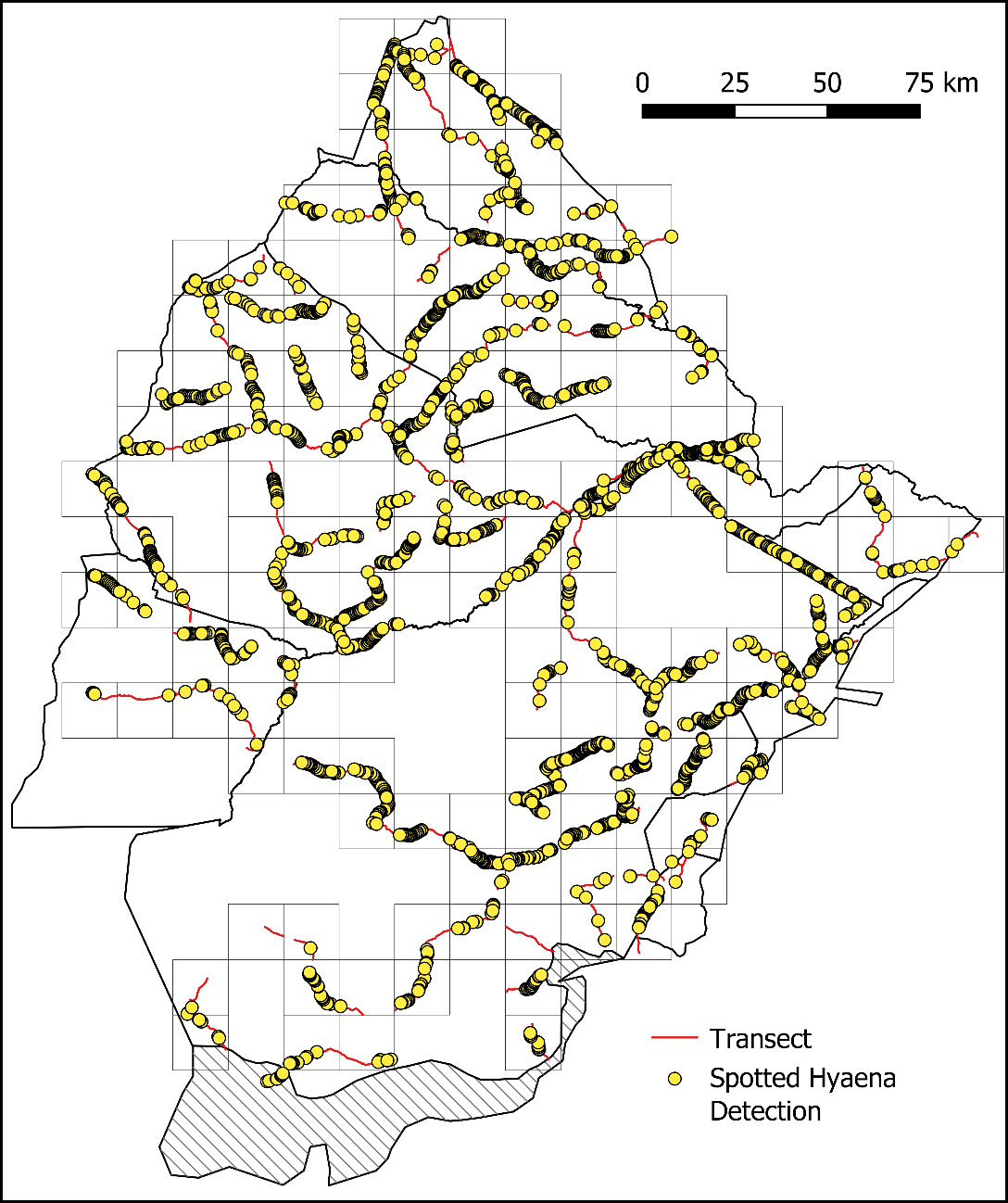


**Probability of Site Use Maps**

All maps are derived from the home range scale analysis covering all PAs.

***Ungulate Prey***

*Buffalo probability of site use*

**
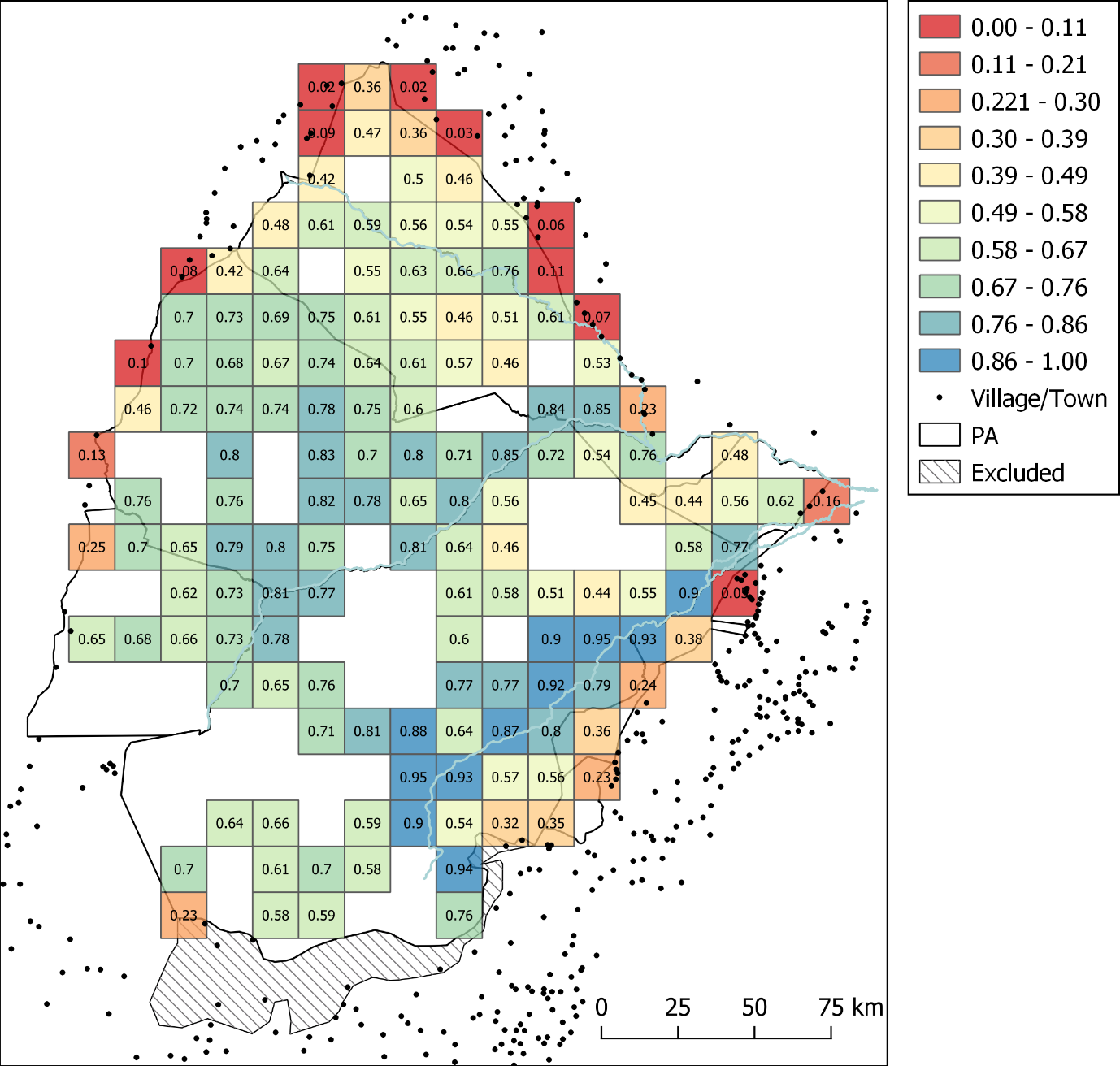
**

*Giraffe probability of site use*

**
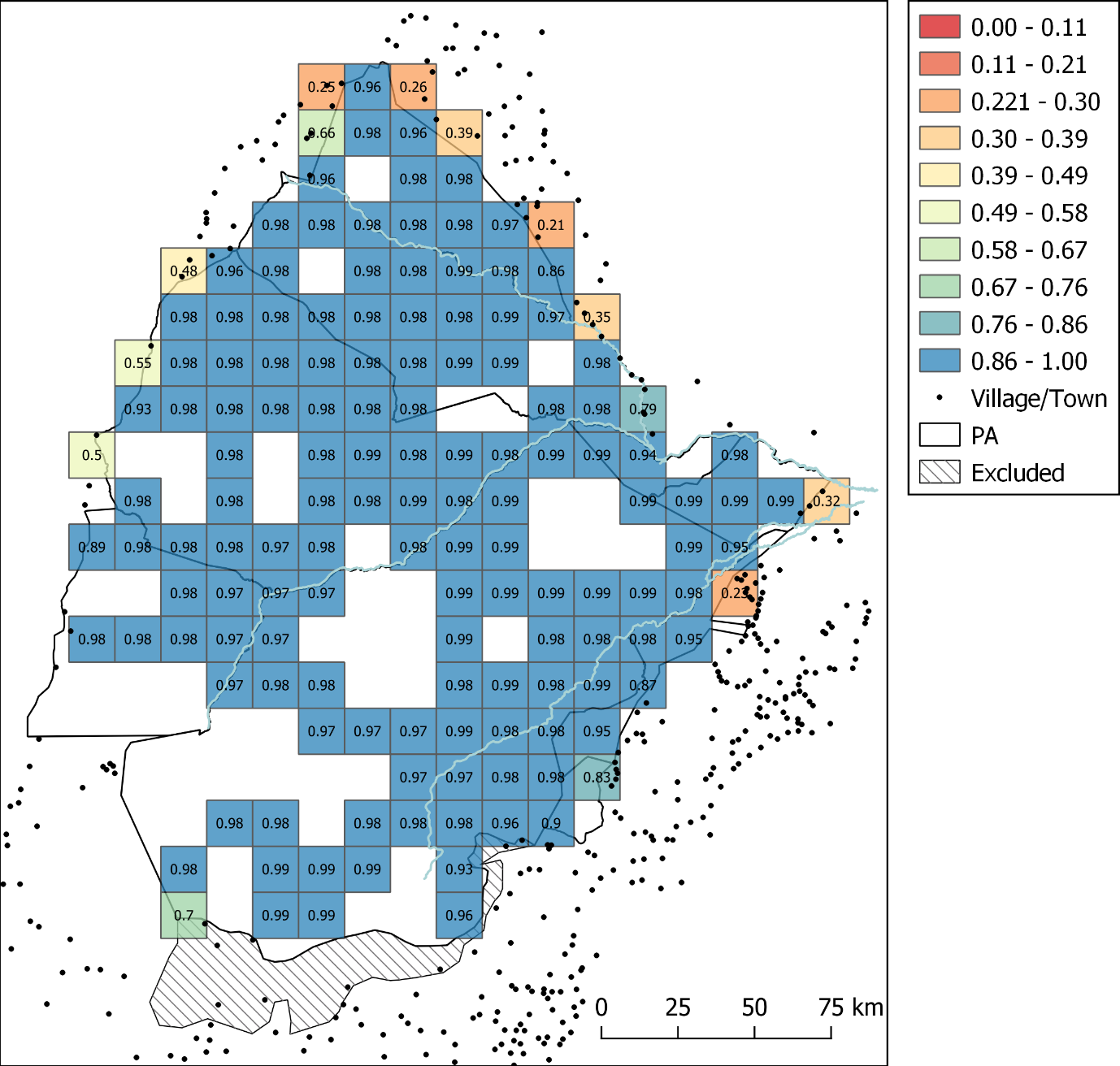
**

*Zebra probability of site use*

**
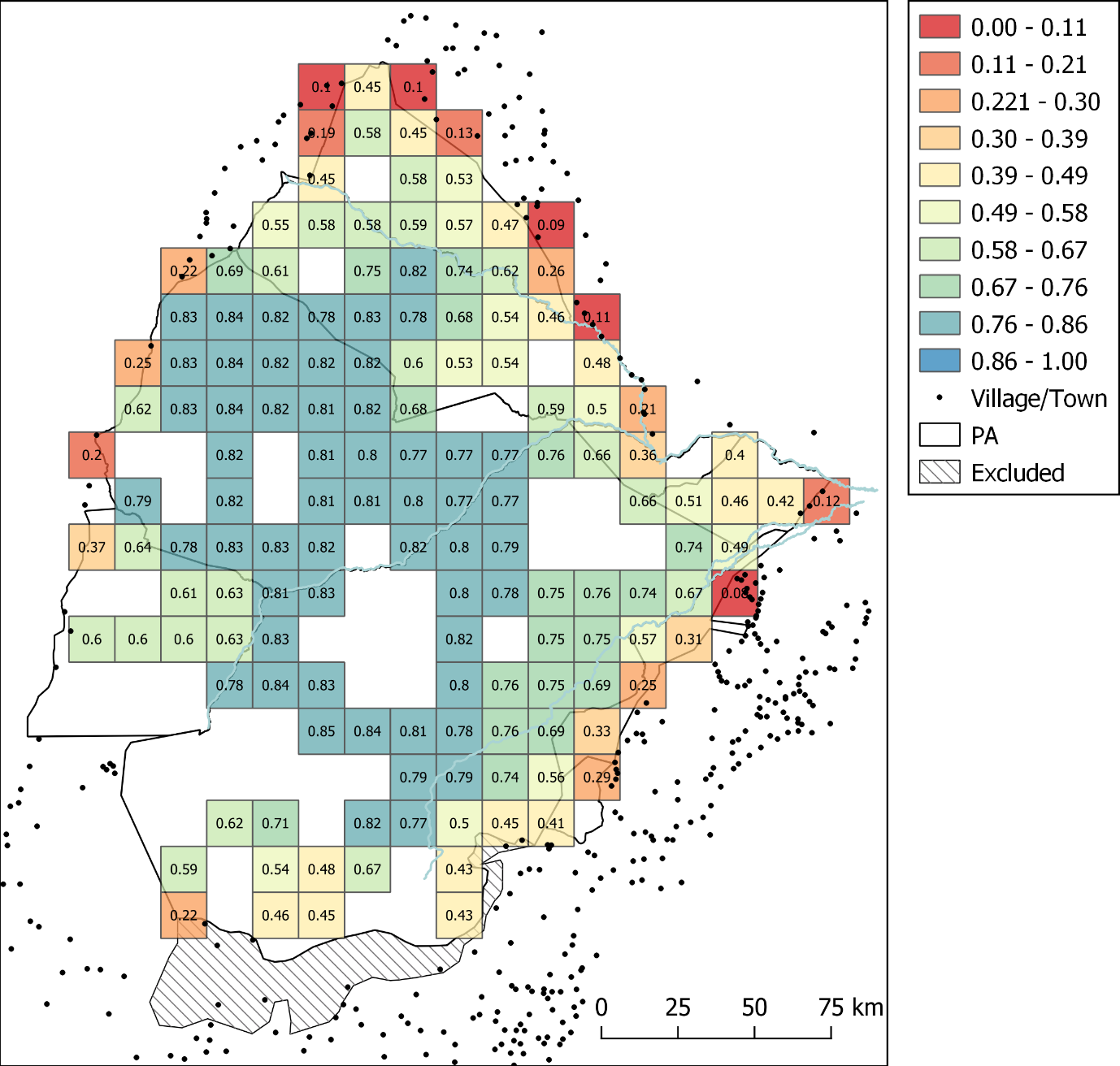
**

*Roan & Sable probability of site use*

**
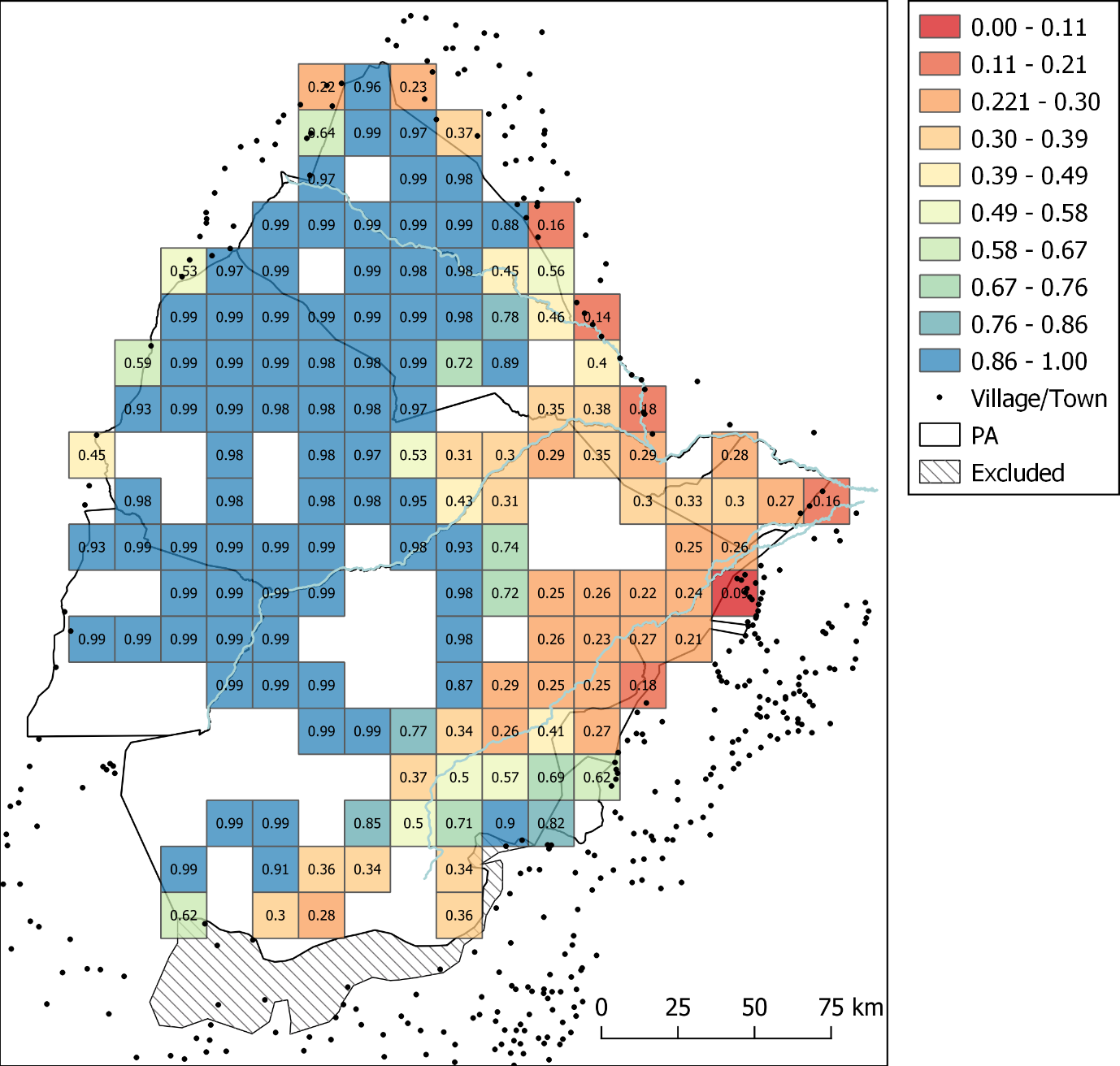
**

*Greater kudu probability of site use*

**
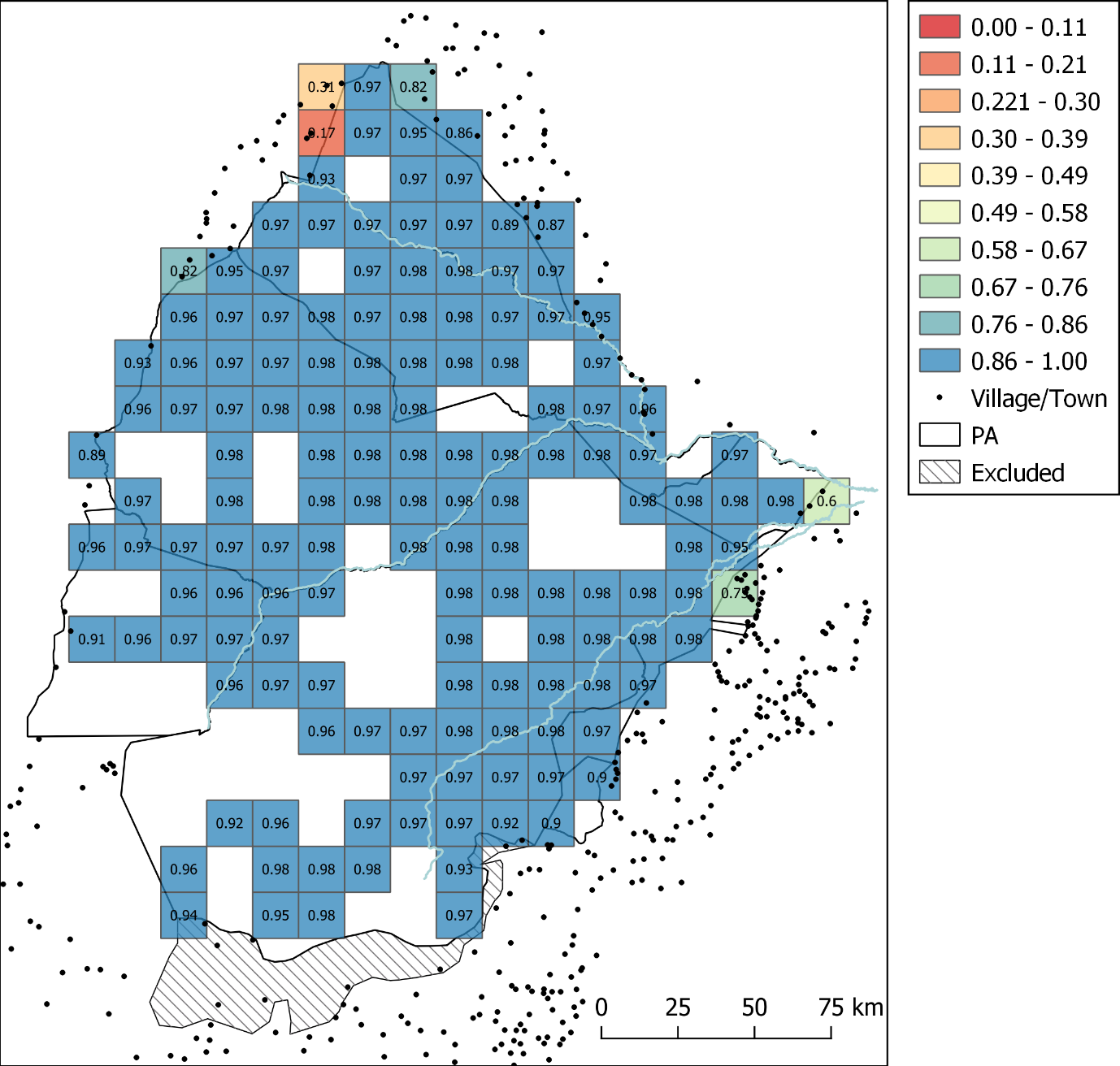
**

*Impala probability of site use*

**
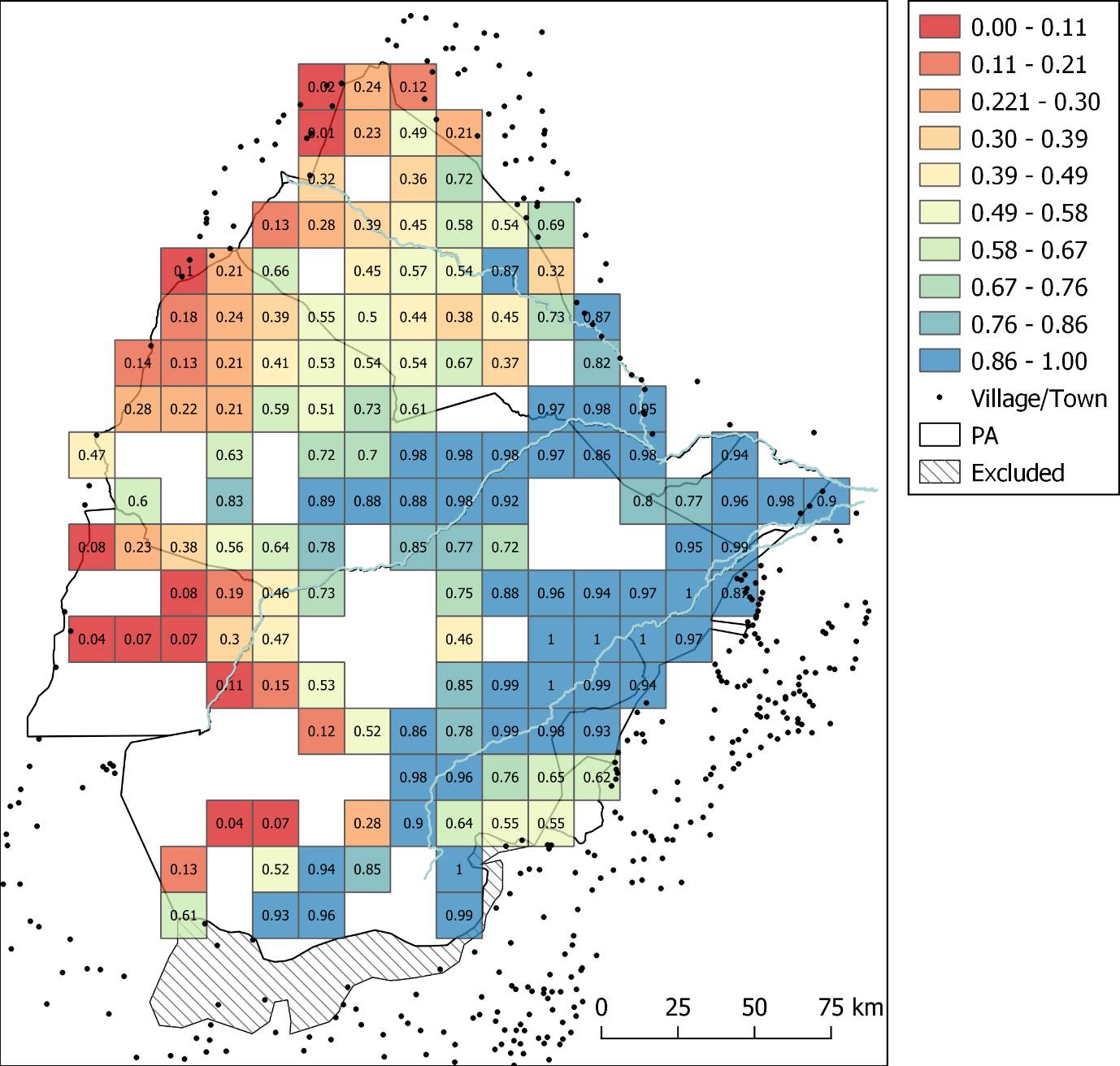
**

***Illegal Human Activity***

*Illegal human activity probability of site use*

**
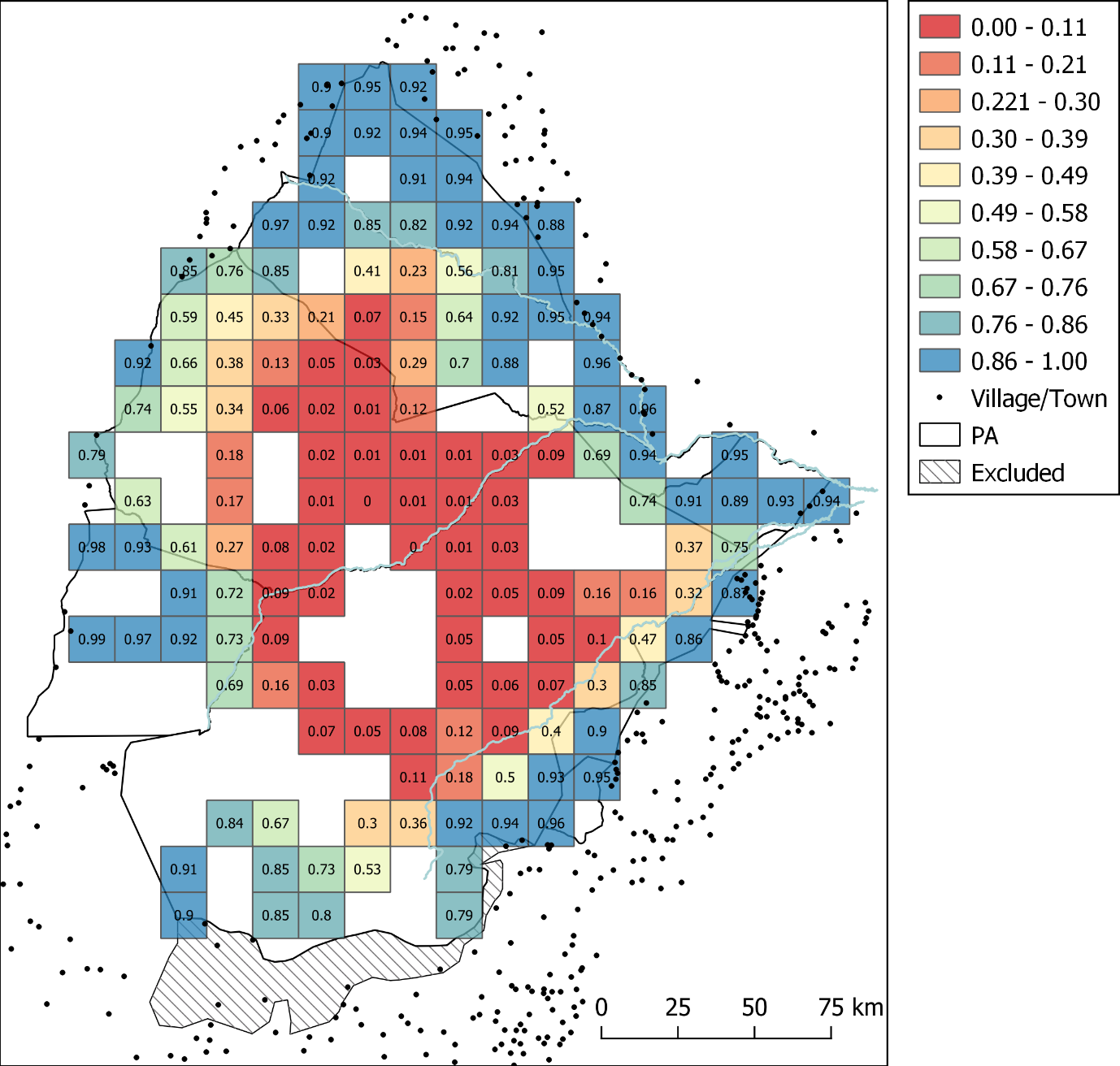
**

***Large Carnivores***

*Lion probability of site use*

**
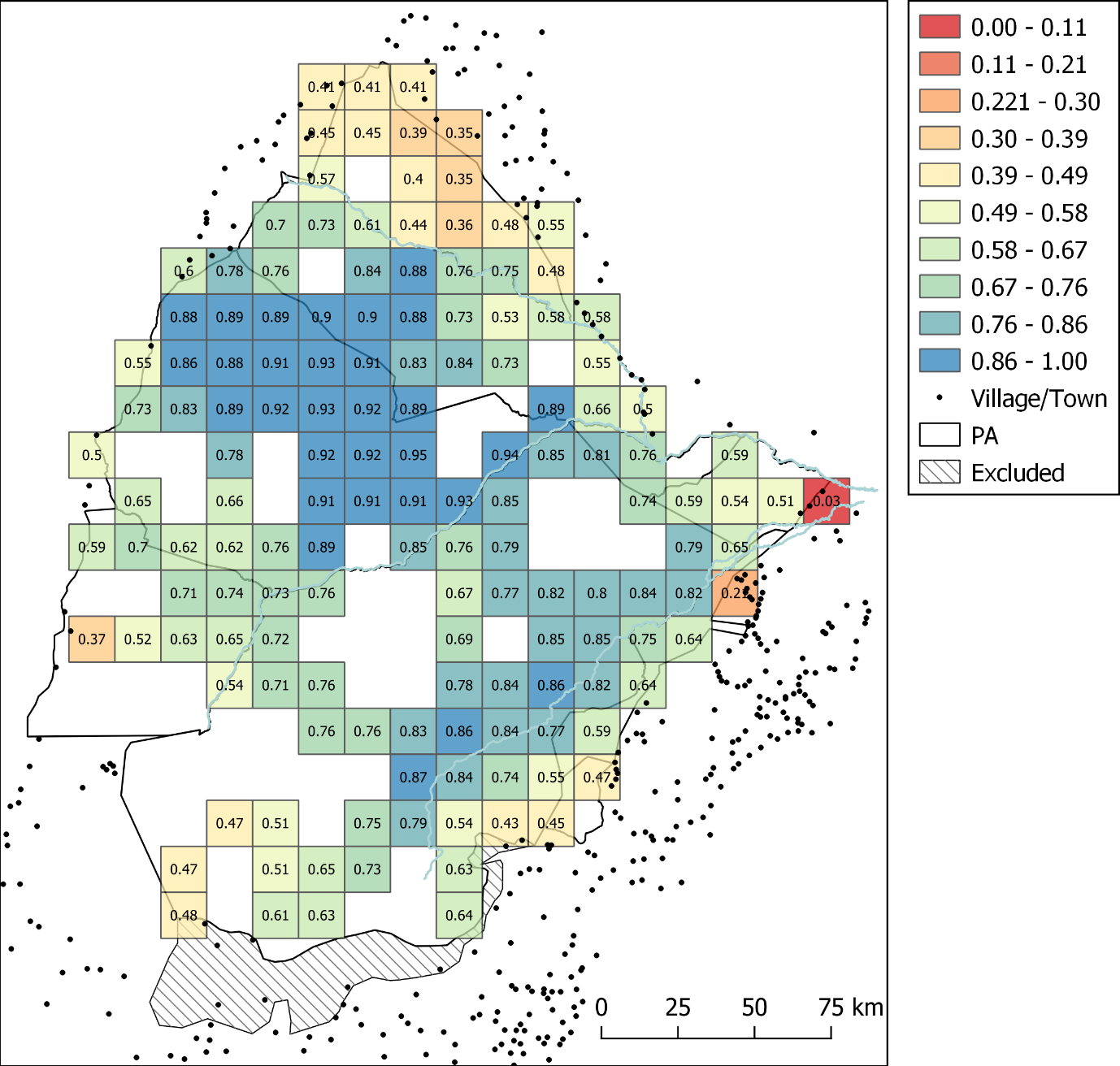
**

*Leopard probability of site use*

**
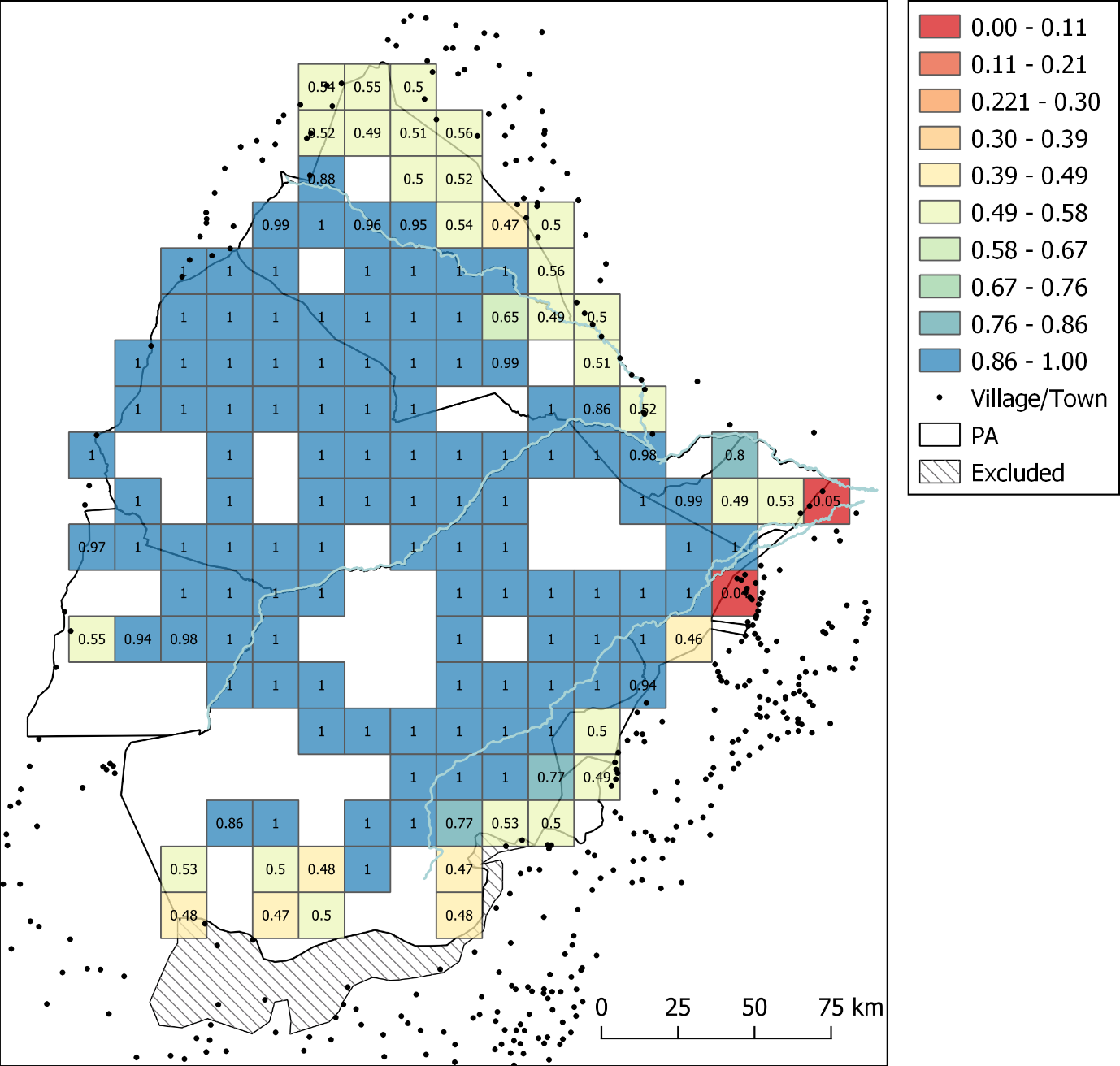
**

*Cheetah probability of site use*

**
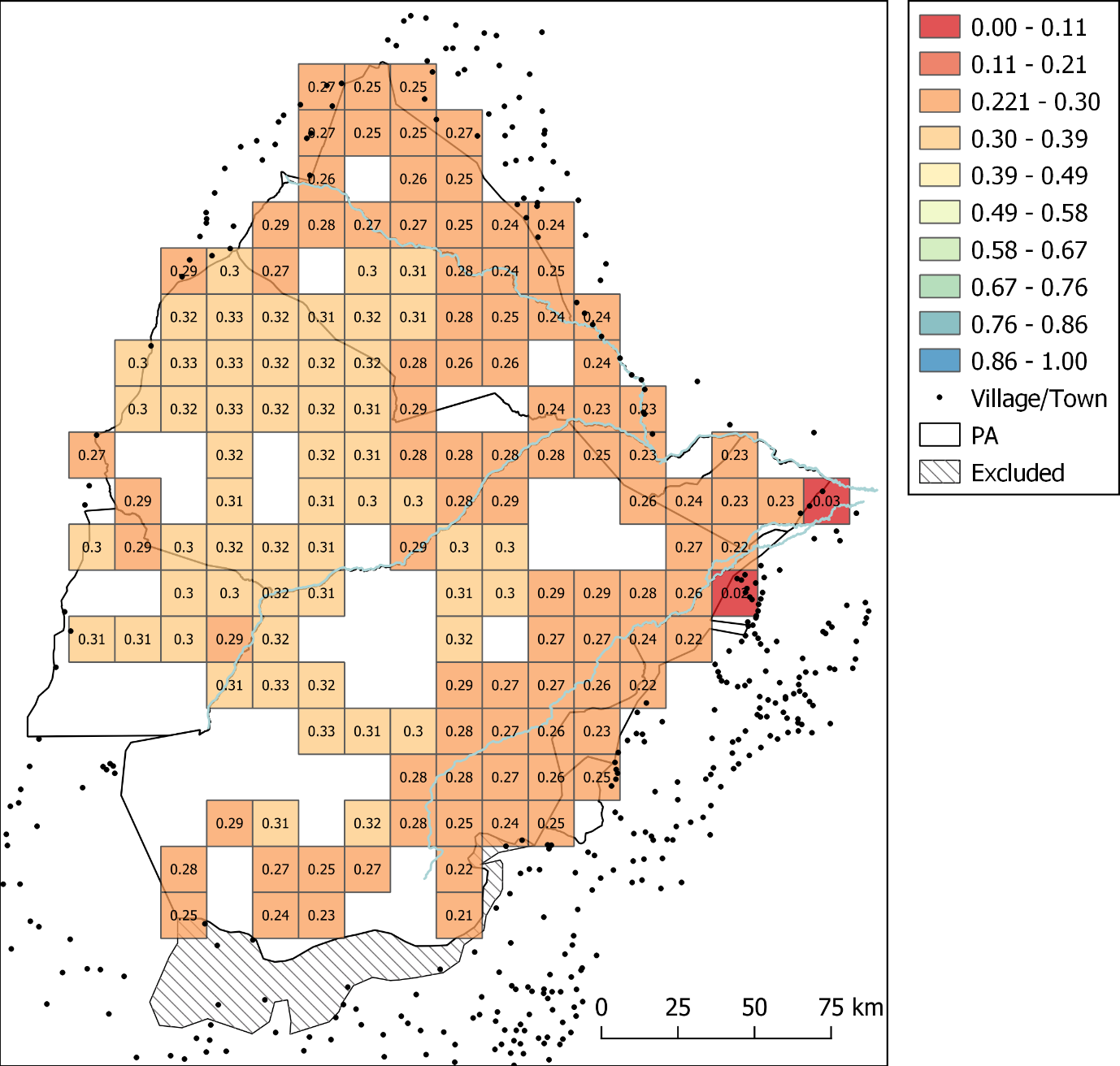
**

*African wild dog probability of site use*

**
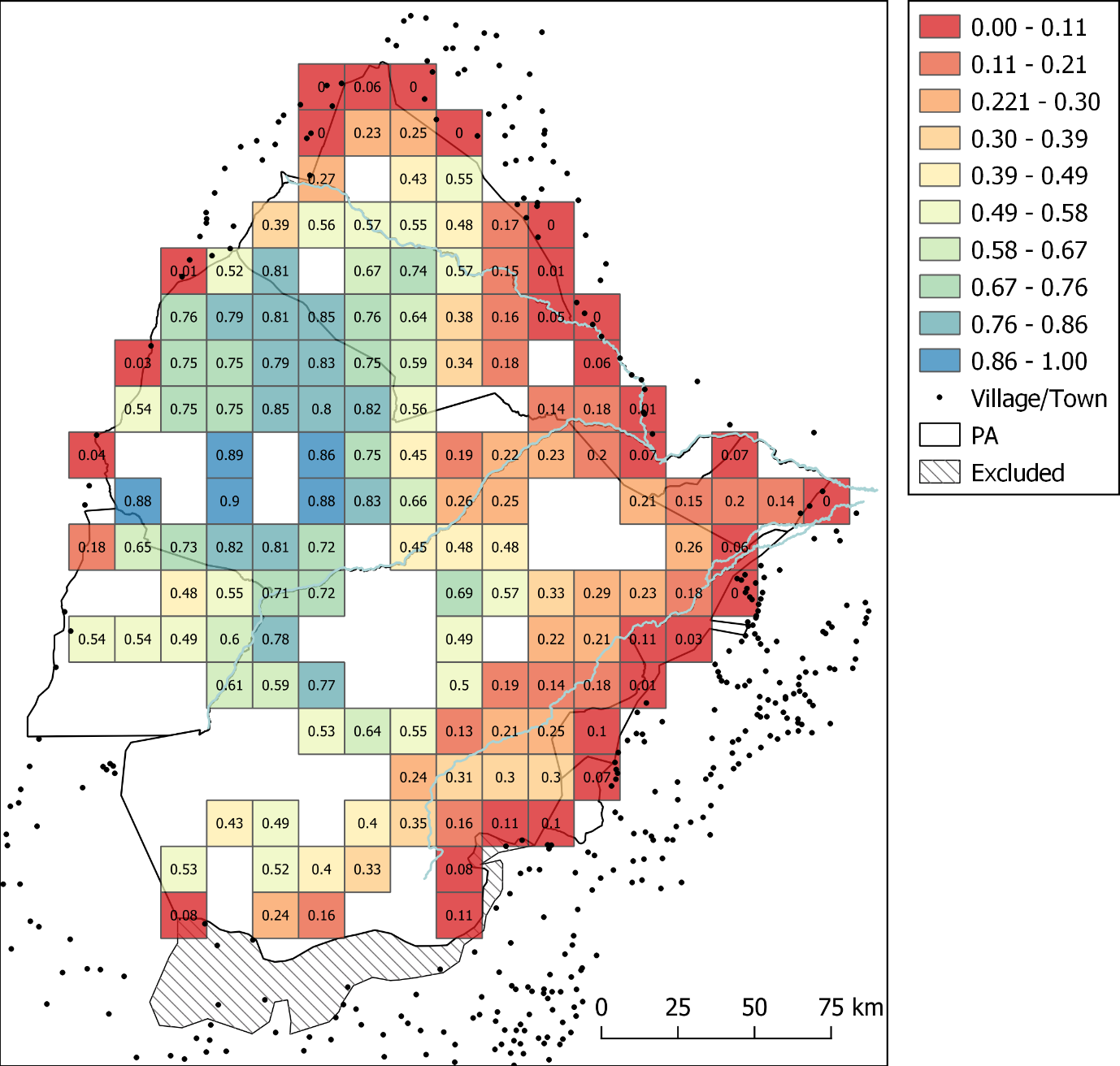
**

**Spoor & Survey Photographs**

Spoor (tracks) of the different large carnivore species recorded during surveying:


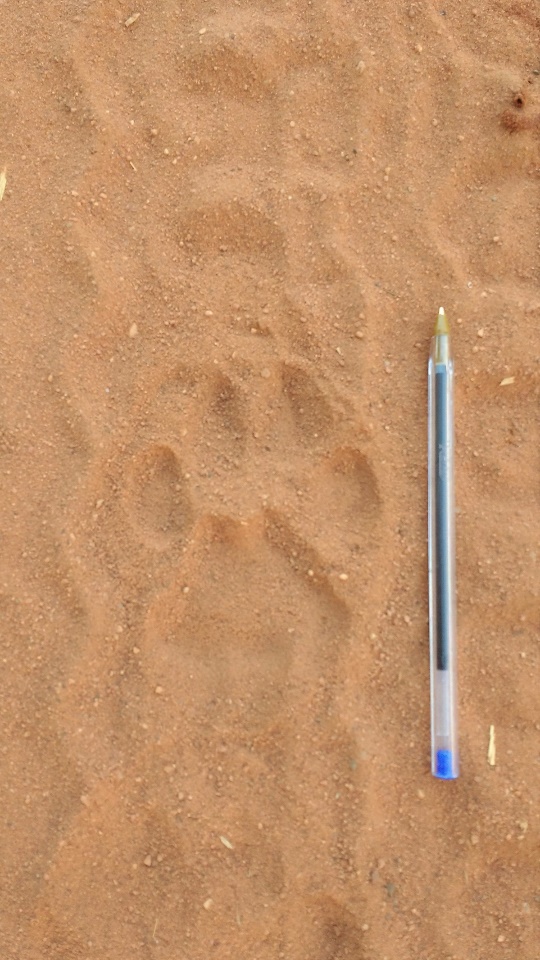


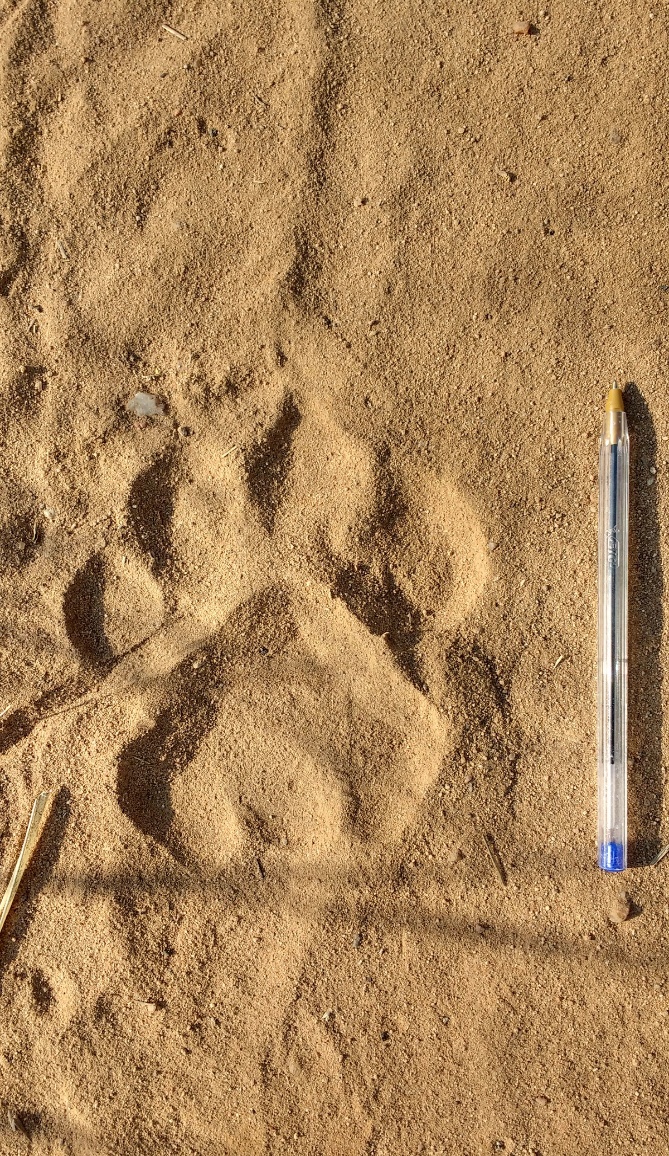


**Lion**


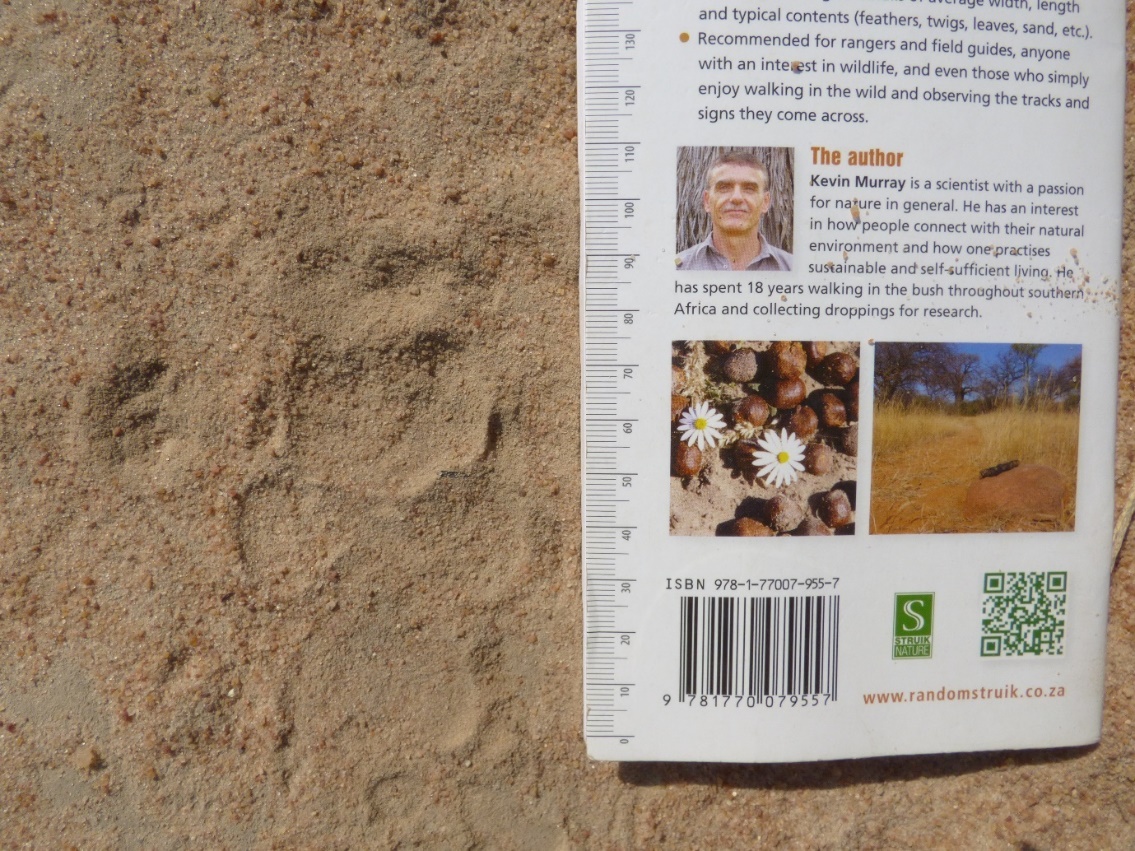


**African**

**Wild Dog**


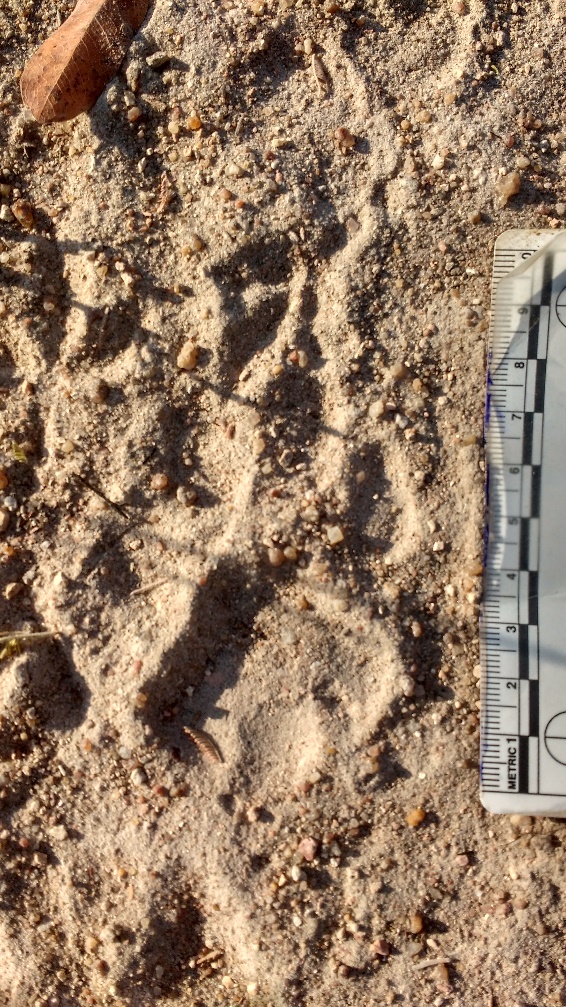


**Cheetah**

**Leopard**


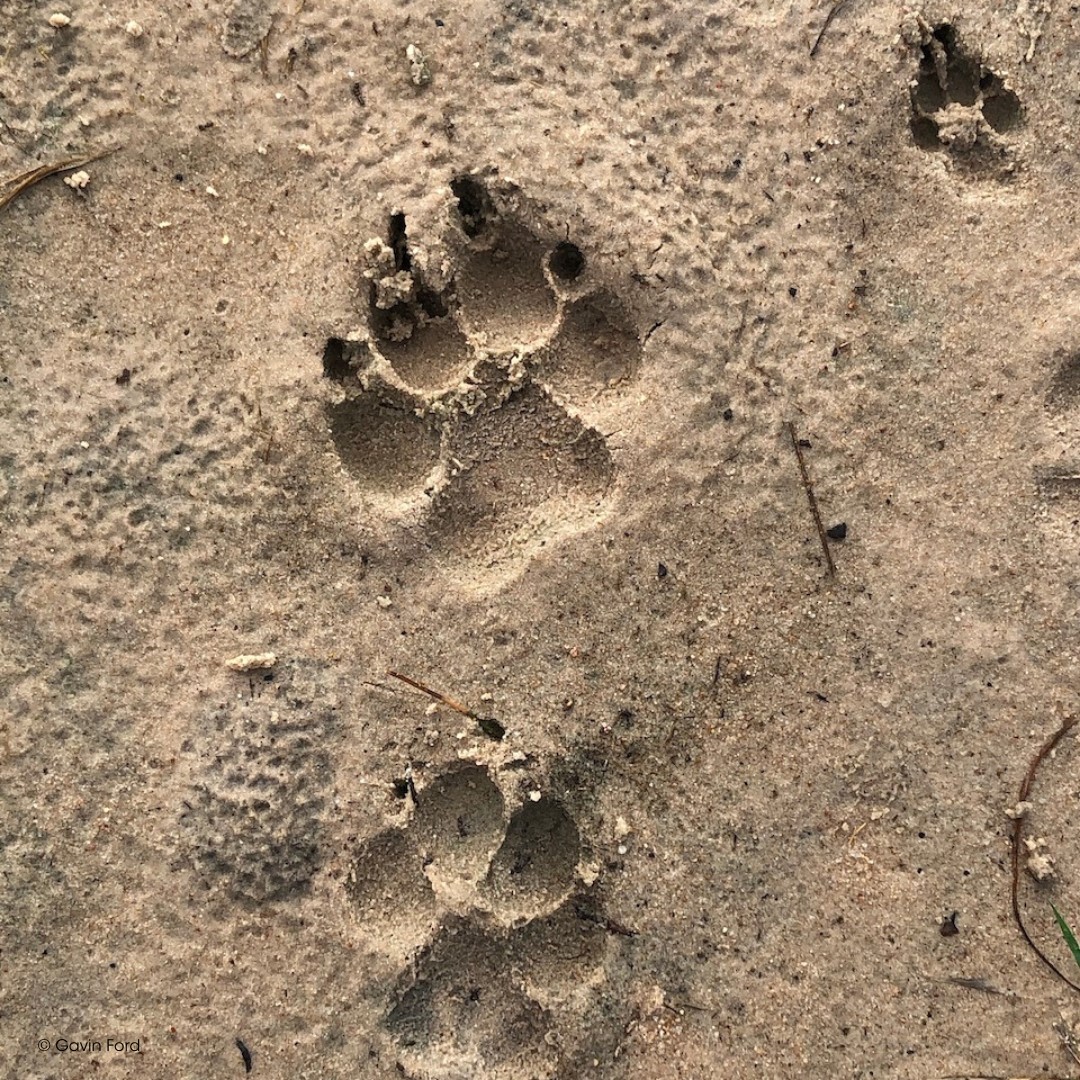


**Spotted**

**Hyaena**

Vehicle setup during survey transects:


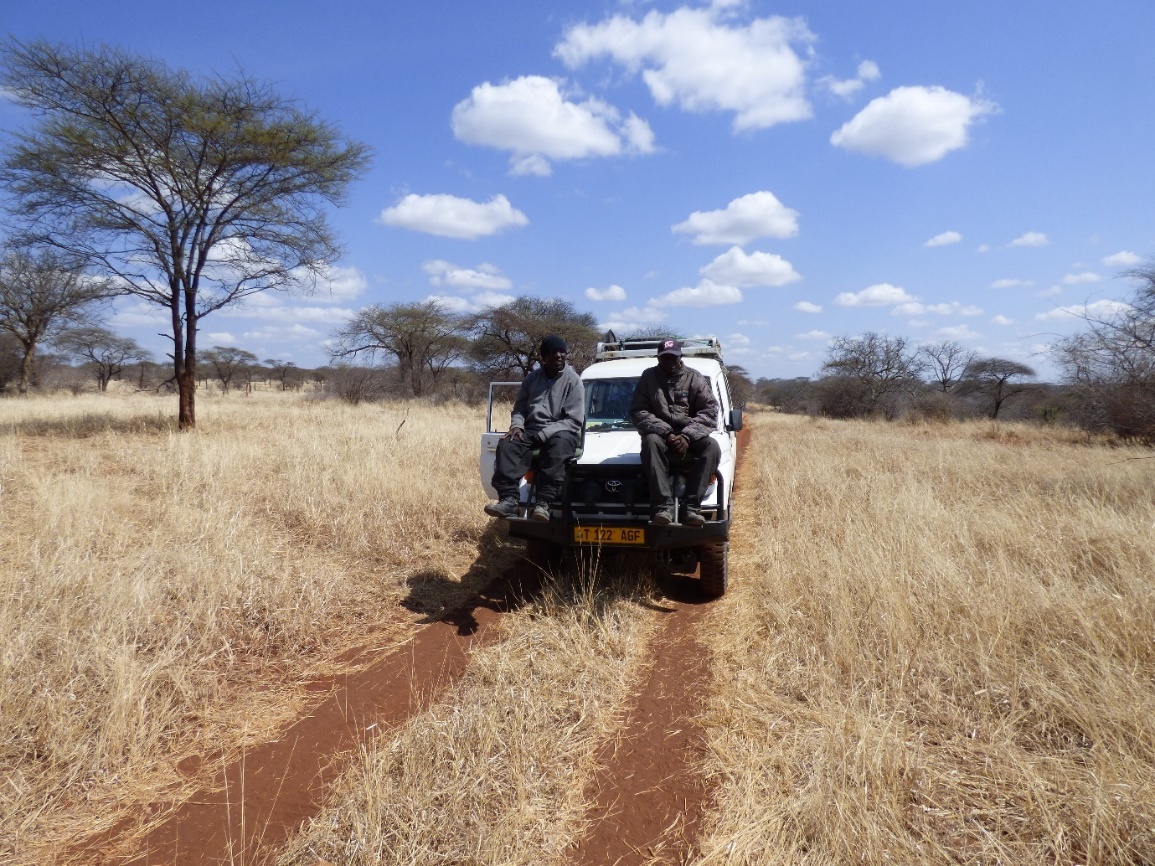


**
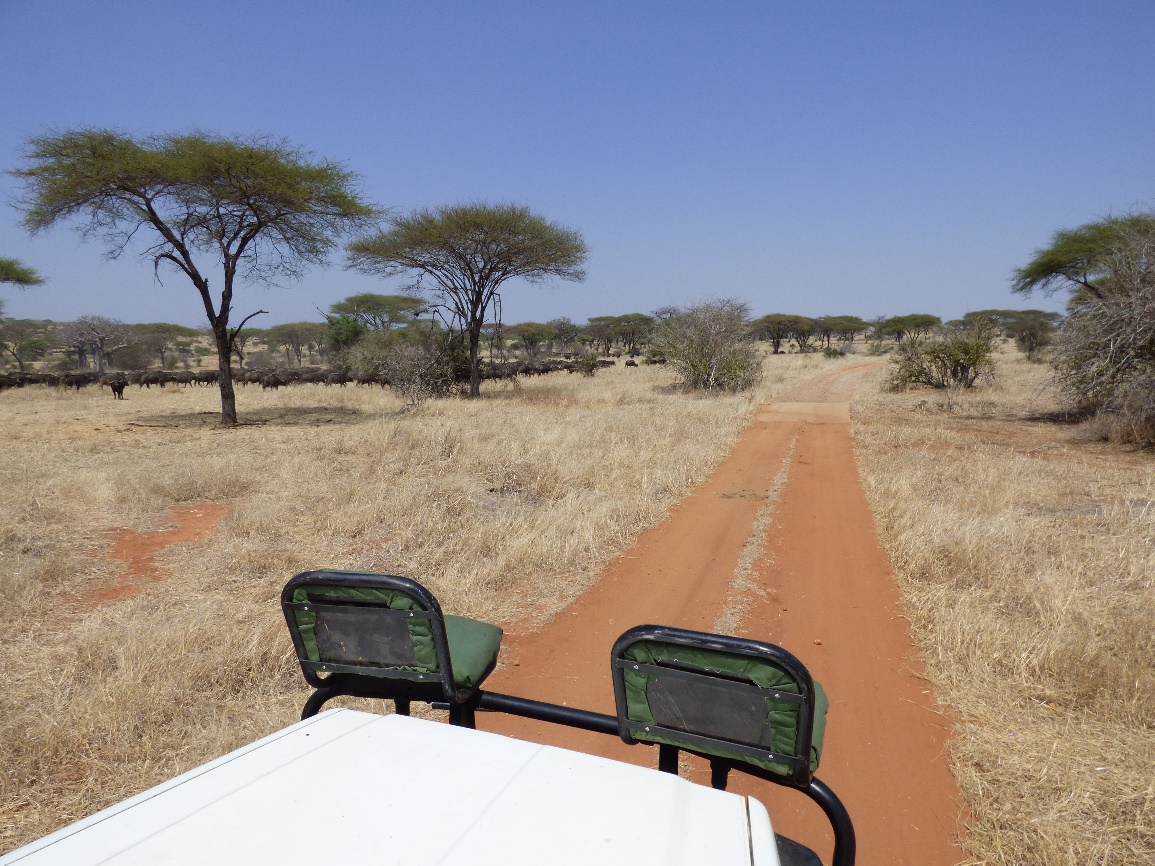
**
